# Cross-species TNF-α signaling in mosquitoes promotes malaria transmission

**DOI:** 10.1038/s44319-026-00929-8

**Published:** 2026-09-29

**Authors:** Meilin Li, Biao He, Jian Zhang, Yongling Fan, Jianyong Li, Feng Zhu, Shuai Guo, Taoli Zhou, Yuanli Gao, Taiping Liu, Wenyue Xu

**Affiliations:** 1 Department of Pathogenic Biology, Army Medical University (Third Military Medical University), Chongqing, China; 2 Key Laboratory of Extreme Environmental Medicine, Ministry of Education of China, Chongqing, China

**Keywords:** Immunology, Microbiology, Virology & Host Pathogen Interaction, Signal Transduction

## Abstract

The interactions between innate immune responses in mammals and insects on vector-borne disease transmission are poorly understood. This study reveals that host-derived tumor necrosis factor α (TNF-α) binds to the mosquito receptor Wengen (Wgn), promoting the transmission of both rodent and human malaria. This TNF-α/Wgn signaling acts through mosquito TNF receptor-associated factor 3 (mTRAF3), which inhibits mosquito immune deficiency (IMD) pathway-related molecules by targeting transforming growth factor-β-activated kinase 1 (mTAK1), leading to decreased thioester-containing protein 1 (TEP1) production and increased parasite infection in mosquitoes. Additionally, neutralizing host-derived TNF-α by an anti-TNF-α antibody prevents experimental cerebral malaria (ECM) and enhances the effectiveness of an anti-Pfs25 antibody in blocking transmission. Our findings uncover a novel strategy by which host-derived TNF-α can be utilized by the parasite to facilitate its transmission, offering new avenues for preventing cerebral malaria and reducing malaria transmission.

## Synopsis

Host TNF-α absorbed by mosquito midguts enhances malaria transmission. This cross-species effect of TNF-α is mediated by the suppression of immune deficiency (IMD) pathway molecules and TEP1.

- Host-derived TNF-α facilitates transmission of both rodent and human malaria parasites.
- Host-derived TNF-α promotes malaria transmission by suppressing mosquito TEP1 expression.
- Host-derived TNF-α downregulates IMD-pathway associated molecules by preferentially binding to the mosquito TNF-α receptor ortholog Wgn.
- TNF-α neutralization prevents ECM development and improves the transmission blocking efficacy of anti-Pfs25 antibodies.

## Abstract (web summary)

Host TNF-α absorbed by mosquito midguts enhances malaria transmission. This cross-species effect of TNF-α is mediated by the suppression of immune deficiency (IMD) pathway molecules and TEP1.

## Graphical abstract

**Figure:**
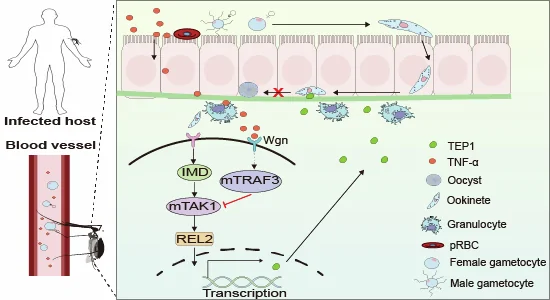

## Introduction

The innate immune systems of mammals and insects share a common evolutionary origin, leading to fundamental similarities in their mechanisms for detecting and responding to pathogens (Hoffmann and Reichhart, 2002). For example, orthologs of mammalian signaling pathways, including TLR (Toll-like receptor) (Lemaitre et al, 1996), Janus kinase (JAK)-signal transducer and activator of transcription (STAT) (Dostert et al, 2005), and tumor necrosis factor-alpha (TNF-α) (Igaki et al, 2002), as well as innate immune defenses, such as RNA interference (RNAi) (Wang et al, 2006), autophagy (Shelly et al, 2009), and complement C3 (Blandin et al, 2004), have been identified in insects. Vector-borne diseases are significant infectious diseases transmitted through the bites of insects like ticks and mosquitoes. During natural transmission, innate immune effectors induced in the host are taken up into the midgut of the insects along with the pathogens (Rana et al, 2023). Consequently, the biting of infected patients by mosquitoes presents a unique opportunity for the interaction of host immune effectors with insect innate immunity within the insects’ midguts (Smith et al, 2016; Rana et al, 2023). However, little is known about the cross-species effects of host-derived immune effectors on mosquito-borne diseases.

Malaria, one of the mosquito-borne diseases, is transmitted when infected female *Anopheles* mosquitoes bite individuals. After biting, microgametocytes and macrogametocytes ingested by the mosquito transform into gametes, which then fertilize to form zygotes. These zygotes develop into ookinetes, which penetrate the midgut epithelium and transform into oocysts within the first 24 h of blood ingestion. Once the oocysts mature within 2 weeks, sporozoites are released into the hemolymph and invade the salivary glands (Beier, 1998). After an infected mosquito bites a healthy individual, sporozoites are introduced into the skin, causing infection. Therefore, blocking the development of the parasite in mosquitoes presents a promising strategy for controlling and potentially eliminating malaria worldwide.

In mosquitoes, the development of parasites is significantly constrained by the mosquito’s innate immune defenses (Barletta et al, 2025). The robust immune response is activated when ookinetes penetrate the midgut epithelial cells (Dimopoulos et al, 2001). This invasion induces the expression of nitric oxide synthase, leading to the production of nitric oxide in the midgut epithelial cells, which effectively kills the ookinetes (Dimopoulos et al, 1998). Concurrently, both heme peroxidase (HPX2) and nicotinamide adenine dinucleotide phosphate oxidase 5 (NOX5) are upregulated through the Jun-N-terminal kinase (JNK) signaling pathway in the midgut epithelial cells, facilitating epithelial nitration (Oliveira et al, 2012). This nitration triggers associated hemocytes to release microvesicles, which subsequently activate complement-like thioester-containing protein 1 (TEP1) to lyse the parasites (Castillo et al, 2017). Additionally, the parasites can activate the peroxidase/oxidase system within the midgut lumen to assemble a dityrosine network that reduces gut permeability to immune elicitors (Kumar et al, 2010). Developing oocysts also stimulate the midgut stem cells to initiate a cellular defense response that limits parasite infection (Barletta et al, 2024). Alongside local immune responses, ookinete invasion triggers a systemic immune response in mosquitoes by inducing the midgut epithelium to secrete prostaglandins, which in turn stimulate the production of hemocyte differentiation factors to promote hemocyte differentiation (Barletta et al, 2019; Rodrigues et al, 2010). Signaling pathways such as the Toll pathway, JAK-STAT pathway, and the immune deficiency (IMD) pathway are also involved in the mosquito’s immune defense against malaria parasites (Garver et al, 2012; Gupta et al, 2009; Frolet et al, 2006; Garver et al, 2009), and Toll and IMD signaling pathways regulate TEP1 expression primarily dependent on Relish-like protein (REL)1 and REL2 members of the NF-kB transcription factors, respectively (Frolet et al, 2006).

In the mammalian host, malaria blood-stage infection induces innate immune cells to produce proinflammatory cytokines, including IL-1β, IL-6 and TNF-α (Coban et al, 2005; Krishnegowda et al, 2005). However, excessive production of TNF-α is strongly associated with the development of severe malaria, such as cerebral malaria (Kwiatkowski et al, 1990). TNF-α signals through two receptors, TNFR1 and TNFR2, with TNFR2 activation being primarily responsible for upregulating intracellular adhesion molecules on endothelial cells, contributing to the development of experimental cerebral malaria (ECM) (Lucas et al, 1997). Interestingly, orthologs of TNF-α and its receptor, referred to as Eiger and Wgn (Wengen)/Grnd (Grindelwald), have been identified in *Drosophila* (Moreno et al, 2002; Kauppila et al, 2003; Palmerini et al, 2021) and *Anopheles* mosquitoes (Samantsidis et al, 2025; Srinivasan et al, 2022). Since innate immune effectors, including host-derived TNF-α, could be taken up into the midgut of mosquitoes along with parasites during natural malaria transmission, we wondered whether host-derived TNF-α could interact with Wgn/Grnd in mosquitoes to modulate malaria transmission in a cross-species manner. Our results demonstrate that host-derived TNF-α can selectively bind and activate the Wgn signaling pathway, thereby inhibiting the IMD pathway-related molecules and reducing TEP1 expression, which in turn promotes the transmission of both rodent and human malaria parasites.

## Results

### Host-derived TNF-α exacerbates malaria pathogenesis and facilitates parasite transmission

**Figure 1:**
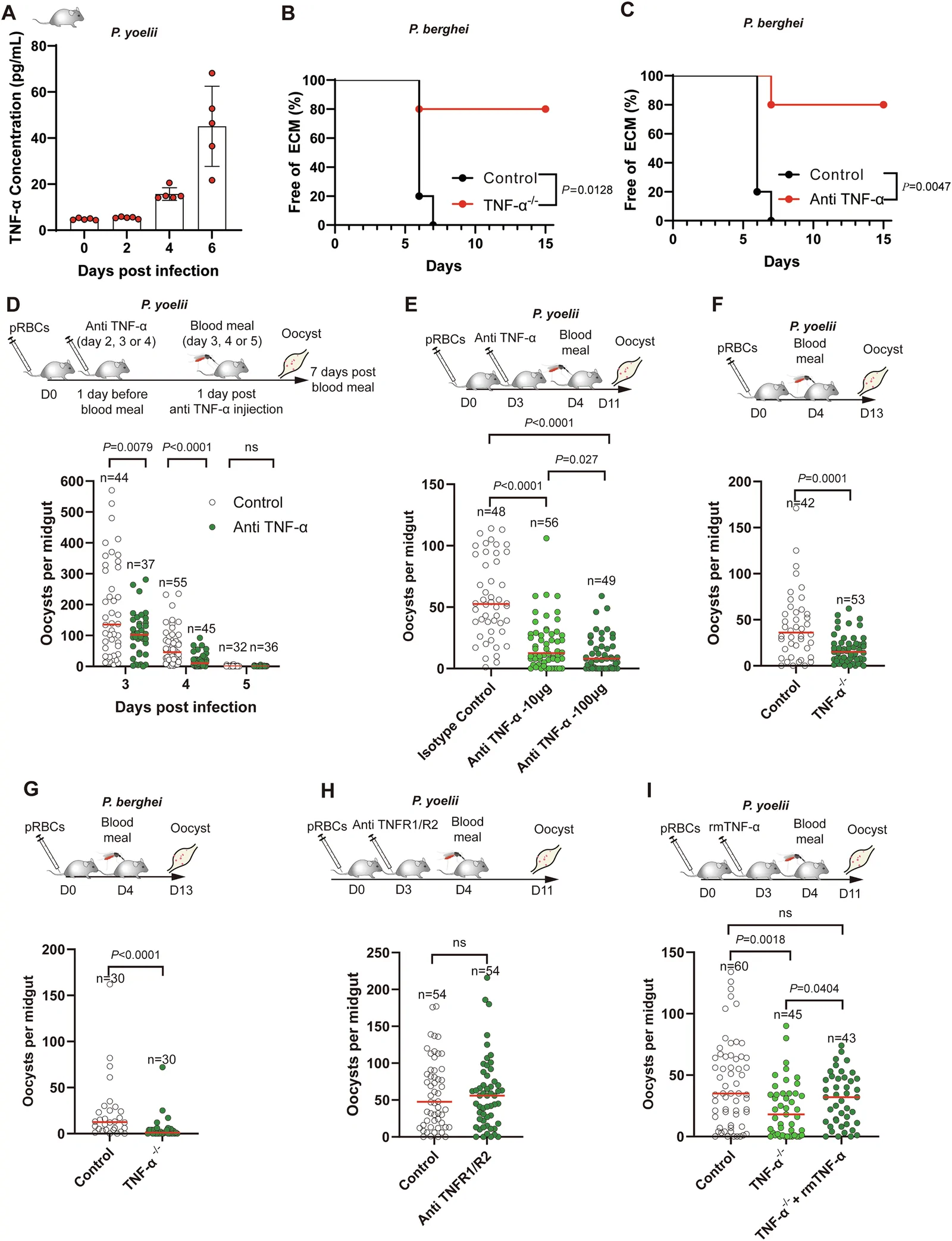
Host-derived TNF-α exacerbates malaria pathogenesis and facilitates parasite transmission. (**A**) The concentration of TNF-α in the serum of *P. yoelii*-infected mice was dynamically monitored by cytometric bead array (CBA) at the indicated days post-infection. Results are from *n* = 5 biological replicates. (**B**) The percentage of *P. berghei*-infected littermate controls and TNF-α^−/−^ mice that were free of ECM are shown. Results are from *n* = 5 biological replicates. (**C**) *P. berghei*-infected mice were treated with or without anti-TNF-α antibody, and the percentage of mice that were free of ECM was recorded. Results are from *n* = 5 biological replicates. (**D**) *P. yoelii*-infected mice were treated with or without anti-TNF-α antibody on days 2, 3, or 4, then were exposed to mosquitoes. The oocyst number in midguts of mosquitoes was determined at 7 days post blood meal. (**E**) Oocyst counts in midguts of mosquitoes fed on *P. yoelii*-infected mice pre-treated with or without 10 or 100 μg of anti-TNF-α antibody. (**F**, **G**) Oocyst counts in midguts of mosquitoes fed on control or TNF-α^−/−^ mice infected with *P. yoelii* (**G**) or *P. berghei* (**H**) for 4 days. (**H**) Oocyst counts in midguts of mosquitoes fed on *P. yoelii*-infected mice pre-treated with or without anti-TNFR1 and TNFR2 antibodies. (**I**) Control and TNF-α^−/−^ mice were infected with *P. yoelii*. Four days later, the infected TNF-α^−/−^ mice were supplemented with rmTNF-α, and all mice were then exposed to mosquitoes. Data information: Data in panels (**A**) are presented as the mean ± SD. In (**D**–**I**), the number of oocysts per mosquito is represented by individual dots, with a horizontal line indicating the median, and n indicating the number of biologically independent mosquito samples. Statistical analysis for (**A**) was done by unpaired *t* test (two-tailed), and (**B**, **C**) by the log-rank (Mantel–Cox) test, while (**D**–**I**) were analyzed using Mann–Whitney *U* test (two-tailed). ns no significance. Source data are available online for this figure.

**Figure EV1:**
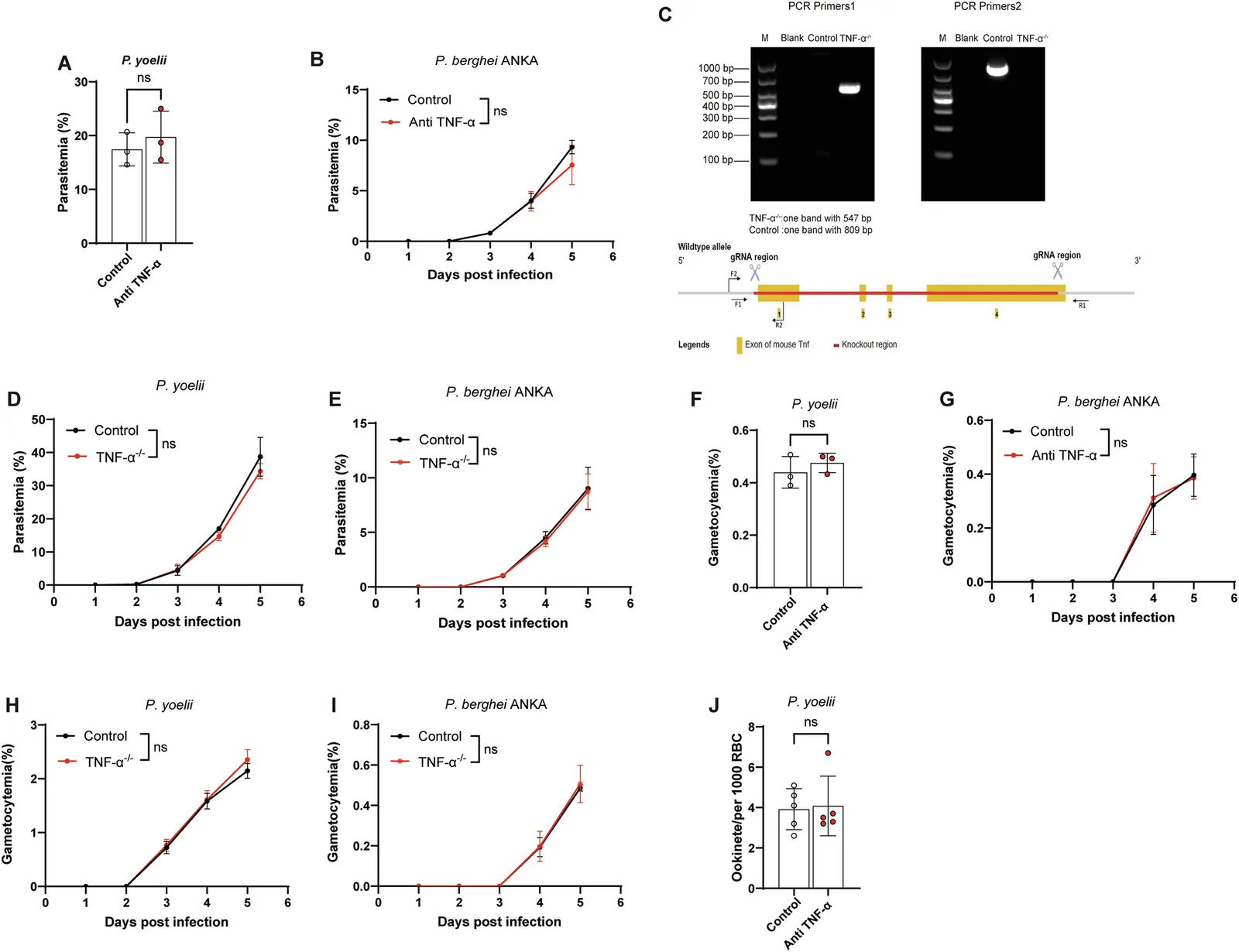
Effect of TNF-α on parasite growth in mice. (**A**) Parasitemia of *P. yoelii* in mice pre-treated with or without anti-TNF-α antibody at 4 days post-infection. Results are from *n* = 3 biological replicates. (**B**) Parasitemia of *P. berghei* in mice pre-treated with or without anti-TNF-α. Results are from *n* = 5 biological replicates. (**C**) Identification of TNF-α knockout mice. Different lengths of fragments amplified from the genomic DNA extracted from control and transgenic mice. A 547 bp target fragment was obtained from the genomic DNA extracted from TNF-α knockout mice while no band was obtained from the genomic DNA extracted from control mice with Primers 1. A 809 bp target fragment was obtained from the genomic DNA extracted from control mice while no band was obtained from genomic extracted from TNF-α knockout mice with Primers 2. (**D**, **E**) Parasitemia of *P. yoelii* (**D**) and *P. berghei* (**E**) in control and TNF-α^−/−^ mice Results are from *n* = 3 biological replicates. (**F**) Gametocytemia of *P. yoelii* in mice pre-treated with or without anti-TNF-α antibody at 4 days post-infection. Results are from *n* = 3 biological replicates. (**G**) Gametocytemia of *P. berghei* in mice pre-treated with or without anti-TNF-α antibody. Results are from *n* = 5 biological replicates. (**H**, **I**) Gametocytemia of *P. yoelii* (**H**) and *P. berghei* (**I**) in control and TNF-α^−/−^ mice. Results are from *n* = 3 biological replicates. (**J**) Blood taken from *P. yoelii*-infected mice pre-treated with or without anti-TNF-α antibody was in vitro cultured for 24 h, and the number of generated ookinetes per 1000 RBCs was counted and compared. Results are from *n *= 5 biological replicates. Data information: (**A**, **B**, **D**–**J**) Data are presented as the mean ± SD. Statistical analysis of data in (**A**, **F**, **J**) was performed by unpaired *t* test (two-tailed), while data in (**B**, **D**, **E**, **G**–**I**) was analyzed by two-way ANOVA mix model. ns no significance. Source data are available online for this figure.

While interferon-gamma (IFN-γ) is widely recognized as a key immune effector in controlling malaria infection (Su and Stevenson, 2000), the roles of other inflammatory cytokines in malaria control remain poorly understood. In this study, we observed that infection with the rodent malaria parasite *Plasmodium yoelii* BY265 (*P. yoelii*) significantly elevated the level of TNF-α in the serum of infected mice (Fig. 1A). However, neutralization of TNF-α using anti-TNF-α antibody had no significant effect on the blood-stage parasitemia of either *P. yoelii* or *Plasmodium berghei* ANKA (*P. berghei*) (Fig. EV1A,B), a finding that was consistent in TNF-α^−/−^ mice (Fig. EV1C–E). Remarkably, the development of ECM was greatly prevented in TNF-α^−/−^ mice infected with *P. berghei* (Fig. 1B). Additionally, pre-treatment with anti-TNF-α antibody also efficiently prevented the onset of ECM in these mice (Fig. 1C). Thus, TNF-α has no significant effect on malaria development in the host, but significantly promotes ECM development.

In addition to its impact on ECM development, the role of host-derived TNF-α in modulating parasite transmission through mosquito vectors has also been largely unexplored. To further investigate the functional significance of TNF-α in transmission dynamics, we neutralized TNF-α with anti-TNF-α antibody in *P. yoelii*-RFP-infected mice prior to mosquito feeding. Notably, TNF-α neutralization led to a significant reduction in malaria infection intensity (measured by oocyst counts), when mosquitoes fed on mice infected with *P. yoelii*-RFP for either 3 or 4 days (Fig. 1D). Increasing the amount of anti-TNF-α antibody from 10 μg to 100 μg further suppressed malaria infection in mosquitoes (Fig. 1E), indicating a dose-dependent relationship. Similar results were observed when mosquitoes fed on TNF-α^−/−^ mice infected with *P. yoelii* or *P. berghei* (Fig. 1F,G). Furthermore, the neutralization of both TNFR1 and TNFR2 had no significant effect on malaria transmission in mosquitoes (Fig. 1H), but the introduction of recombinant mouse TNF-α (rmTNF-α) in TNF-α^−/−^ mice restored malaria infection in mosquitoes (Fig. 1I), indicating the direct role of host-derived TNF-α in facilitating malaria infection in mosquitoes. Notably, the neutralization of TNF-α or the infection of TNF-α^−/−^ mice with either *P. yoelii* or *P. berghei* did not significantly affect gametocytemia in the mice (Fig. EV1F–I), nor did it impact the capacity of gametocytes to develop into ookinetes in vitro (Fig. EV1J). This suggests that the promoting effect of TNF-α on malaria infection primarily operates through its influence on the mosquito rather than on the host.

### Host-derived TNF-α has no impact on either the attractiveness of hosts to mosquitoes or mosquito fitness

**Figure EV2:**
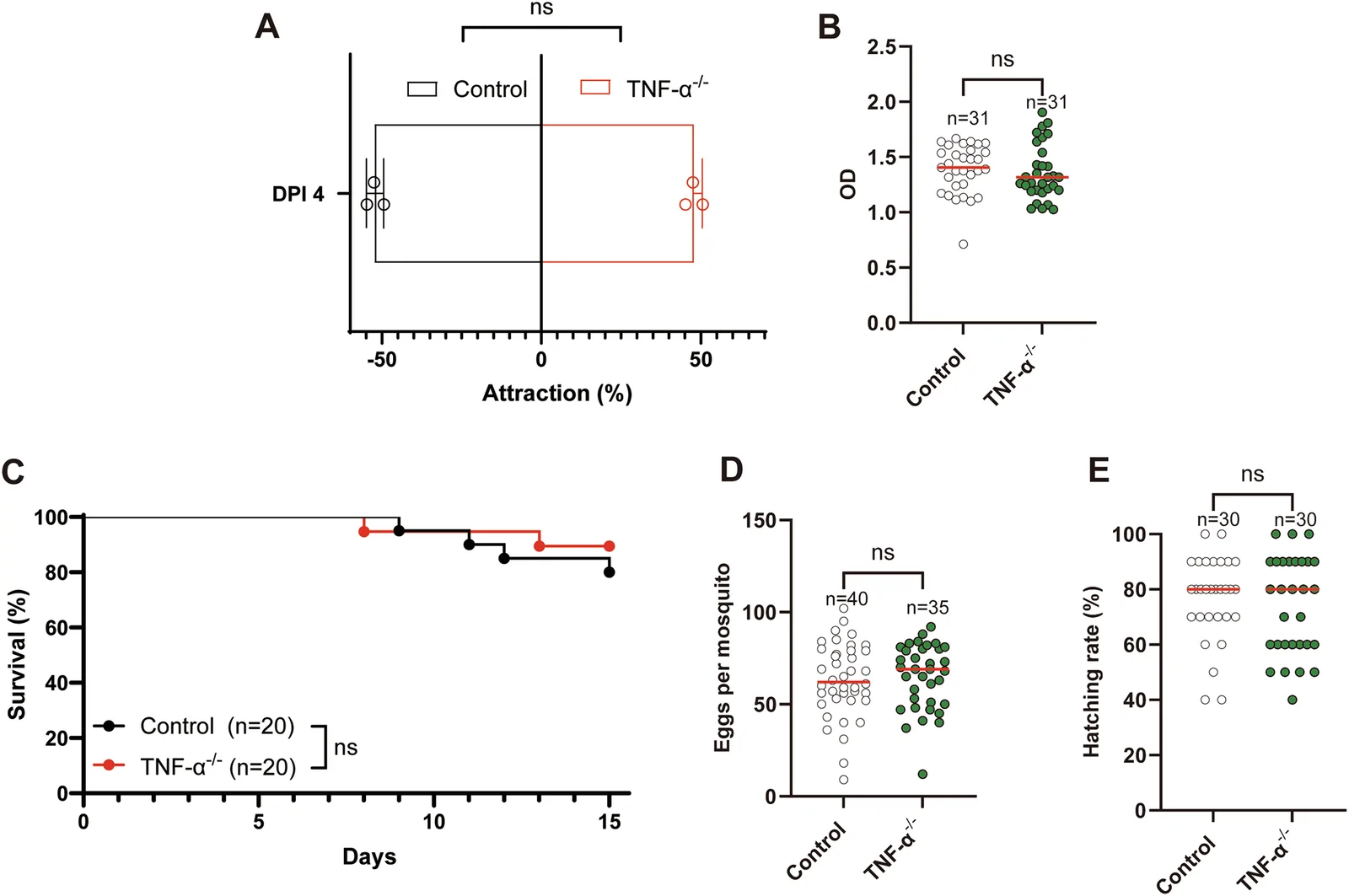
Effect of TNF-α on mosquito fitness. (**A**) Host-seeking behavior of mosquitoes toward littermate control or TNF-α^−/−^ mice infected with *P. yoelii* for 4 days was assessed through dual-choice olfactometry assays. A box plot shows the choice preference of the mosquitoes in Control and TNF-α^−/−^ groups. The central line represents the median (50th percentile). The box boundaries represent the 25th and 75th percentiles (Q1 and Q3, interquartile range, IQR). The whiskers extend to the minimum and maximum observed values within 1.5 × IQR from the lower and upper quartiles; since all data points lie within this range, the whisker endpoints coincide with the data extrema and no outliers are present. Specific descriptive values are as follows: Control: Min = 49.4, Q1 = 49.4, Median = 52.5, Q3 = 54.8, Max = 54.8. TNF-α^−/−^: Min = 45.2, Q1 = 45.2, Median = 47.5, Q3 = 50.6, Max = 50.6. Results are from *n* = 3 biological replicates. (**B**) Midguts of mosquitoes fed on infected control or TNF-α^−/−^ mice were dissected. Hemoglobin-derived heme levels were quantified by measuring absorbance at 410 nm. Individual dots represent the OD value of ten midguts, with a line indicating median. (**C**) Survival probability of mosquitoes fed on infected control or TNF-α^−/−^ mice within 15 days. (**D**) The number of eggs laid by each mosquito fed on infected control or TNF-α^−/−^ mice. Individual dots represent the number of eggs laid by per mosquito, with a line indicating the median. (**E**) The percentage of hatched eggs collected from mosquitoes fed on infected control or TNF-α^−/−^ mice. Individual dots represent the hatching rate of ten eggs, with a line indicating the median. Results are from *n* = 30 biological replicates. Data information: For (**B**–**D**), *n* indicates the number of biologically independent mosquito samples. Statistical analysis of data in (**A**) was performed by Fisher’s exact test (two-sided), while data in (**B**, **D**, **E**) was analyzed by Mann–Whitney *U* test (two-tailed). In panel (**C**), data was analyzed by log-rank (Mantel–Cox) test, ns: no significance. Source data are available online for this figure.

To explore the underlying mechanisms of TNF-α-promoted malaria transmission, we utilized a *P. yoelii* infection model in the following sections, unless a different species is specified. As the acquirement of blood from infected mice is the first critical step in malaria transmission, we sought to determine whether host-derived TNF-α would influence mosquitoes to take blood from the infected mice. As a result, we found the infected TNF-α^−/−^ mice and littermate control mice had no significant difference in the attractiveness to mosquitoes (Fig. EV2A). In addition, the amount of blood taken by mosquitoes from infected control mice was comparable to that taken from infected TNF-α^−/−^ mice (Fig. EV2B), suggesting that TNF-α did not play a role in enhancing blood-feeding behavior in mosquitoes. The lifespan of mosquitoes that fed on infected control mice was similar to that of mosquitoes that fed on infected TNF-α^−/−^ mice (Fig. EV2C), indicating that TNF-α does not influence the longevity of mosquitoes feeding on the infected mice. There were also no significant differences in the number of eggs laid by mosquitoes or the hatching rates of those eggs between the groups fed on infected control and TNF-α^−/−^ mice (Fig. EV2D,E). Therefore, neither host attraction nor mosquito fitness contributes to the promotion of malaria infection in mosquitoes by host-derived TNF-α.

### Changes in the microbiota do not contribute to TNF-α-mediated promotion of malaria transmission

**Figure EV3:**
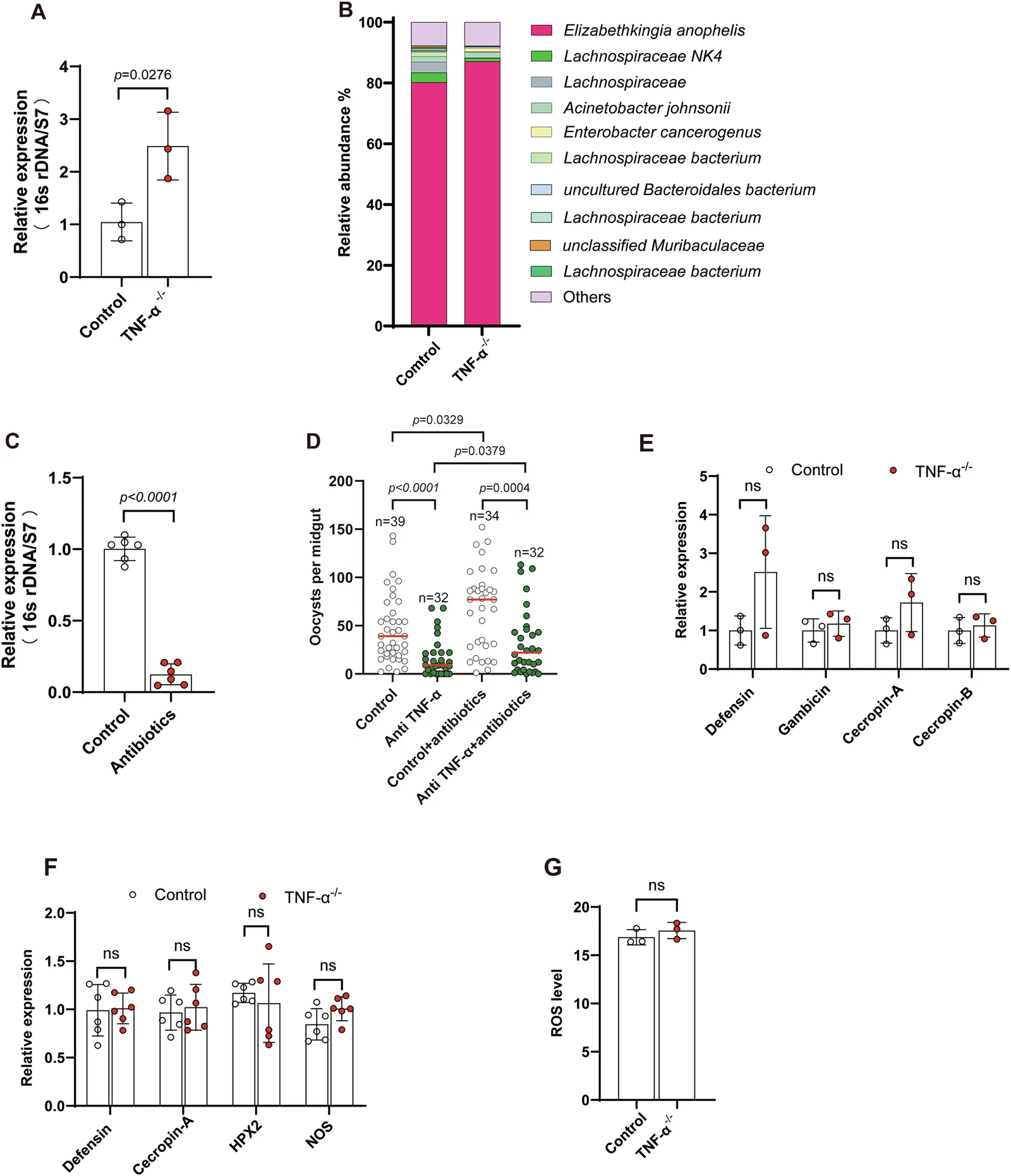
The alteration of microbiota, AMPs and ROS is not contributed to the promoting effect of TNF-α on malaria infection. (**A**) The abundance of microbiota in midguts of mosquitoes feeding on *P. yoelii*-infected control or TNF-α^−/−^ mice was determined by qPCR at 24 h post-blood meal. Results are from *n* = 3 biological replicates. (**B**) Comparative analysis of midgut microbial communities in mosquitoes feeding on infected control and TNF-α^−/−^ mice at 24 h post-blood meal. (**C**) The abundance of microbiota in the mosquito midgut was assessed by qPCR before and after 4-day antibiotic treatment. Results are from *n* = 6 biological replicates. (**D**) After microbiota was cleared by antibiotics treatment, mosquitoes were allowed to feed on *P. yoelii*-infected control and TNF-α^−/−^ mice. Seven days later, the oocysts number in mosquitoes were counted and compared. The number of oocysts per mosquito represented by individual dots, with a horizontal line indicating the median, and *n* indicating the number of biologically independent mosquito samples. (**E**) Relative expression of *Defensin*, *Gambicin, Cecropin-A* and *Cecropin-B* between mosquitoes fed on *P. yoelii*-infected control and TNF-α^−/−^ mice. Results are from *n* = 3 biological replicates. (**F**) Relative expression of *Defensin*, *Cecropin-A*, HPX2 and NOS between hemocytes from mosquitoes fed on *P. yoelii*-infected control and TNF-α^−/−^ mice. Results are from *n* = 6 biological replicates. (**G**) ROS levels in midguts of mosquitoes feeding on *P. yoelii*-infected control and TNF-α^−/−^ mice was quantified by DHE assays 24 h post blood meal. Data were normalized to total protein content (Bradford assay) and expressed as relative fluorescence units (RFU)/μg protein. Results are from *n* = 3 biological replicates. Data information: Data in panels of (**A**, **C**, **E**–**G**) are presented as the mean ± SD. Statistical analysis of data in (**A**, **C**, **E**–**G**) were performed by unpaired *t* test (two-tailed), while data in (**D**) was analyzed by Mann–Whitney *U* test (two-tailed). Source data are available online for this figure.

Ample evidence has shown that the microbiota in the mosquito midgut significantly influences the susceptibility of mosquitoes to malaria parasites (Dong et al, 2009; Cirimotich et al, 2011; Gao et al, 2021; Feng et al, 2022; Huang et al, 2023). Therefore, we next investigated whether TNF-α promoted malaria transmission by modulating the components of the microbiota in the mosquito midgut. To this end, we compared the bacterial burden in the midguts of mosquitoes fed on infected control and TNF-α^−/−^ mice. As expected, TNF-α-deficiency significantly increased the bacterial burden in mosquitoes fed on infected mice (Fig. EV3A). 16S rDNA sequencing of the microbiota in the midguts of mosquitoes revealed a significant alteration in the bacterial community of mosquitoes that fed on infected TNF-α^−/−^ mice (Fig. EV3B). However, TNF-α-deficiency still promoted malaria transmission even after the bacteria in the mosquito midgut were cleared using antibiotics (Fig. EV3C,D). Thus, the promoting effect of TNF-α on malaria infection is not attributed to alterations in the microbiota of the mosquito midgut.

### Host-derived TNF-α enhances malaria infection in mosquitoes by inhibiting TEP1 expression

**Figure 2:**
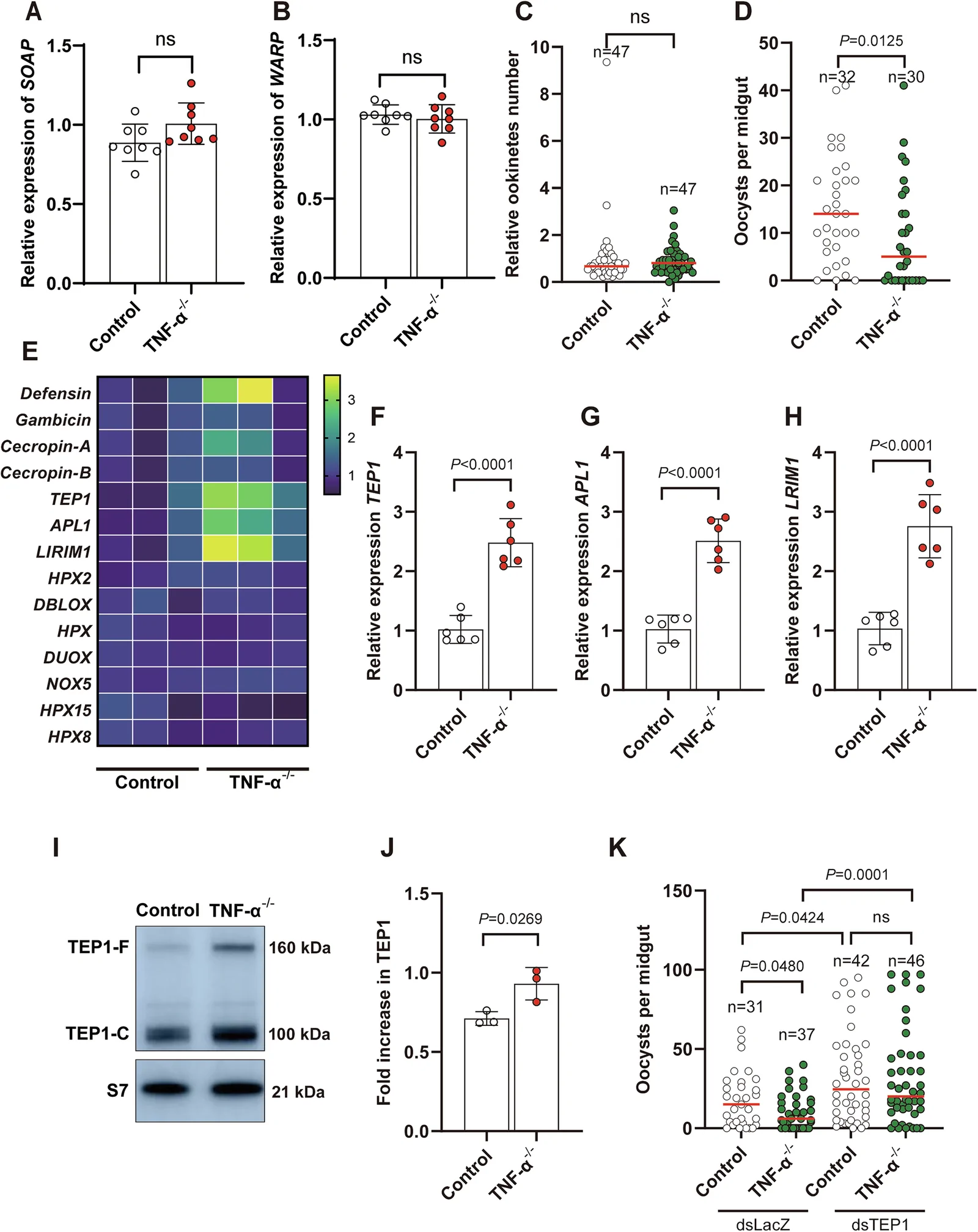
Host-derived TNF-α enhances malaria infection in mosquitoes by inhibiting TEP1 expression. (**A**, **B**) The number of ookinetes in the midgut of mosquitoes that fed on *P. yoelii*-infected control and TNF-α^−/−^ mice at 14 h post-blood meal was quantified by qPCR, measuring the relative expression of *SOAP* (**A**) and *WARP* (**B**) normalized to *S7*. Results are from *n* = 8 biological replicates. (**C**) The average number of ookinetes per mosquito fed on *P. yoelii*-infected control and TNF-α^−/−^ mice was counted. The relative number of ookinetes at 14 h post-blood meal was expressed as the ratio of average number of ookinetes in mosquitoes fed on TNF-α^−/−^ or control mice to that in mosquitoes fed on the control mice. (**D**) Oocyst counts in midguts of mosquitoes fed on the infected control and TNF-α^−/−^ mice at 2 days post-blood meal. (**E**) Heatmap of differentially expressed genes related to antimicrobial peptides, the TEP1 complex, peroxidase/oxidase, and nitration in the midguts of mosquitoes fed on *P. yoelii*-infected control and TNF-α^−/−^ mice at 24 h post-blood meal. (**F**–**H**) Relative expression levels of *TEP1* (**F**), *APL1* (**G**), and *LRIM1* (**H**) in mosquitoes fed on *P. yoelii*-infected control and TNF-α^−/−^ mice at 24 h post-blood meal. Results are from *n* = 6 biological replicates. (**I**, **J**) Western blot analysis detecting TEP1 activation in mosquitoes that fed on *P. yoelii*-infected control and TNF-α^−/−^ mice at 24 h post-blood meal, using antibody against TEP1-F (TEP1-Full) and TEP1-C (TEP1-cut). S7 was used as the internal control, and the relative ratio of TEP1-C to S7 was quantified and compared. Results are from *n* = 3 biological replicates. (**K**) *TEP1* in mosquitoes was knocked down by dsRNA interference, after which the mosquitoes were fed on control or TNF-α^−/−^ mice infected with *P. yoelii* for 4 days. Data information: Data in (**A**, **B, F**–**H**, **J**) are presented as the mean ± SD. In (**C**), the relative number of ookinetes per mosquito is represented by individual dots, with a horizontal line indicating the median, and n indicating the number of biologically independent mosquito samples. In (**D**, **K**), the number of oocysts per mosquito is represented by individual dots, with a horizontal line indicating the median, and n indicating the number of biologically independent mosquito samples. Statistical analyses for panels in (**A**, **B**,** F**–**H**, **J**) were done using unpaired *t* test (two-tailed), while the panels of (**C**, **D**, **K**) were analyzed using Mann–Whitney *U* test (two-tailed). ns no significance. Source data are available online for this figure.

To explore the underlying mechanism by which TNF-α promotes malaria transmission, we first aimed to identify the specific stage at which TNF-α exerted its effects. Real-time PCR analysis of ookinete-specific genes, including the secreted ookinete adhesive protein (*SOAP*) (Dessens et al, 2003) and von Willebrand factor A domain-related protein (*WARP*) (Raibaud et al, 2006), along with ookinete counting, revealed that TNF-α deficiency did not significantly affect the ookinete stage in the mosquito midgut (Fig. 2A–C). However, TNF-α deficiency markedly reduced oocyst numbers in mosquitoes at 2 days post blood feeding (Fig. 2D). This indicates that TNF-α facilitates malaria infection by promoting the transformation of ookinetes into early oocysts. Consequently, the carcasses of mosquitoes collected 24 h after feeding on infected control and TNF-α^−/−^ mice were subjected to mRNA sequencing and comparison.

To identify the key immune effectors in mosquitoes induced by host-derived TNF-α, we chose genes encoding antimicrobial peptides (Imler and Bulet, 2005) and peroxidase/oxidase (Kumar et al, 2010), as well as genes involved in nitration reactions (Oliveira et al, 2012) and TEP1 activation (Blandin et al, 2004)—all major immune effectors that limit malaria parasite development. We compared the expression of these genes between mosquitoes fed on the infected control and TNF-α^−/−^ mice. No significant differences were observed in the expression of genes encoding antimicrobial peptides (*Defensin*, *Gambicin*, *Cecropin-A*, and *Cecropin-B*) or peroxidase/oxidase (*DUOX*, *HPX*, *HPX15*, *DBLOX*, and *HPX8*), nor in genes related to nitration (*HPX2* and *NOX5*) between the two groups (Figs. 2E and EV3E). The mRNA levels of *Defensin* (ASTE011281) and *Cecropin-A* (ASTE007107) tended to be upregulated in hemocytes from mosquitoes fed on infected TNF-α^−/−^ mice, but their unchanged expression between two groups was determined by real-time PCR (Fig. EV3F). Additionally, the levels of reactive oxygen species (ROS), which are important immune effectors against malaria parasites (Kumar et al, 2003), were also comparable between mosquitoes fed on infected control mice and TNF-α^−/−^ mice (Fig. EV3G). Strikingly, the components of the TEP1 activation complex (Fraiture et al, 2009)—including TEP1, *Anopheles Plasmodium*-responsive leucine-rich repeat 1 (APL1), and leucine-rich repeat immune protein 1 (LRIM1)—were significantly upregulated in mosquitoes fed on infected TNF-α^−/−^ mice (Fig. 2E). The upregulation of *TEP1* (ASTE016444), *APL1* (ASTE016290), and *LRIM1* (ASTE000814) was confirmed by real-time PCR (Fig. 2F-H). The activation of TEP1, indicated by the increased ratio of TEP1-C (TEP1-cut) to S7, was further validated by Western blot analysis (Fig. 2I,J), as the 160 kDa proenzyme form of TEP1-Full is cleaved into the 100 kDa TEP1-cut upon activation (Povelones et al, 2009).

**Figure EV4:**
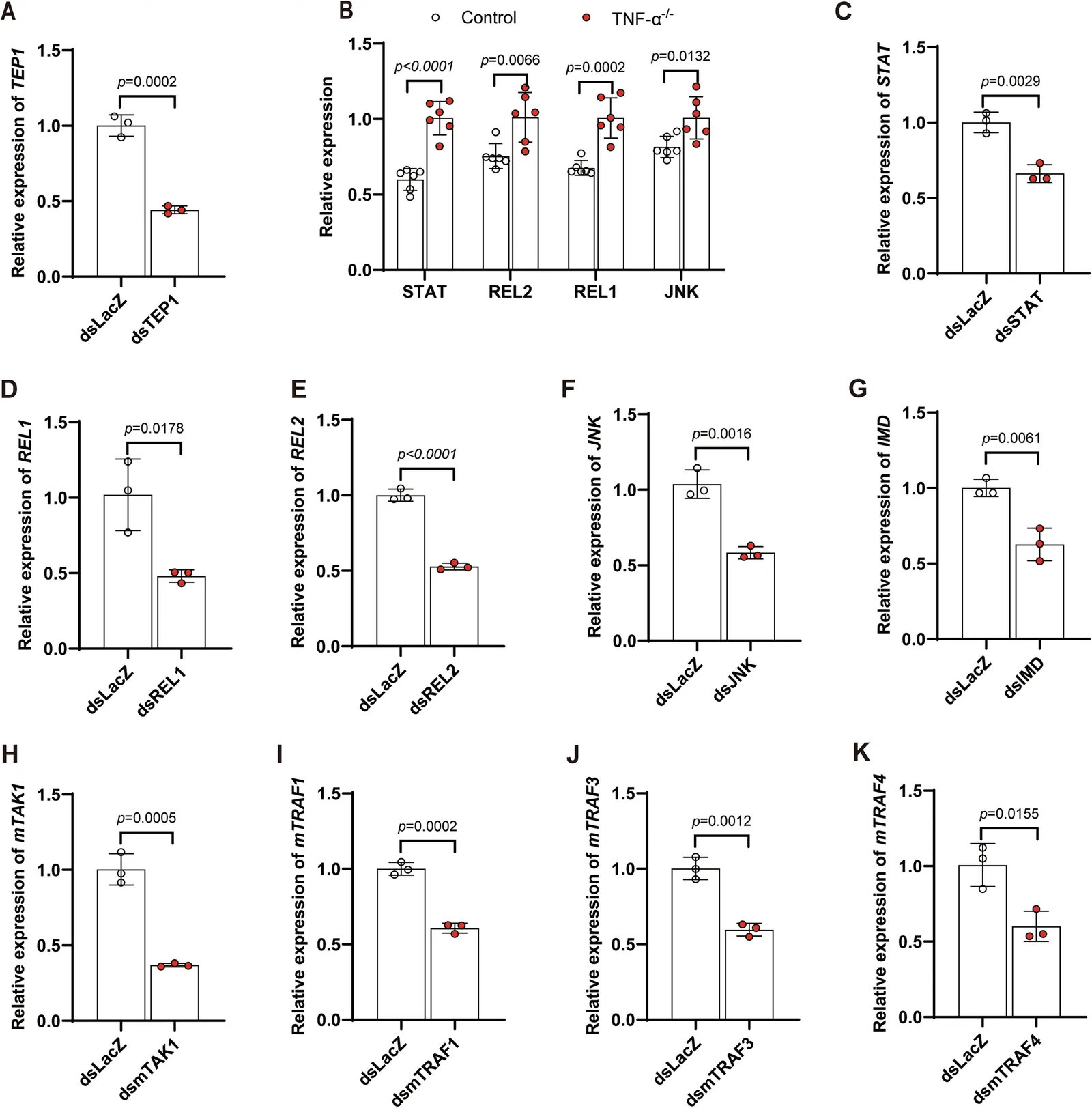
The dsRNA mediated silencing efficiency of *TEP1*, *STAT*, *REL1*, *REL2, JNK, IMD, mTAK1*, *mTRAF1*, *mTRAF3* and *mTRAF4.* (**A**, **C**–**K**) The dsRNA silencing efficiency of *TEP1* (**A**), *STAT* (**C**)*, REL1* (**D**)*, REL2* (**E**)*, JNK* (**F**), *IMD* (**G**), *mTAK1* (**H**), *mTRAF1* (**I**), *mTRAF3* (**J**) and *mTRAF4* (**K**) was assessed by qPCR. Results are from *n* = 3 biological replicates. (**B**) Relative expression of *STAT, REL2, REL1* and *JNK* between hemocytes from mosquitoes fed on *P. yoelii*-infected control and TNF-α^−/−^ mice. Results are from *n* = 6 biological replicates. Data are presented as the mean ± SD. Statistical analysis of data was conducted by unpaired *t* test (two-tailed). Source data are available online for this figure.

To determine whether TNF-α promotes malaria infection through the downregulation of TEP1, we knocked down TEP1 in mosquitoes using dsRNA interference before they fed on the infected control and TNF-α^−/−^ mice (Fig. EV4A). Consequently, the infection intensity in mosquitoes fed on the infected TNF-α^−/−^ mice was no longer reduced after TEP1 was knocked down (Fig. 2K). Taken together, our data demonstrate that TNF-α promotes malaria infection by suppressing the expression of TEP1 in mosquitoes.

### Host TNF-α-modulated TEP1 expression correlates with suppressed IMD pathway-related molecules in mosquitoes

**Figure 3:**
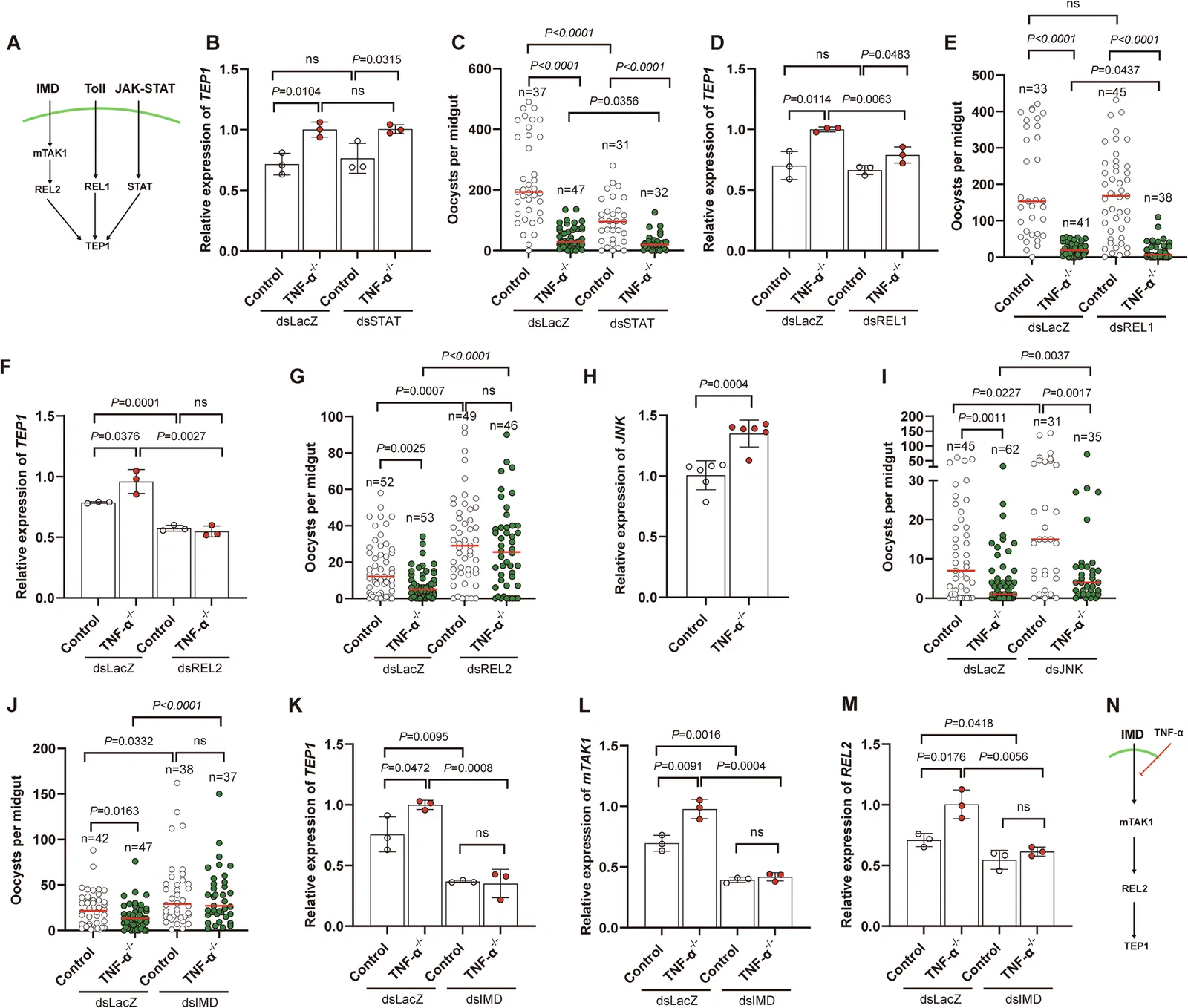
Host TNF-α-modulated TEP1 expression correlates with suppressed IMD pathway-related molecules in mosquitoes. (**A**) Schematic representation of TEP1 regulated by three immune pathways: Toll, IMD and JAK-STAT. (**B**, **C**) *STAT* in mosquitoes was knocked down, after which the mosquitoes were fed on the infected control or TNF-α^−/−^ mice. The relative expression of *TEP1* was determined by qPCR (**B**). Results are from *n* = 3 biological replicates. Oocyst number in mosquito midguts was counted at 7 days post-blood meal (**C**). (**D**, **E**) *REL1* in mosquitoes was similarly knocked down, followed by feeding on infected control or TNF-α^−/−^ mice. The relative expression of *TEP1* was measured by qPCR (**D**). Results are from *n* =  3 biological replicates. Oocyst number in mosquito midguts was quantified at 7 days post-blood meal (**E**). (**F**, **G**) *REL2* of mosquitoes was also knocked down, with subsequent feeding on infected control or TNF-α^−/−^ mice. The relative expression of *TEP1* was determined by qPCR (**F**). Results are from *n* = 3 biological replicates. Oocyst number in mosquito midguts was evaluated at 7 days post-blood meal (**G**). (**H**) Relative expression levels of *JNK* genes in mosquitoes feeding on infected control and TNF-α^−/−^ mice at 24 h post-blood meal. Results are from *n* = 6 biological replicates. (**I**) *JNK* in mosquitoes was knocked down, and mosquitoes were then fed on the infected control or TNF-α^−/−^ mice. (**J**–**M**) *IMD* in mosquitoes was knocked down, and mosquitoes were then fed on the infected control or TNF-α^−/−^ mice. Oocyst number in mosquito midguts was assessed at 7 days post-blood meal (**J**), and the relative transcriptional levels of *TEP1* (**K**), *mTAK1* (**L**), and *REL2* (**M**) were determined by qPCR. Results are from *n* = 3 biological replicates. (**N**) A schematic showed that host-derived TNF-α inhibited the activation of TEP1 by attenuating the IMD/REL2 pathway in mosquitoes. Data information: Data in panels of (**B**, **D**, **F**, **H**, **K**–**M**) are presented as the mean ± SD. In panels of (**C**, **E**, **G**, **I**, **J**), the number of oocysts per mosquito is represented by individual dots, with a horizontal line indicating the median, and n indicating the number of biologically independent mosquito samples. Statistical analysis of data in (**B**, **D**, **F**, **H**, **K**–**M**) were conducted by unpaired *t* test (two-tailed), while data in (**C**,** E**, **G**, **I**, **J**) were analyzed using Mann–Whitney *U* test (two-tailed). ns no significance. Source data are available online for this figure.

TEP1 is known to be regulated by the Toll, IMD and JAK-STAT signaling pathways (Gupta et al, 2009; Frolet et al, 2006; Meister et al, 2005) (Fig. 3A), and the upregulated expression of *STAT* (ASTE011642), *REL2* (ASTE010360) and *REL1* (ASTE011378), the essential genes from all three pathways, in hemocytes from mosquitoes fed on the infected TNF-α^−/−^ mice was demonstrated by qPCR (Fig. EV4B). To determine which pathway is involved in the modulation of *TEP1* expression by TNF-α, we assessed the effects of knocking down *STAT*, *REL2* or *REL1* on malaria parasite development and *TEP1* expression in mosquitoes. Knockdown of *STAT* (Fig. EV4C), the essential genes of JAK-STAT (Gupta et al, 2009), did not alter the differences in TEP1 expression or oocyst numbers between mosquitoes fed on infected control and TNF-α^−/−^ mice (Fig. 3B,C), although it significantly increased the oocyst number in mosquitoes fed on the infected control mice (Fig. 3C). Similarly, no significant changes of difference in *TEP1* expression or oocyst counts were observed between mosquitoes fed on infected control and TNF-α^−/−^ mice (Fig. 3D,E), after knocking down *REL*1 (Fig. EV4D), a key transcription factor in the Toll pathway (Pan et al, 2012). In contrast, knockdown of *REL2* (Fig. EV4E), the key transcription factor of the IMD pathway (Garver et al, 2012), could significantly increase the oocyst number in mosquitoes, consistent with a previous study (Garver et al, 2012). Strikingly, it could also eliminate the transcriptional differences in the immune effector *TEP1* and the oocyst number between mosquitoes fed on the infected control and TNF-α^−/−^ mice (Fig. 3F,G). Additionally, the JNK pathway is also reported to regulate TEP1 expression (Garver et al, 2013), and the key component *JNK* (ASTE007551) was also significantly upregulated in mosquitoes fed on infected TNF-α^−/−^ mice determined by real-time PCR (Fig. 3H). However, knockdown of *JNK* could not eliminate the difference in infection intensity between mosquitoes fed on infected control and TNF-α^−/−^ mice (Figs. EV4F and 3I), indicating that the JNK pathway was also not involved in this process. Collectively, these results suggest that the IMD signaling pathway may be involved in the regulation of TEP1 expression by host-derived TNF-α.

**Figure EV5:**
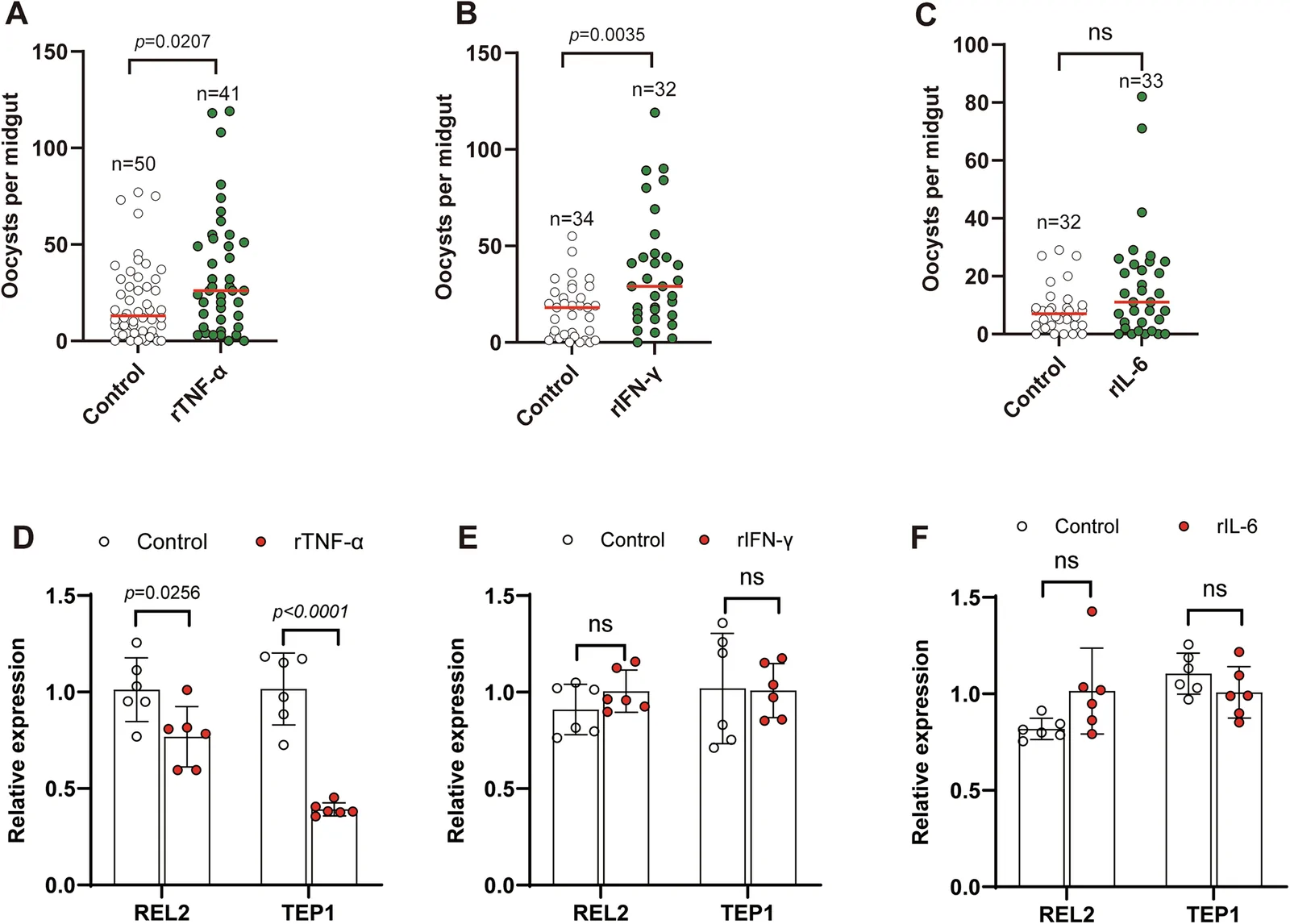
The effect of rTNF-α, rIFN-γ and rIL-6 on the parasite infection and the expression of REL2 and TEP1 in mosquitoes. (**A**–**C**) Blood was collected from mice infected with *P. yoelii* on day 4. Oocyst counts in midgut of mosquitoes fed on blood supplemented with or without rTNF-α (**A**), rIFN-γ (**B**), or rIL-6 (**C**). The number of oocysts per mosquito is represented by individual dots, with a horizontal line indicating the median, and n indicating the number of biologically independent mosquito samples. (**D**–**F**) Relative expression levels of *REL2* and *TEP1* in mosquitoes fed on blood supplemented with or without rTNF-α (**D**), rIFN-γ (**E**) or rIL-6 (**F**). Data are presented as the mean ± SD. Results are from *n* = 6 biological replicates. Data information: Statistical analysis of data in (**A**–**C**) was performed using Mann–Whitney *U* test (two-tailed), while data in (**D**–**F**) were analysed by unpaired *t* test (two-tailed). ns: no significance. Source data are available online for this figure.

To confirm the possible role of the IMD signaling pathway in this process, the *IMD* gene (ASTE008529) was also knocked down (Fig. EV4G). This knockdown abolished the differences in oocyst numbers (Fig. 3J) and the transcriptional levels of downstream molecules (Garver et al, 2012), including mosquito TAK1 (*mTAK1*, ASTE000482), *REL2*, and *TEP1*, between mosquitoes fed on infected control and TNF-α^−/−^ mice (Fig. 3K–M). Additionally, we also investigated the specific inhibitory effect of TNF-α on the IMD signaling pathway, by observing the effects of rTNF-α, rIFN-γ or rIL-6 on the IMD pathway and malaria transmission in mosquitoes using an in vitro membrane feeding assay. Supplementation with rTNF-α or rIFN-γ, but not rIL-6, significantly increased the parasite infection intensity in mosquitoes (Fig. EV5A–C). However, only rTNF-α, but not rIFN-γ, could significantly downregulate the expression of both *REL2* and *TEP1* (Fig. EV5D–F). Thus, our data demonstrate that host TNF-α-regulated TEP1 expression is linked to the downregulation of mosquito IMD-associated immune markers (Fig. 3N).

### Host-derived TNF-α suppresses IMD pathway-related molecules through the *mTRAF3*-mediated inhibition of mTAK1

**Figure 4:**
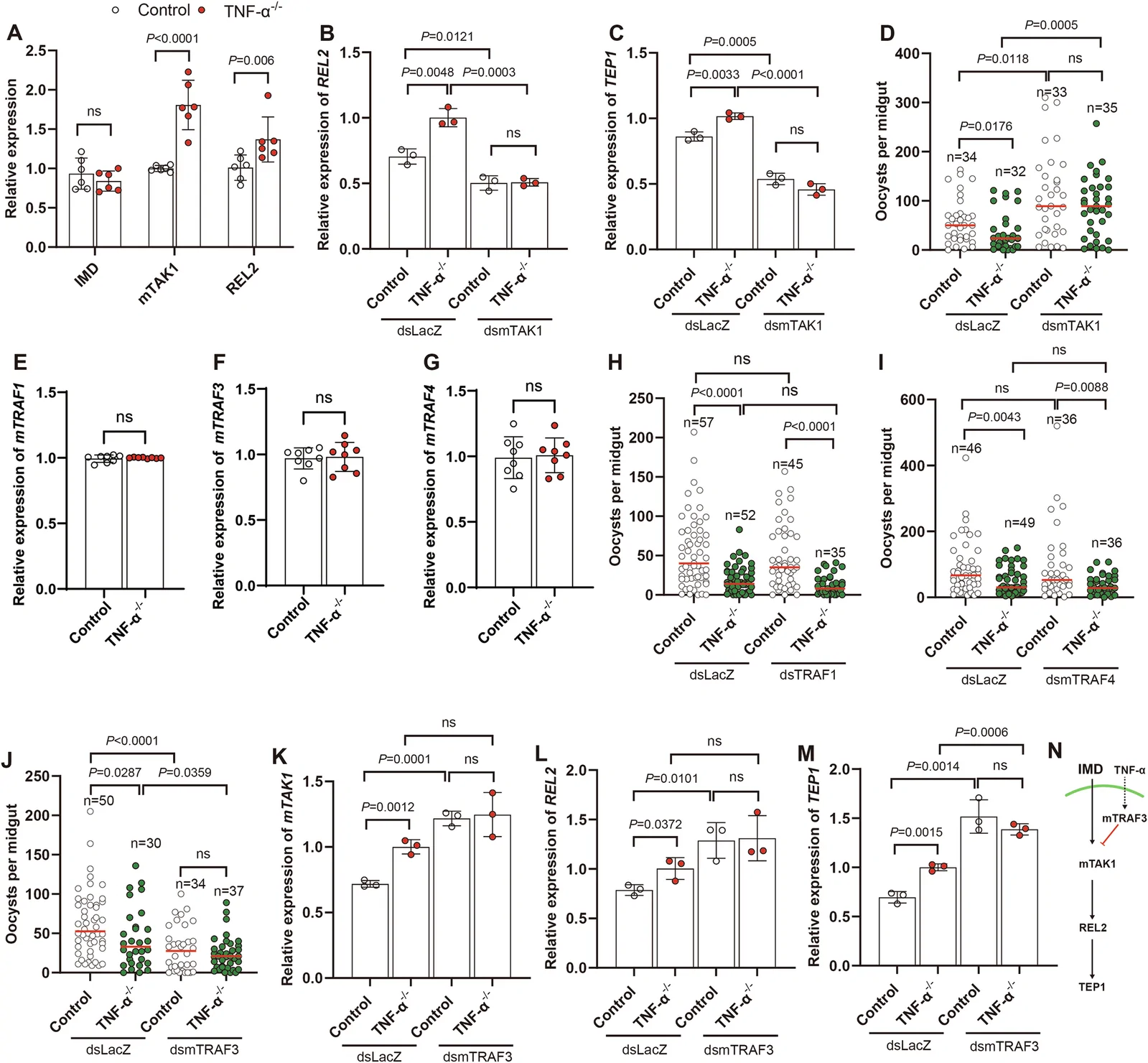
Host-derived TNF-α suppresses IMD pathway-related molecules through *mTRAF3*-mediated inhibition of mTAK1. (**A**) Relative expression levels of *IMD*, *mTAK1* and *REL2* in mosquitoes fed on infected control or TNF-α^−/−^ mice were determined by qPCR at 24 h post-blood meal. Results are from *n* = 6 biological replicates. (**B**–**D**) *mTAK1* in mosquitoes was knocked down, followed by feeding on infected control or TNF-α^−/−^ mice. Relative expression levels of *REL2* (**B**) and *TEP1* (**C**) in mosquitoes were determined by qPCR at 24 h post-blood feeding. Results are from *n* = 3 biological replicates. Oocyst number in mosquito midguts was counted at 7 days post-blood meal (**D**). (**E**–**G**) Relative mRNA levels of *mTRAF1* (**E**), *mTRAF3* (**F**) and *mTRAF4* (**G**) in mosquitoes fed on infected control or TNF-α^−/−^ mice were determined by qPCR at 24 h post-blood meal. Results are from *n* = 8 biological replicates. (**H**, **I**) *mTRAF1* (**H**) or *mTRAF4* (**I**) of mosquitoes was knocked down, and mosquitoes were then fed on infected control or TNF-α^−/−^ mice. Oocyst number in mosquito midguts was quantified at 7 days post-blood meal. (**J**–**M**) *mTRAF3* in mosquitoes was knocked down, and mosquitoes were then fed on infected control or TNF-α^−/−^ mice. Oocyst number in mosquito midguts was counted at 7 days post-blood meal (**J**), and the relative expression levels of *mTAK1* (**K**), *REL2* (**L**) and *TEP1* (**M**) in mosquitoes were determined by qPCR at 24 h post-blood feeding. Results are from *n* = 3 biological replicates. (**N**) A schematic showed that TNF-α/Wgn signaling acts through mTRAF3 and inhibited IMD pathway by interfering *mTAK1* expression. Data information: Data in panels (**A**–**C**,** E**–**G**,** K**–**M**) are presented as the mean ± SD. In panels (**D**, **H**–**J**), the number of oocysts per mosquito is represented by individual dots, with a horizontal line indicating the median, and n indicating the number of biologically independent mosquito samples. Statistical analysis of data in (**A**–**C**, **E**–**G**, **K**–**M**) was done by unpaired *t* test (two-tailed), while the data in (**D**, **H**–**J**) were analyzed by using Mann–Whitney *U* test (two-tailed). ns no significance. Source data are available online for this figure.

Next, we aimed to determine at which point the IMD pathway was inhibited by host-derived TNF-α. Although the transcription of downstream molecules such as *mTAK1* and *REL2* was upregulated, real-time PCR analysis revealed no significant change in the upstream molecule IMD between mosquitoes fed on infected control and TNF-α^−/−^ mice (Fig. 4A). This suggests that host-derived TNF-α interferes with the IMD pathway at the level of *mTAK1*. Knockdown of *mTAK1* significantly reduced the transcription levels of *REL2* and *TEP1* (Figs. EV4H and 4B,C), while increasing the oocyst numbers in mosquitoes fed on infected TNF-α^−/−^ mice (Fig. 4D). These findings support the essential role of mTAK1 in TNF-α-mediated promotion of malaria transmission.

Studies on *Drosophila* have shown that the expression of *TAK1* is negatively regulated by TNF receptor-associated factor (*TRAF*) 2 and *TRAF3* (Loudhaief et al, 2023). Consequently, we identified several orthologs of TRAFs in mosquitoes (mTRAFs), while the mRNA levels of *mTRAF1* (ASTE011577), *mTRAF3* (ASTE008048), and *mTRAF4* (ASTE007686) were found to be comparable between mosquitoes fed on infected control and TNF-α^−/−^ mice (Fig. 4E–G). To identify which *mTRAF* is involved in the regulation of *mTAK1*, we evaluated the effects of knocking down *mTRAF1*, *mTRAF3*, or *mTRAF4* on the transcription levels of *mTAK1* and parasite infection in mosquitoes. Only the knockdown of *mTRAF3*, but not *mTRAF1* and *mTRAF4*, was found to abolish the difference in infection intensity between mosquitoes fed on the infected control and TNF-α^−/−^ mice (Figs. EV4I–K and 4H–J). Furthermore, the different mRNA levels of *mTAK1, REL2* and *TEP1* between mosquitoes fed on the infected control and TNF-α^−/−^ mice disappeared after *mTRAF3* was knocked down (Fig. 4K–M). Taken together, our data show that host-derived TNF-α suppresses IMD pathway-related molecules through the *mTRAF3*-mediated inhibition of mTAK1. (Fig. 4N).

### Host-derived TNF-α preferentially binds to the mosquito TNF receptor Wgn to dampen IMD pathway-related immune markers

**Figure EV6:**
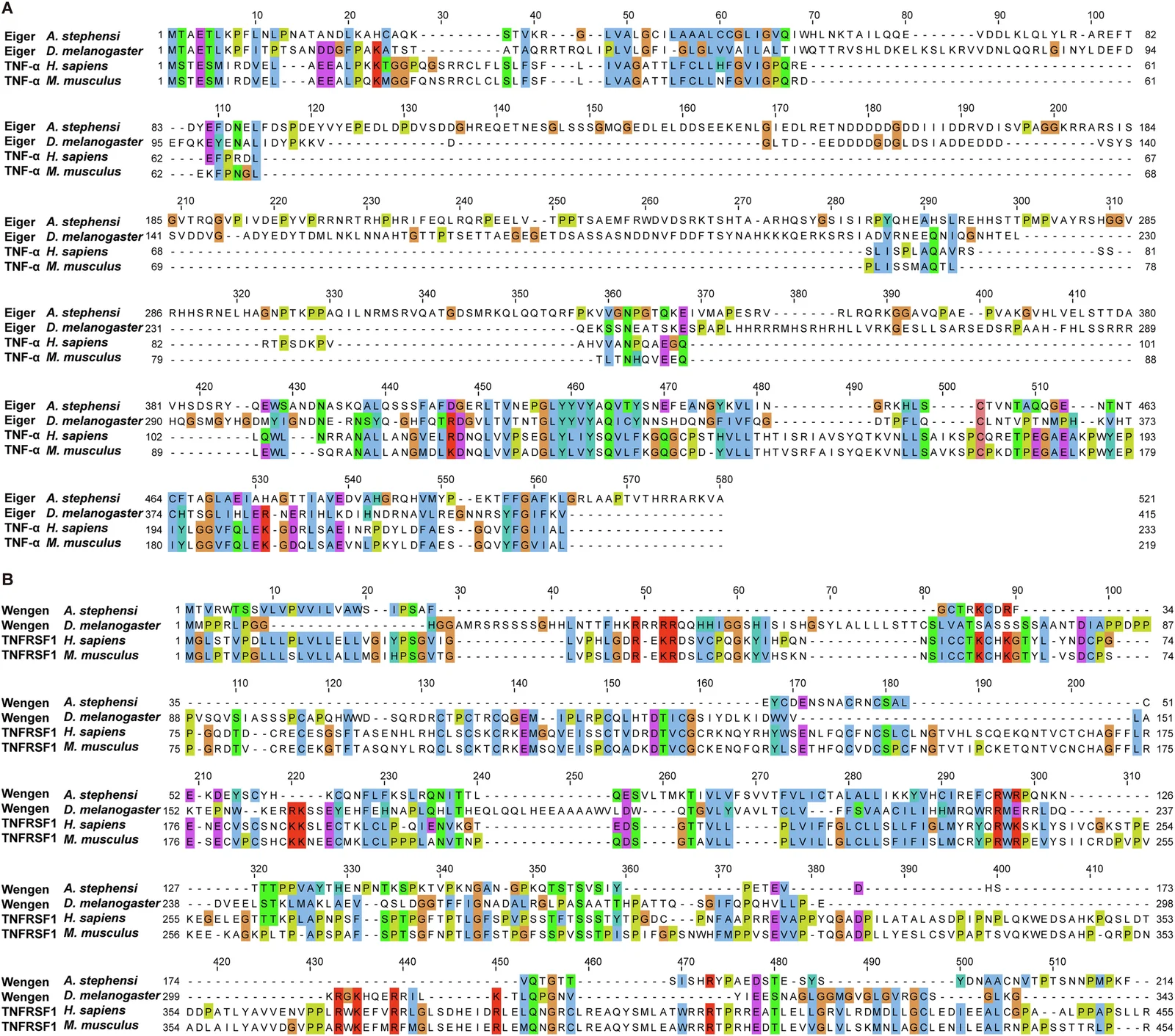
Sequence alignment of Eiger and Wgn with their homologs in mammalians. (**A**) Sequence alignment of the Eiger from *An. stephensi* and *D. melanogaster* with TNF-α from *M. musculus* and *H. sapiens*. (**B**) Sequence alignment of the Wgn from *An. stephensi* and *D. melanogaster* with TNFRs from *M. musculus* and *H. sapiens*. Different colors indicate varying levels of homology among the alignments. Source data are available online for this figure.

Host TNF-α has a homolog in *Drosophila* known as Eiger, which interacts with two homologous TNF-α receptors: Wgn (Kauppila et al, 2003) and Grnd (Andersen et al, 2015). Eiger binds to these two receptors with differing affinities, regulating distinct cellular functions (Palmerini et al, 2021). Further studies have shown that Eiger’s binding to Wgn can modulate lipid catabolism and immune activity in *Drosophila* through the recruitment of *TRAF2* and 3, respectively (Loudhaief et al, 2023). Considering the similarity of both Eiger and Wgn in *Drosophila* and mosquitoes (Fig. EV6A,B), we proposed that host-derived TNF-α preferentially bound to Wgn in mosquitoes. This interaction might activate mTRAF3, which subsequently inhibited IMD-related immune markers by downregulating *mTAK1* expression.

**Figure EV7:**
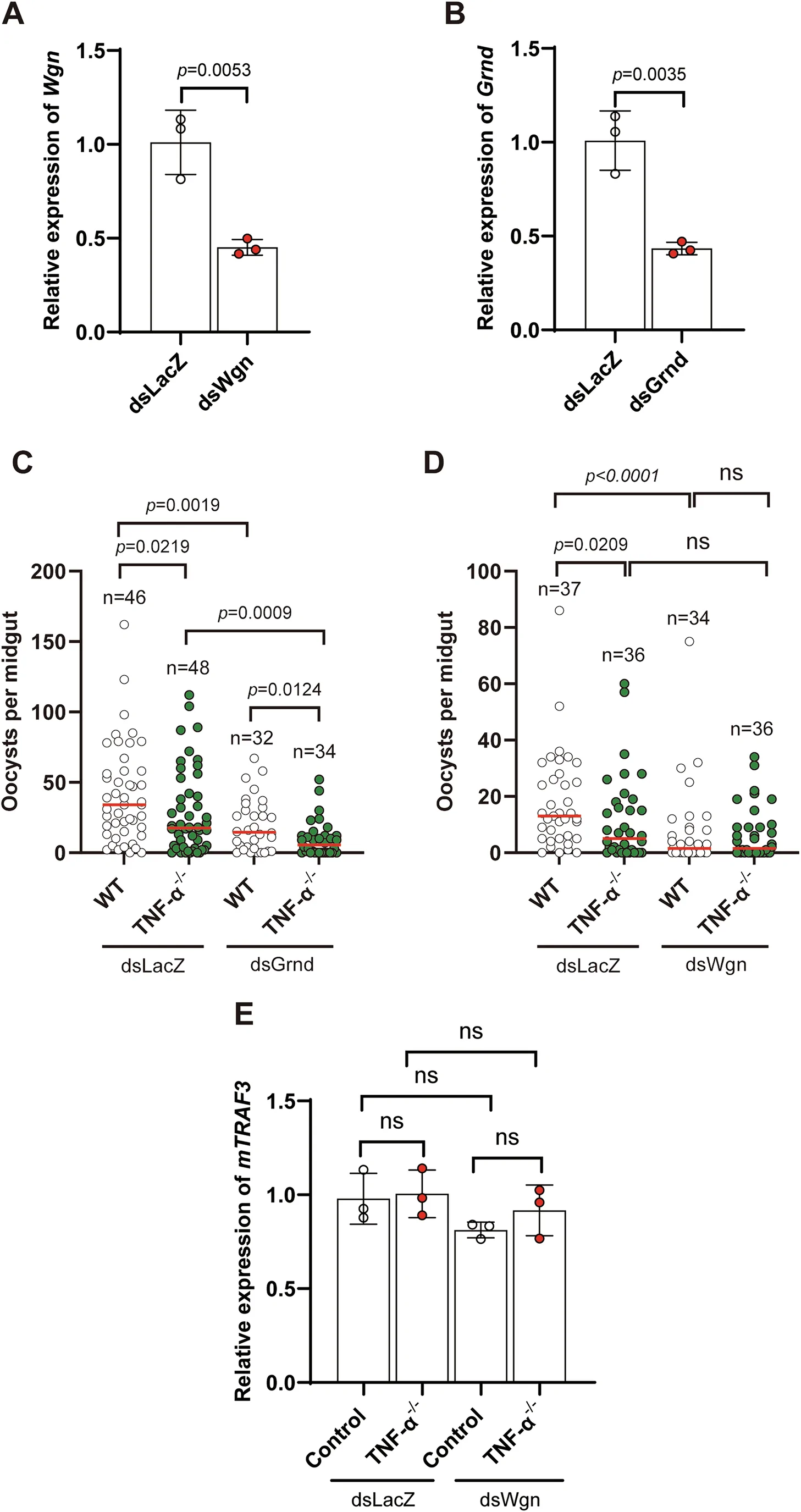
The effect of knockdown of *Wgn* or *Grnd* on *P. berghei* infection in mosquitoes. (**A**, **B**) The dsRNA silencing efficiency of *Wgn* (**A**) and *Grnd* (**B**) was determined by qPCR. Results are from *n* = 3 biological replicates. (**C**, **D**) *Grnd* (**C**) or *Wgn* (**D**) of mosquitoes was knocked down, and mosquitoes were then fed on *P. berghei*-infected control or TNF-α^−/−^ mice. Oocyst number in mosquito midguts was counted at 7 days post-blood meal. The number of oocysts per mosquito is represented by individual dots, with a horizontal line indicating the median, and n indicating the number of biologically independent mosquito samples. (**E**) The relative expression levels of *mTRAF3* in mosquitoes with *Wgn* knocked down were evaluated by qPCR at 24 h post-blood feeding. Results are from *n* =  3 biological replicates. Data information: Data in (**A**, **B**, **E**) are presented as the mean ± SD. Statistical analysis of data in (**A**, **B**, **E**) was done by unpaired *t* test (two-tailed), while data in (**C**, **D**) was analysed using a Mann–Whitney *U* test (two-tailed). ns no significance. Source data are available online for this figure.

**Figure 5:**
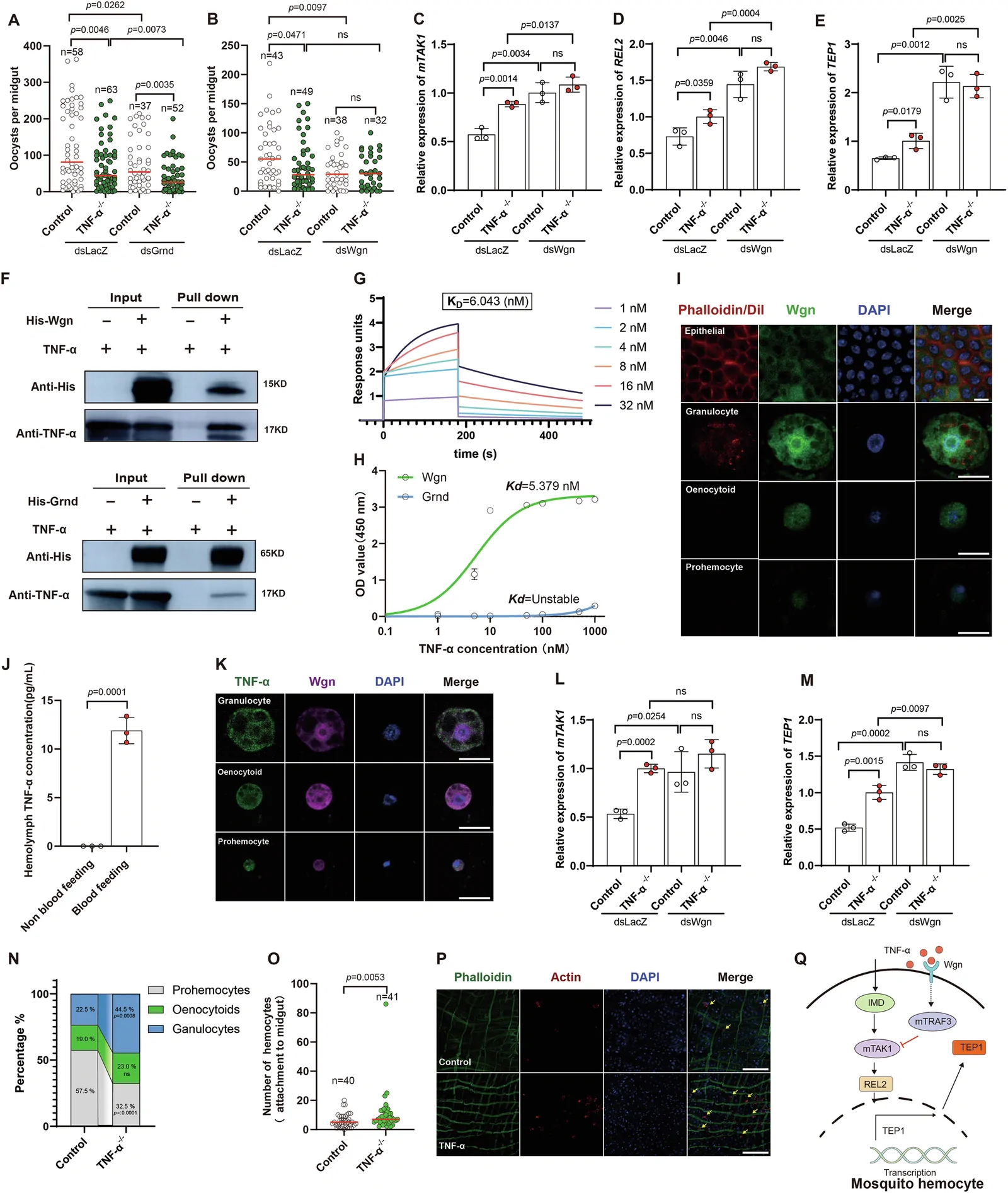
Host-derived TNF-α preferentially binds to the mosquito TNF receptor Wgn to dampen IMD pathway-related immune markers. (**A**, **B**) *Grnd* (**A**) or *Wgn* (**B**) of mosquitoes was knocked down, and mosquitoes were then fed on infected control or TNF-α^−/−^ mice. Oocyst number in mosquito midguts was counted at 7 days post-blood meal. (**C**–**E**) The relative expression levels of *mTAK1* (**C**), *REL2* (**D**) and *TEP1* (**E**) in mosquitoes with *Wgn* knockdown were evaluated by qPCR at 24 h post-blood meal. Results are from *n* = 3 biological replicates. (**F**) The pull-down assay shows the interaction between TNF-α and His-Wgn as well as His-Grnd, which were attached to anti-His beads. (**G**) Binding affinity of Wgn and Grnd for recombinant TNF-α was measured by SPR. Sensorgrams show the response upon injection of various concentrations of Wgn over a TNF-α-immobilized surface. Data were fitted to a 1:1 binding model (local fit), revealing a high-affinity interaction with an equilibrium dissociation constant (K_D_) of 6.043 nM. No binding signal was observed between Grnd and rTNF-α. (**H**) Representative binding curve of Wgn and rTNF-α determined by ELISA. Absorbance at 450 nm was plotted against the concentration of TNF-α, data points represent the mean ± SD of three technical replicates. The curve was fitted by non-linear regression using a one-site specific binding model (Langmuir isotherm). No binding signal was detected for Grnd with rTNF-α. (**I**) Immunofluorescence assay showed the distribution of Wgn in midgut epithelial cells and hemocytes from mosquitoes fed on the infected mice. Mosquitoes were intrathoracically injected with Dil and then allowed to feed on *P. yoelii*-infected mice. Midguts were dissected and stained with anti-Wgn (green), Phalloidin (red), and DAPI (blue). Hemocytes were perfused and then stained with anti-Wgn (green) and DAPI (blue), and subsets—granulocytes, oenocytoids, and prohemocytes—were identified based on morphology, with granulocytes further confirmed by Dil (red) signal. Scale bar: 10 µm. (**J**) Hemolymph was collected from non-blood-fed mosquitoes or 24 h post-blood meal mosquito. The concentration of TNF-α in mosquito hemolymph was detected by ELISA. Results are from *n* = 3 biological replicates. (**K**) Immunofluorescence assay showed the binding of host TNF-α (green) to Wgn (purple) on granulocytes, oenocytoids and prohemocytes. Scale bar: 10 µm. (**L**, **M**) *Wgn* in mosquitoes was knocked down, and mosquitoes were then fed on the infected control or TNF-α^−/−^ mice. Hemocytes from mosquitoes were collected and the relative mRNA levels of *mTAK1* (**L**) and *TEP1* (**M**) were evaluated by qPCR at 24 h post-blood meal. Results are from *n* = 3 biological replicates. (**N**) The percentage of granulocytes, oenocytoids and prohemocytes in mosquitoes that fed on the infected control or TNF-α^−/−^ mice was compared. (**O**, **P**) The number of hemocytes attached to the midgut in mosquitoes fed on the infected control and TNF-α^−/−^ mice was statistically analyzed (**O**), representative immunofluorescence image of hemocytes attached to the midgut is shown (**P**). Scale bar: 25 µm. (**Q**) A schematic showing that TNF-α inhibits the IMD pathway by preferentially binding to mosquito TNF-α receptor Wgn. Data information: The individual dots in panels of (**A**, **B**) represent the number of oocysts per mosquito, with a line indicating the median, and n indicating the number of biologically independent mosquito samples. Data in (**C**–**E**, **J**,** L**, **M**) are presented as the mean ± SD. Data in panels of (**O**) represent the number of hemocytes per midgut, with a line indicating the median, and n indicating the number of biologically independent mosquito samples. Statistical analysis of data in (**A**, **B**, **O**) were performed by Mann–Whitney *U* test (two-tailed), while data in (**C**–**E**, **J**, **L**, **M**) were analyzed by unpaired *t* test (two-tailed). Data in (**N**) was analyzed by Fisher’s exact test (two-sided). ns no significance. Source data are available online for this figure.

To test this hypothesis, either *Wgn* or *Grnd* was knocked down before feeding mosquitoes on the infected control and TNF-α^−/−^ mice. Knockdown of either *Wgn* (ASTE010852) or *Grnd* (ASTE008719) significantly reduced infection intensity in mosquitoes fed on either *P. yoelii*- or *P. berghei*-infected mice (Figs. EV7A–D and 5A,B), suggesting their intrinsic roles in suppressing mosquito immune responses. Notably, only the knockdown of *Wgn* eliminated the differences in infection intensity between mosquitoes fed on control and TNF-α^−/−^ mice (Fig. 5A,B), as well as the transcriptional differences of *mTRAF3*, *mTAK1*, *REL2*, and *TEP1* (Figs. EV7E and 5C–E). Pull-down assays revealed that host-derived TNF-α preferentially binds to Wgn, but not to Grnd (Fig. 5F). Moreover, both surface plasmon resonance (SPR) and ELISA demonstrated that TNF-α exhibited a significantly higher affinity for Wgn than for Grnd (Fig. 5G,H), confirming that host TNF-α specifically targeted Wgn.

Wgn was detected in both midgut epithelial cells and hemocytes (Fig. 5I), with a prominent enrichment in granulocytes and oenocytoids among the hemocytes (Fig. 5I). Furthermore, host-derived TNF-α was detectable in mosquito hemolymph at 24 h post-blood meal (Fig. 5J), indicating that host-derived TNF-α could enter the mosquito midgut and successfully reach the hemolymph. Given that TEP1 is predominantly expressed in hemocytes, we investigated whether host-derived TNF-α directly regulated TEP1 expression in hemocytes through Wgn. Immunofluorescence assay revealed that host TNF-α bound to Wgn on all three hemocyte subsets (Fig. 5K). Both *mTAK1* and *TEP1* were upregulated in hemocytes of mosquitoes fed on the infected TNF-α^−/−^ mice (Fig. 5L,M). However, after knocking down *Wgn*, the expression levels of these genes were comparable between mosquitoes fed on the infected control and TNF-α^−/−^ mice (Fig. 5L,M), suggesting that host-derived TNF-α might suppress the IMD signaling pathway in hemocytes to limit TEP1 production. Notably, we also observed a significantly increased proportion of granulocytes and oenocytoids, accompanied by a decreased proportion of prohemocytes, in the hemolymph of mosquitoes fed on the infected TNFα^−/−^ mice (Fig. 5N). Furthermore, increased hemocyte attachment to the midgut basal lamina was observed in mosquitoes fed on the infected TNF-α^−/−^ mice compared with those fed on the infected control mice (Fig. 5O,P). Together, these results suggest that host-derived TNF-α may impede the differentiation of prohemocytes into granulocytes and oenocytoids (Fig. 5Q).

### Host-derived TNF-α promotes human malaria infection in mosquitoes in a cross-species manner

**Figure 6:**
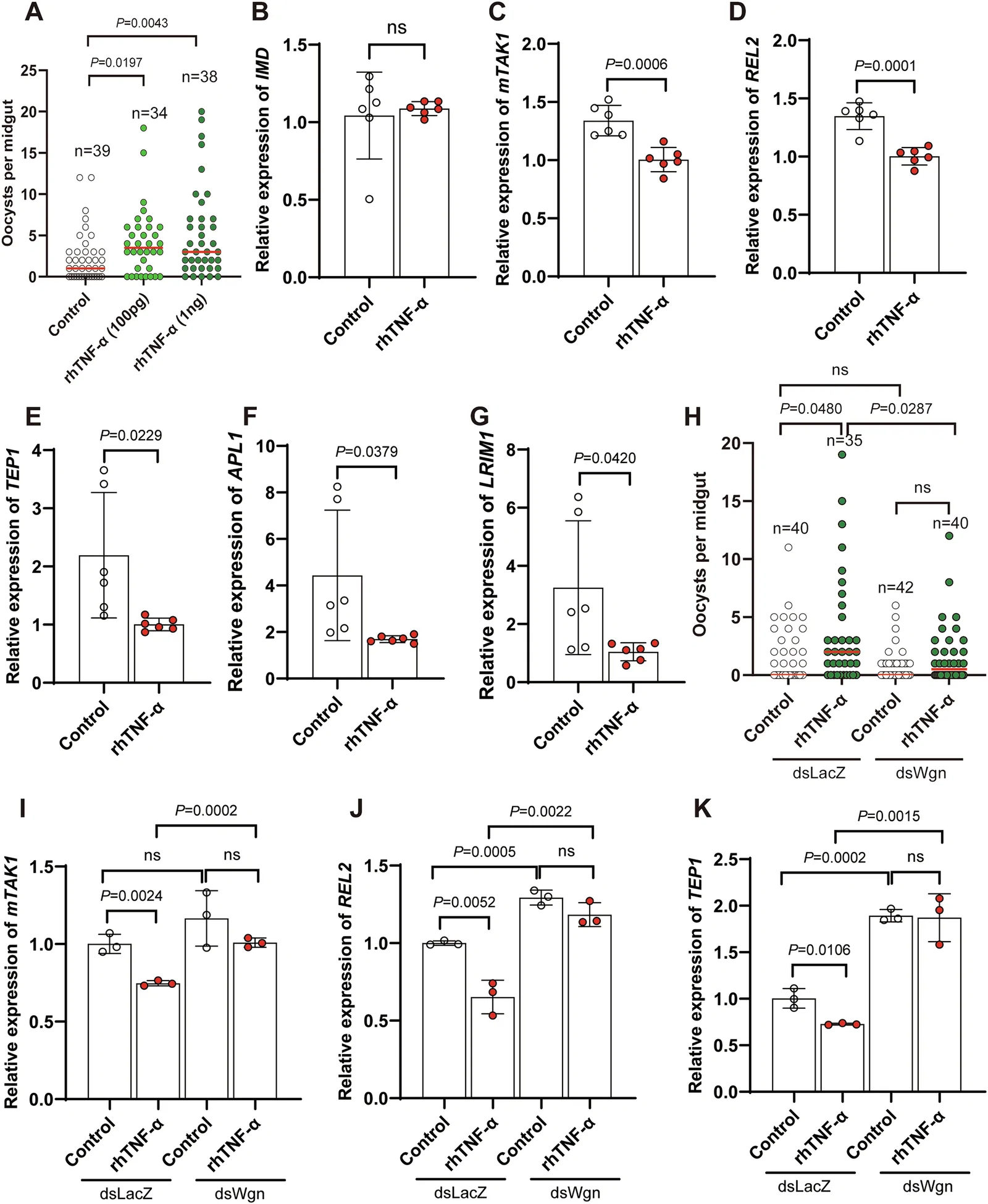
Host-derived TNF-α promotes human malaria infection in mosquitoes in a cross-species manner. (**A**) Oocyst counts in mosquitoes fed on *P. falciparum* gametocytes supplemented with or without 100 pg/mL or 1 ng/mL of rhTNF-α at 9 days post-blood meal. (**B**–**G**) Relative mRNA levels of *IMD* (**B**), *mTAK1* (**C**), *REL2* (**D**), *TEP1* (**E**), *APL1* (**F**) and *LRIM1* (**G**) were determined by qPCR in mosquitoes fed on *P. falciparum* gametocyte in the presence of rhTNF-α. Results are from *n* = 6 biological replicates. (**H**–**K**) *Wgn* in mosquitoes was knocked down, and mosquitoes were then fed on *P. falciparum* gametocytes supplemented with rhTNF-α. 24 h later, the relative mRNA levels of *mTAK1* (**I**), *REL2* (**J**) and *TEP1* (**K**) in mosquitoes were determined by qPCR. Results are from *n* = 3 biological replicates. Oocyst number in mosquito was counted at 9 days post blood meal (**H**). Data information: Data in (**B**–**G**, **I**–**K**) are presented as the mean ± SD. In (**A**, **H**), the individual dots represent the number of oocysts per mosquito, with a line indicating the median, and n indicating the number of biologically independent mosquito samples. Statistical analysis of data in (**A**, **H**) were performed by Mann–Whitney *U* test (two-tailed), while data in (**B**–**G**, **I**–**K**) was analyzed by unpaired *t* test (two-tailed). ns no significance. Source data are available online for this figure.

To investigate whether host-derived TNF-α could enhance human malaria infection, various doses of recombinant human TNF-α (rhTNF-α) were added to in vitro cultured *P. falciparum* gametocytes before being fed to mosquitoes using the standard membrane feeding assay (SMFA). The addition of rhTNF-α, which was at physiological concentrations (100 pg/ml or 1 ng/ml) (Duarte et al, 2007), significantly increased the infection intensity in mosquitoes (Fig. 6A). Although the transcriptional level of the *IMD* gene remained unchanged (Fig. 6B), other IMD pathway-related genes, including *REL2* and *mTAK1*, as well as *TEP1*, *APL1*, and *LRIM1*, were significantly downregulated in mosquitoes after the addition of rhTNF-α to the cultured *P. falciparum* gametocytes (Fig. 6C–G). Importantly, the knockdown of *Wgn* reversed the promoting effect of rhTNF-α on *P. falciparum* infection in mosquitoes (Fig. 6H) and restored the transcriptional level of *mTAK1*, *REL2* and *TEP1* (Fig. 6I–K). Therefore, we demonstrate that host-derived TNF-α can promote human malaria infection in mosquitoes in a cross-species manner.

### Host-derived TNF-α reduces the transmission-blocking efficacy of anti-Pfs25

**Figure 7:**
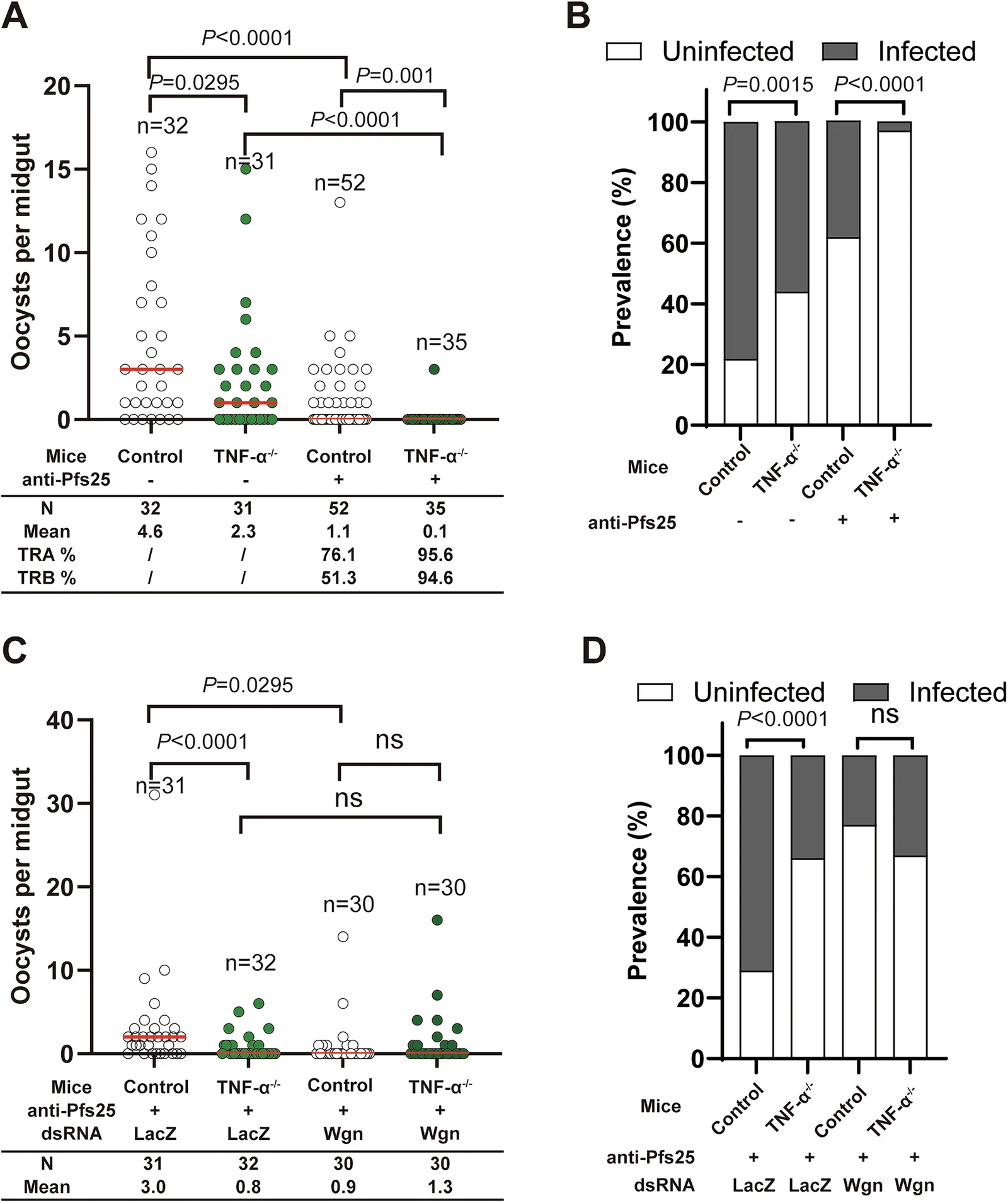
Host-derived TNF-α reduces the transmission blocking efficacy of anti-Pfs25. (**A**, **B**) Oocyst number (**A**) and prevalence (**B**) in mosquitoes fed on *P. b-pfs25*-infected control or TNF-α^−/−^ mice pre-treated with or without anti-Pfs25 monoclonal antibody. (**C**, **D**) *Wgn* in mosquitoes was knocked down, and mosquitoes were then fed on *P. b-pfs25*-infected control or TNF-α^−/−^ mice pre-treated with or without anti-Pfs25 monoclonal antibody. The oocyst number (**C**) and prevalence (**D**) of mosquitoes were quantified at 9 days post blood meal. Data information: The individual dots in (**A**, **C**) represent the oocyst number per mosquito, with a line indicating the median, and n indicating the number of biologically independent mosquito samples. Statistical analysis of data in (**A**, **C**) was performed by Mann–Whitney *U* test (two-tailed), while data in (**B**, **D**) was analyzed by Fisher’s exact test (two-sided). ns no significance. Source data are available online for this figure.

Malaria transmission-blocking vaccines (TBVs) are designed to elicit antibodies against surface proteins on the sexual stages of *Plasmodium* parasites, such as pfs230, pfs48/45, and pfs25 (Acquah et al, 2019). The efficacy of TBVs in blocking transmission relies on the production of antibodies targeting these sexual stages, and their effectiveness is typically assessed using SMFA. However, SMFA does not account for host-derived factors, leaving it unclear whether these factors might influence the efficacy of TBVs. To address this, *Pfs25* transgenic *P. berghei* (*P. b-pfs*25) was utilized to assess the impact of host-derived TNF-α on the transmission-blocking efficacy of anti-Pfs25. Control and TNF-α^−/−^ mice infected with *P. b-pfs25* were intravenously injected with anti-Pfs25 antibodies and subsequently fed to mosquitoes. Both transmission-reducing activity (TRA) and transmission-blocking activity (TBA) of anti-pfs25 in mosquitoes fed on infected control mice were substantially lower compared to mosquitoes fed on TNF-α^−/−^ infected mice (Fig. 7A,B). Interestingly, the influence of TNF-α on both TRA and TBA against *P. b-pfs25* was completely abolished when the *Wgn* gene in mosquitoes was knocked down (Fig. 7C,D). These findings strongly suggest that host-derived TNF-α negatively affects the transmission-blocking efficacy of anti-Pfs25 by acting through Wgn.

## Discussion

The role of host-derived factors in modulating malaria transmission remains largely unexplored. To our surprise, we discovered that host-derived TNF-α significantly promotes, rather than reduces, both rodent and human malaria infection in mosquitoes. Mechanistically, host-derived TNF-α preferentially binds to Wgn and acts through the downstream *mTRAF3*, which inhibits the IMD-related immune molecules and reduces the expression of TEP1. Furthermore, neutralizing host-derived TNF-α not only prevents ECM development, but also enhances the transmission-blocking efficacy of anti-Pfs25 antibody. These findings reveal a novel strategy by which host-derived TNF-α can be utilized by the parasite to facilitate transmission in mosquitoes. Our study offers a potential new approach to prevent ECM and reduce malaria transmission by targeting TNF-α.

Previous studies have established that various host-derived factors can significantly influence malaria transmission. These include antibodies targeting sexual stages (Stone et al, 2016), complement components (e.g., C3 and C5) (Tsuboi et al, 1995; He et al, 2025), human chitotriosidase (Di Luca et al, 2007), and elements of the fibrinolytic system (Alves E Silva et al, 2021). These factors can act through direct mechanisms, such as targeting parasites to block their development (Stone et al, 2016), or indirectly by modulating processes like the formation of the mosquito peritrophic matrix or altering the abundance of antimalarial bacteria within the vector (He et al, 2025). Furthermore, host molecules, such as insulin and TGF-β1, when ingested during a blood meal, have been reported to modulate malaria transmission by activating specific mosquito immune responses. For instance, ingested human insulin was found to elevate *P. falciparum* burden in *An. stephensi* via regulation of NF-κB and suppression of antimicrobial peptides (Pakpour et al, 2012). Similarly, ingested TGF-β1 exhibits a concentration-dependent effect on rodent malaria parasite development in mosquitoes (Luckhart et al, 2003; Lieber & Luckhart, 2004). In contrast, our study reveals that host-derived TNF-α can suppress antimalarial immune responses of mosquitoes in a cross-species manner, ultimately facilitating malaria transmission. This finding further underscores the remarkable complexity within the tripartite host-vector-parasite interaction network of malaria.

Notably, a previous study demonstrated that host-derived TNF-α impaired zygote development in vitro (Ramiro et al, 2011), suggesting a negative impact on malaria transmission, which appeared to contradict our findings. This discrepancy might arise from the fact that the inhibitory effect of TNF-α on malaria transmission was observed in an in vitro setting, while our study was conducted in vivo. Additionally, a recent study reported that TNF-α could inhibit malaria infection in mosquitoes (Stone et al, 2016). However, this study used a high dose of rhTNF-α (50 ng) (Samantsidis et al, 2025), which was intrathoracically injected into mosquitoes—a scenario that does not occur naturally during malaria transmission across species. In addition, *P. berghei* was used in previous studies, while our study utilized *P. yoelii*. We confirmed that silencing of either *Wgn* or *Grnd* resulted in significantly reduced *P. berghei* infection in mosquitoes, which is also contradictory to what is found in the previous study. Therefore, the discrepancy between our and previous study was not due to the different species used. We proposed that this discrepancy might be interpreted by the different dose used in our and previous study. We demonstrated that TNF-α at physiological concentrations (100 pg/mL–1 ng/mL) has a significantly higher affinity for binding with Wgn compared to Grnd. However, at sufficiently high levels, TNF-α might activate both the high-affinity receptor Wgn and the low-affinity receptor Grnd. Since the activation of Wgn and Grnd by Eiger in *Drosophila* is known to promote distinct cellular functions (Palmerini et al, 2021), it is plausible that high concentrations of TNF-α can elicit responses that contradict those induced by lower concentrations. This suggests a dual effect of dose-dependent TNF-α on the mosquito immune response. Interestingly, a similar dual effect has been reported regarding TNF-α‘s influence on the growth of mammalian cells (Tang et al, 2013). Additionally, TNF-α has broad influences on inflammatory (van Loo and Bertrand, 2023) and metabolic (Sethi and Hotamisligil, 2021), and its depletion may influence insulin signaling (Panda et al, 2025) and produce other host blood factors in infected mice. Thus, we cannot fully rule out indirect impacts of TNF-α deficiency on malaria transmission, even though feeding mosquitoes on infected mice pre-treated with anti-TNFR1 and anti-TNFR2 antibodies yielded no significant differences in parasite transmission.

We found host-derived TNF-α inhibited mosquito IMD pathway-related immune markers in a mTRAF3-dependent manner, while dTRAF2 was essential for the regulation of immune response in *Drosophila*. This may reflect variations in the downstream signaling molecules utilized by Wgn in the regulation of immune responses in *Drosophila* compared to mosquitoes. As dTRAF3 was involved in the limitation of autophagy-dependent lipolysis in *Drosophila* (Loudhaief et al, 2023), the effect of mTRAF3 on the regulation of mosquito lipid metabolism could not be completely excluded. Although we found Grnd was not involved in the TNF-α-promoted malaria transmission, knockdown of Grnd also significantly increased the parasite infection in mosquitoes, indicating the important role of Grnd in the modulation of mosquito immune responses. As *Drosophila* Eiger was demonstrated to have a significantly higher affinity to bind to Grnd than Wgn (Palmerini et al, 2021), Grnd signaling in mosquitoes might be triggered by Eiger.

It is well established that *An. gambiae* exhibits isoform-specific roles of APL1A versus APL1C in defending against rodent versus human malaria parasites, respectively. However, unlike *An. gambiae*, which carries three *APL1* isoforms, only a single *APL1* ortholog has been identified in *An. stephensi* (Mitri et al, 2020). The role of the IMD pathway in *An. gambiae* responses to rodent malaria infection appears contradictory (Garver et al, 2009; Meister et al, 2005). In contrast, we found the essential role of the IMD pathway in *An. stephensi* against rodent malaria infection, which is consistent with a prior study (Feng et al, 2021). Additionally, structural similarity-based homology searches revealed that *An. stephensi* encodes only one *STAT* gene, unlike *An. gambiae*, which carries two paralogs designated *STAT*-A and *STAT*-B. Furthermore, silencing *STAT* in *An. stephensi* yielded infection phenotypes distinct from those observed following *STAT*-A knockdown in *An. gambiae* (Gupta et al, 2009). These indicate that the immune responses of *An. stephensi* against malaria parasites differ from those of *An. gambiae*, and results should be interpreted with caution across different parasite-mosquito combinations.

TBVs are considered a promising strategy for controlling and potentially eliminating malaria worldwide. Despite significant efforts to optimize TBV design and induce high levels of antibodies against malaria sexual stages, no TBVs have been licensed to date. Interestingly, we discover that host-derived TNF-α can significantly reduce the blocking efficacy of anti-Pfs25. However, TBV effectiveness is typically evaluated using SMFA, which does not account for host-derived factors. This oversight may explain why TBVs show high blocking efficacy in laboratory settings but lower efficacy in field studies (Singh et al, 2021; Tiono et al, 2024). Therefore, we strongly advocate for revisiting the SMFA to more accurately assess TBV efficacy in blocking malaria transmission. Furthermore, our research, along with previous studies, has shown that the neutralization of TNF-α—the key factor in the pathogenesis of cerebral malaria (Gimenez et al, 2003; Grau et al, 1987)—using anti-TNF-α antibodies can effectively prevent the onset of ECM. Thus, we reveal a dual role for TNF-α. These findings suggest that modulation of TNF-α signaling may represent a potential avenue for future investigation, but that further studies are required before any clinical application can be considered.

In conclusion, we report that host-derived TNF-α facilitates malaria transmission in a cross-species manner. This finding not only enhances our understanding of malaria transmission by mosquitoes but also offers a novel strategy for preventing ECM, reducing malaria transmission, and improving the transmission-blocking efficacy of anti-Pfs25 through anti-TNF-α treatment. Furthermore, our study opens new avenues for investigating the mechanisms underlying other mosquito-borne diseases.

## Methods

**Table:** Reagents and tools table

Reagent/resource	Reference or source	Identifier or catalog number
**Experimental models**		
TNF-α^−/−^ C57BL/6J mice	Southern Medical University	/
C57BL/6J mice	GemPharmat	Strain NO. N000013
*Anopheles stephensi*	Our laboratory	/
*Plasmodium yoelii* BY265	Our laboratory	/
*Plasmodium berghei* ANKA	Our laboratory	/
*Plasmodium falciparum*	Our laboratory	/
**Antibodies**		
Anti-TNF-α Mouse IgG	Santa Cruz Biotechnology	sc-52746
InVivoMAb anti-mouse TNFα	BioXcell	BE0058
TNF-alpha Antibody (rabbit)	Cell Signaling Technology	3707S
TNF-alpha Antibody (mouse monoclonal)	Santa Cruz	sc-52746
His-Tag (D3I1O) Rabbit mAb	Cell Signaling Technology	12698
CD120a (TNF Receptor I) Monoclonal Antibody	Invitrogen	16-1202-85
CD120b (TNF Receptor II) Monoclonal Antibody	Invitrogen	14-1203-81
Alexa Flour 488 Goat Anti-Rabbit IgG	Beyotime	A0423
Alexa Flour 647 Goat Anti-Mouse IgG	Beyotime	A0473
HRP-labeled Goat Anti-Rabbit IgG	Beyotime	A0208
**Oligonucleotides and other sequence-based reagents**		
TNF-M-R	TAGGGTTAGGCTCCTGTTTCC	/
TNF-M-F	TTAGCTCTGGAGGACAGAGAAG	/
TNF-W-F	CTTCCTCCAAGAACTCAAACAGG	/
TNF-W-R	GAAGAGGCTGAGACATAGGCAC	/
Defensin-QF	CATCTGTGCCGTAGTCGTGG	/
Defensin-QR	GGCAGTTCATCCAGGAGTGTG”	/
Cecropin-A-QF	CTTCGTCTTCGTCGTTCTGG	/
Cecropin-A-QR	CTTGCCAACTCCTTCAATCTTC	/
HPX2-QF	ACCTACGCTGTCCTGTCTTATCCG	/
HPX2-QR	GCTGCACTGTACTCCGCCAAC	/
NOS-QF	CAGCGAACGGACGGCAAGCA	/
NOS-QR	TGACACGACCAGCGGCAGGAT	/
STAT-QF	GAGGTGACGGAGGTGTTCAATGC	/
STAT-QR	ATCAGGTTGCGGTTGTCGTTCTC	/
REL1-QF	GCTGTGCGAGAAGGTGGTGAAG	/
REL1-QR	GGTGGCGTTCGGAAACTGATGG	/
REL2-QF	TGACCAGTGTATGGAGGAGC	/
REL2-QR	GGAAATCAAGCAGCGAACCT	/
JNK-QF	TGGTACGACGAGAGTGAAGTTAATGC	/
JNK-QR	CCTCCTGGTAGATCAGCTCCTTCC	/
IMD-QF	TGGGACAACAAGCACAAGGA	/
IMD-QR	AACTCCGATTAGCACGGCTC	/
mTAK1-QF	AAATGTACCAACGCCCCGAT	/
mTAK1-QR	TGTTGATGTGCTGGCGGTAT	/
TEP1-QF	GTGCTGGACTACCTGAATGCGATC	/
TEP1-QR	CACCGAACGAACCATCCGACTG	/
LRIM1-QF	TAGCGTTCGTTTGTGTCGC	/
LRIM1-QR	GATGGGCGGATGCTTTCCAG	/
APL1-QF	TTTCCCTCATTCCGAGCCTGTTC	/
APL1-QR	GTTGATCCGATTGTACGAAGCATCC	/
mTRAF3-QF	TGAACGAGCCCATCCTCAC	/
mTRAF3-QR	GGAGTTTTTCCCGTTCAACC	/
mTRAF1-QF	TCTGGCGCATCGACAACTTT	/
mTRAF1-QR	TAAGGGCTGGCTTCCATCTGC	/
mTRAF4-QF	ACATCGCAGAAGGACTACGC	/
mTRAF4-QR	GCAGGTATTCAGATGTGCCTTC	/
Wengen-QF	CCGTCAGCCGTCCGTATCC	/
Wengen-QR	TCTTTCCGGCGTTCCTCCTTC	/
Grindelwald-QF	ACTGTAGTGCGTTGTGCGAA	/
Grindelwald-QR	GGTGATATTTTGACGCAATGACTTG	/
16S rDNA-QF	CAAGCGGTGGAGCATGTGGTT	/
16S rDNA-QR	ACGGCACGAGCTGACGACAA	/
S7-QF	TCGGTTCCAAGGTGATCAAAGC	/
S7-QR	AGCGCGGTCTCTTCTGCTTGT	/
dsTEP1-F	TAATACGACTCACTATAGGGAACTCGCAGGACATCAACATCACC	/
dsTEP1-R	TAATACGACTCACTATAGGGGGACGCTTCAGTGCCACCTTG	/
dsSTAT-F	TAATACGACTCACTATAGGGCGGAGACGAACTTCACCATTAAGA	/
dsSTAT-R	TAATACGACTCACTATAGGGGTTAATCTTCCACTGCGACAGATACTT	/
dsREL1-F	TAATACGACTCACTATAGGGATCTGGTCGGTAAGGAGGGC	/
dsREL1-R	TAATACGACTCACTATAGGGGGTTCCGTAAAGCGTCCTCG	/
dsIMD-F	TAATACGACTCACTATAGGGTCCAGACGTAGCGTTAGTGC	/
dsIMD-R	TAATACGACTCACTATAGGGGCTCAGCCAATGTACTGTTGT	/
dsmTAK1-F	TAATACGACTCACTATAGGGATCGTGTGAACAGCGTGCTA	/
dsmTAK1-R	TAATACGACTCACTATAGGGACTGCCCTTGTTGTTGGTCA	/
dsREL2-F	TAATACGACTCACTATAGGGCTCCACCTTGCGGTATCCAC	/
dsREL2-R	TAATACGACTCACTATAGGGCCGTTCGCTTCTCCAAACCA	/
dsmTRAF3-F	TAATACGACTCACTATAGGGCATGTCCCGTCAAAGCCAAC	/
dsmTRAF3-R	TAATACGACTCACTATAGGGTCTCCGACTGCATCAAGTGC	/
dsmTRAF1-F	TAATACGACTCACTATAGGGTGAAGCTAGATGTGGCAGCG	/
dsmTRAF1-R	TAATACGACTCACTATAGGGACCGGCGATCAAGTTACGTC	/
dsmTRAF4-F	TAATACGACTCACTATAGGGATGCAGGAGGCAAAGACGAA	/
dsmTRAF4-R	TAATACGACTCACTATAGGGCTTGCTGGGATCGACCTTCA	/
dsJNK-F	TAATACGACTCACTATAGGGCAAGCATCTCCACTCGGCTG	/
dsJNK-R	TAATACGACTCACTATAGGGTTTCGCACGGTTGGCTGTAA	/
dsWengen-F	TAATACGACTCACTATAGGGAAGCACTTTGACGCCGATATG	/
dsWengen-R	TAATACGACTCACTATAGGGTTGCTCTTGCTTCGCGTTTC	/
dsGrindelwald-F	TAATACGACTCACTATAGGGAACTTTCTGTTCAAGTCATTGCG	/
dsGrindelwald-R	TAATACGACTCACTATAGGGTTGCTCGTTGGCGTCACATT	/
**Chemicals, enzymes and other reagents**		
PBS, 1×	Servicebio	G4202
TRIzol	Ambion	15596026
VYBRANT(R) CM-DII CELL-LA	Thermo Fisher	V22888
Alexa Flour 633 phalloidin	Invitrogen	A22284
DAPI	Beyotime	C1006
Dako Fluorescence Mounting Medium	Dako	S302380
Polylysine solution	Sangon	E676002-0005
4% polyformaldehyde fixing solution	Beyotime	P0099
gentamicin sulfate	BBI	A620217-0005
Penicillin/streptomycin solution	Sangon	B540732-0010
fetal calf serum	Thermo Fisher	10091148
Heparin sodium	DING GUO PROSPEROUS	DH157
QuickBlock™ Primary Antibody Dilution Buffer for Western Blot	Beyotime	P0023A
QuickBlock™ Secondary Antibody Dilution Buffer for Western Blot	Beyotime	P0258
WB antibody rapid stripping buffer	Epizyme	PS107
5× Rapid Transfer Buffer for Ice-Free Blotting	Servicebio	G2164
EZ-buffer B 10 ×TBS buffer	Sangon Biotech	C520002
SimplePAGE Plus 15% Tris-Glycine PAGE Gel	Sangon Biotech	C651302-0001
DEPC	Biosharp	BL510A
PageRuler™ Prestained Protein Ladder	Thermo Fisher	26616-1
RPMI 1640 Medium, powder	Gibco	31800089
Xanthurenic acid	MedChemexpress (MCE)	HY-W014666
HEPES	Sigma	H4034
Albumax Ⅱ	Invitrogen	11021029
Hypoxanthine	Sigma	H9636
NaHCO_3_	Sigma	S5761
Type O positive human serum	Blood Bank of Chongqing	/
Type O positive human red blood cell	Blood Bank of Chongqing	/
TB Green® Premix Ex Taq™ II (Tli RNaseH Plus)	TaKaRa	RR820A
Tissue ROS test kit(DHE)	Bjbalb	HR8821
BGMG Fast RNAex Kit	Bioground	BG0050-L
ExonScript RT Mix	Bioground	BG0070
TB Green® Premix Ex Taq™ II (Tli RNaseH Plus)	TaKaRa	RR820A
KOD FX	TOYOBO	KFX-101
MicroElute DNA Clean Up Kit	OMEGA	D6296-01
MEGAscript™ RNAi	Invitrogen	AM1626
Mouse Inflammation Kit	BD	552364
ProFound™ Pull-Down PolyHIS Protein: Protein Interaction Ki	Thermo Fisher	21277
TEP1	LAEKISPSRNDYTITLKYKRSVRNFYINSQDINITSYEDIPEDTRALEVNVGGIGFGLLQVIYQYSLNLVNFAHRFKLDLERQSTGSEYELRMRVCANFIPKMTDSRS	/
S7	MVFGSKVIKAGNGEPDAFETQIGQAILELEMNSDLKPQLRDLYITRAREVEFNNKKAIIIYVPVPKQKAFQKVQTRLVRELEKKFSGKHVVFIAERRILPKPMRGRRDPNKQKRPRSRTLTAVYDAILEDLVFPAEVVGKRIRVKLDGSQLIKVHLDKNQQTTIEHKVDTFASVYKKLTGRDVTFEFPENYL	/
Wengen	DLGDGPGGGRSMLVRAACESRKSWWDPTLGECAPCLICADHQVVLRPCQDYMNTVCGTMKDLTASVSRPYPHLPEGNGNGIGSVGRTRDHHWKEERRKEGDGVEGYRRVVPAISTEEILWDWQ	/
Grindelwald	GCTRKCDRFEYCDENSNACRNCSALCEKDEYSCYHKCQNFLFKSLRQNITTLQES	/
**Software**		
GraphPad Prism software versions 8.0	https://www.graphpad.com/scientific-software/prism/	/
ZEN 3.4	https://www.zeiss.com.cn/corporate/home.html	/
Image J	http://imagej.nih.gov/ij/macros/	/
Primer Premier 5	http://www.premierbiosoft.com/primerdesign/	/
**Protein**		
InVivoMAb rat IgG1 isotype contro	BioXcell	BE0088
TNF-α (Mouse)	MCE	HY-P7090
TNF-α (Human)	MCE	HY-P7058
Wengen	This study	/
Grindelwald	This study	/

### Mosquito, parasites and mice

*An. stephensi* (*Anopheles stephensi*, Hori strain) from our laboratory were reared and maintained under standard laboratory conditions at 27 °C with 75% relative humidity (RH) on a 12-h light-dark cycle. *P. yoelii* BY265, *P. yoelii* BY265-RFP, *P. berghei* (ANKA strain) and *P. falciparum* were maintained in our laboratory. *P. berghei-*pfs25 transgenic parasites used in this study were kindly provided by Professor Jian Li at Xiamen University (Xiamen, China). TNF-α^−/−^ mice on the C57BL/6 genetic background were generously provided by Professor Yingping Xu of Southern Medical University (Guangzhou, China). TNF-α^−/−^ mice were crossed with WT mice of the same genetic background to obtain TNF-α^+/−^ heterozygous mice. The heterozygous mice were then crossed again, and the resulting offspring were genotyped. Genotyped littermate controls and TNF-α^−/−^ homozygous mice were used for subsequent experiments. Mice were bred and maintained in a specific pathogen-free facility at Army Medical University (Chongqing, China). All experimental procedures were conducted in strict compliance with the guidelines approved by the Experimental Animal Welfare and Ethics Committee of the Army Medical University (AMUWEC20250062).

### Mosquito infection

Female *An. stephensi* mosquitoes (3–6 days old) were starved for 24 h and then allowed to feed for 30 min on mice infected with *P. yoelii* or *P. berghei*. In each RNAi experiment, mosquitoes injected with dsLacZ or dsTarget gene were fed alternatively on a single infected mouse, which was either control or TNF-α^−/−^, for 5 min each, repeated four times. SMFA was used to infect *An. stephensi* with *P. falciparum*. In brief, the induced mature *P. falciparum* gametocytes were diluted with fresh red blood cells (RBCs), and reconstituted in 60% normal human serum in the presence or absence of rhTNF-α. The starved 3–6-day-old female mosquitoes were allowed to feed for 30 min through an artificial membrane feeding device. All manipulations were performed on a thermostatically controlled heating block at 37 °C. Engorged mosquitoes were transferred to new containers and provided with a 10% sugar solution. They were maintained at 19–20 °C for *P. berghei* or 23–24 °C for *P. yoelii*, or 26 °C for *P. falciparum*, with 70 ± 10% RH under a 12-h light/dark cycle. To quantify infection levels, mosquito midguts were dissected and the number of *P. yoelii-RFP* oocyst was counted at 2 or 7-days post-infection using a fluorescence microscope, and the oocyst numbers of both *P. berghei* and *P. falciparum* were counted at 9 days post-infection after staining with 0.1% mercurochrome. Simple randomization was performed by using a mosquito aspirator to randomly collect and allocate mosquitoes to each group, avoiding artificial selection. The outcome assessor who recorded mosquito infection and mortality rates was blinded to group allocation. Data were unblinded only after the final analysis.

### Mouse infection

Female TNF-α^−/−^ mice and control littermate mice aged 6–8 weeks were intravenously infected with 1 × 10^6^ parasitized RBCs of *P. yoelii*, *P. yoelii*-RFP or *P. berghei*. Parasitemia and gametocytemia were recorded daily by examining Giemsa-stained blood thin films under a light microscope. The clinical manifestations of mice infected with *P. berghei* were scored according to a previous study (Waknine-Grinberg et al, 2010), with a score of 2 or higher considered indicative of ECM.

### TNF-α neutralization and rescue in mice

To neutralize TNF-α, mice were injected with 100 μg of anti-mouse TNF-α antibody (BE0058, BioXcell) via the tail vein on day 2, 3 and 4 post-infection; 24 h after the last injection, the mice were subjected to mosquito feeding. To observe the impact of TNF-α neutralization on the development of ECM, mice received 400 μg of anti-TNF-α antibody intravenously on days −1, 1, 3, and 5 post-infection with *P. berghei* ANKA. For the rescue experiment, TNF-α^−/−^ mice were intravenously injected with 100 pg of recombinant mouse TNF-α protein (HY-P7090, MedChemExpress) 1 h prior to mosquito feeding on day 4 post-infection.

### Quantification of cytokines

Blood was collected from the mice on days 0, 2, 4, and 6 post-infection, and allowed to clot at room temperature for at least 1 h. The samples were then centrifuged at 3000 rpm for 10 min to obtain serum. TNF-α levels in mice were quantified using cytometric bead array (552364, BD Biosciences) according to the manufacturer’s protocols. To detect TNF-α in mosquitoes, hemolymph was collected from 15 mosquitoes by perfusion at 24 h post blood feeding, and then the concentration of TNF-α was quantified using an ELISA kit (Catalog No. m1002095V, mlbio) according to the manufacturer’s instructions.

### In vitro culture of ookinetes

After mice were infected with *P. yoelii* for 4 days, blood was collected from the orbit sinus and immediately added to the ookinete culture medium (RPMI-1640, 10% fetal calf serum (FCS), 100 μM XA (xanthurenic acid), 25 mM HEPES; pH 8.0, 100 μg/ml streptomycin, 100 U/ml penicillin). The gametocytes were cultured at 22 °C for 24 h to allow gametogenesis, fertilization, and ookinete differentiation. The generated ookinetes were calculated per 1000 RBCs in Giemsa-stained thin blood smears based on morphology, at least 50 fields were counted for each sample.

### Mosquito survival, fecundity, and fertility

To investigate whether TNF-α influences the survival rate of mosquito post-blood meal, mosquitoes fed on either control mice or TNF-α^−/−^ mice were maintained under controlled conditions of 23 °C and 70% RH. The survival status of these mosquitoes was monitored daily for a period of 15 days. To evaluate the impact of TNF-α on mosquito reproductive capacity, mosquitoes fed on either control mice or TNF-α^−/−^ mice were permitted to oviposit on moistened filter paper 3 days post-blood meal. Subsequently, the number of hatched larvae was quantified using a stereomicroscope. Additionally, to assess the direct effect of TNF-α on mosquito eggs, ten randomly selected eggs from mosquitoes fed on either control mice or TNF-α^−/−^ mice were placed in individual wells of a 12-well plate. After 3 days, the number of hatched larvae in each well was recorded.

### The amount of blood uptake

After feeding control or TNF-α^−/−^ mice, the midguts of 10 fully engorged female mosquitoes were dissected and homogenized in 200 μL PBS, respectively. The volume of blood in each midgut was determined by measuring the absorbance of protein-bound heme at 410 nm.

### Mosquito behavioral assay

The mosquito behavior assay was performed according to a previous study (Zhang et al, 2022). Briefly, sixty *An. stephensi* mosquitoes were placed in the central cage of three-cage olfactometer and were allowed them to acclimate to the environment for 30 min. Subsequently, we introduced the infected control and TNF-α^−/−^ mice into the cages at both ends. Following this, the mosquitoes were released to search for the mice. This behavioral assay was conducted under near-dark conditions. After a 30-min period, we recorded the number of mosquitoes in each end cage.

### 16S rDNA sequencing of mosquito midgut symbiont

Mosquitoes were surface-sterilized with 75% ethanol for 5 min and rinsed three times with sterile 1× PBS. Midguts were dissected aseptically, pooled into groups of 20 midguts per biological replicate (three replicates per experimental group), and homogenized. Total bacterial genomic DNA was isolated using the DNeasy PowerFecal Pro DNA Kit (QIAGEN, USA) according to the manufacturer’s protocol. DNA purity and concentration were determined spectrophotometrically (NanoDrop One, Thermo Fisher Scientific, USA) by measuring absorbance ratios at 260/280 nm and 260/230 nm. The hypervariable V3-V4 region of the bacterial 16S rRNA gene was amplified using universal primers flanked by Illumina adapters and sample-specific barcode sequences. Primary PCR products were purified with VAHTS™ DNA Clean Beads (Vazyme Biotech, China), and libraries were quantified fluorometrically using the Quant-iT™ dsDNA High-Sensitivity Assay Kit (Thermo Fisher Scientific, USA). Equimolar amounts of purified amplicons were pooled and subjected to paired-end sequencing (2 × 250 bp) on the Illumina NovaSeq 6000 platform (Illumina, USA).

### Clearance of microbiota in mosquito midgut

To clear symbiont bacteria in the midgut of *An. stephensi*, adult female mosquitoes were given access to 10% sucrose solution containing 15 μg/mL gentamicin, 100 U/mL penicillin, and 100 μg/mL streptomycin for 48 h pre-blood feeding. Midgut bacterial load verification was performed through 16S rDNA qPCR, confirming microbiota reduction compared to untreated controls.

### ROS quantification

Ten blood-fed mosquito heads were excised and transferred into 1 mL of PBS for homogenization. The homogenate was then filtered through a centrifugal filter with a 10 kDa molecular weight cut-off (431486, Corning SpinXUF, Corning) at 1000×*g* for 3 min at 4 °C. Next, 200 µL of the filtrate was added to a black, transparent-bottom 96-well cell culture plate. Dihydroethidium (DHE) was subsequently added to each sample at a volume of 2 µL. The samples were incubated at 37 °C for 30 min in the dark. Following incubation, the fluorescence intensity of DHE was measured using a microplate reader at room temperature, with an excitation wavelength of 530 nm and an emission wavelength of 610 nm. Data were normalized to total protein content (Bradford assay) and expressed as relative fluorescence units (RFU)/μg protein.

### RNA-seq in mosquitoes

Total RNA was extracted from mosquitoes that fed on control and TNF-α^−/−^ mice infected with *P. yoelii* for 4 days using TRIzol (15596018, Ambion). The samples were delivered to the Jiayin Biotech Co., Ltd. (Shanghai, China) for RNA-seq services and data analysis. Clean reads were mapped to the *Anopheles stephensi* (NCBI_GCF_013141755.1_UCI_ANSTEP_V1.0) genome database using SOAP aligner/SOAP2 mismatches. The read counts for each gene were quantified and normalized to reads per kilobase of transcript per million mapped reads (RPKM), which correlates with gene expression levels.

### Quantitative real-time PCR analysis

To quantify the bacterial load, total DNA was extracted from mosquitoes fed on *P. yoelii*-infected control or TNF-α^−/−^ mice using the DNeasy PowerFecal Pro DNA Kit (QIAGEN, USA) following the manufacturer’s protocol. DNA quality and concentration were assessed using a NanoDrop One Spectrophotometer (Thermo Fisher Scientific, USA), and 100 ng of DNA per sample was used as template for qPCR using the TB Green® Premix Ex TaqTMII (Tli RNaseH Plus, Takara Bio Inc, Japan). To quantify *SOAP* and *WARP* expression of ookinetes, midguts of mosquitoes fed on *P. yoelii*-infected control or TNF-α^−/−^ mice were dissected. Total RNA of twenty midguts was extracted using the BGMG Fast RNAex Kit (BG0050-L, Bioground) at 24 h post blood meal, and subsequently reverse transcribed into cDNA by using the ExonScript RT Mix (BG0070, Bioground) following the manufacturer’s protocol. To determine the transcriptional levels of immune-related genes, twenty mosquitoes were homogenized and total RNA was extracted and reverse transcribed into cDNA as mentioned above. The cDNA of mosquitoes was quantified via quantitative real-time PCR (qPCR) using TB Green® Premix Ex TaqTM II (RR820A, Takara). Relative quantification (ΔΔCq method) was performed using the mosquito *ribosomal protein S7 (RPS7)* gene as an endogenous control. Data are expressed as fold-changes normalized to *RPS7*. Primers used in this study could be found in the reagents and tools table.

### Western blot analysis

Hemolymph was collected from mosquitoes via perfusion. The samples were dissolved in Tissue Protein Extraction Reagent (Thermo Fisher Scientific) containing Halt Protease & Phosphatase Inhibitor Cocktail (Thermo Fisher Scientific). Equal amounts of protein were separated on a 10% SDS-PAGE gel and then transferred onto a PVDF membrane. Following blocking with a non-protein blocking solution (C530040, Sangon), the membranes were incubated overnight at 4 °C with primary antibodies against TEP1 or S7 as previously described (Zhu et al, 2022). This was followed by incubation with horseradish peroxidase (HRP)-conjugated anti-rabbit secondary antibodies (ZB-2301, Zhongshan Golden Bridge Biotechnology). Finally, the membranes were visualized using a Bio-Rad ChemiDoc Touch Imaging System.

### Gene silencing in mosquitoes

Detailed procedures for gene silencing in mosquitoes have been described previously (Oliveira et al, 2012). Specifically, adult females were injected with 69 nL dsRNA (3 mg/mL) specific for the given gene and allowed to recover for 3 days before infection. The efficiency of gene silencing was evaluated using qPCR. The primers utilized for gene detection are detailed in the reagents and tools table.

### Expression and purification of recombinant proteins

Both Wgn and Grnd protein sequences (listed in the reagents and tools table) were codon-optimized and chemically synthesized, then inserted into the expression vector pcDNA3.4. Transfection-grade plasmid DNA was prepared and transfected into CHO-S cells. The transfected cells were cultured for 5 days, after which the target protein was captured by Protein A magnetic beads and eluted with 0.1 M sodium acetate (pH 3.5). A secondary purification step was performed using size-exclusion chromatography (SEC).

### Antibody preparation

Antigenic peptide of Wgn and Grnd (listed in the reagents and tools table) was designed and synthesized by GeneCreate Biotech (Wuhan, China). Peptides were purified by high-performance liquid chromatography (HPLC) on a Waters Corporation Prominence HPLC system (Milford, MA, USA), using a Waters X Bridge C18 column (4.6 × 250 mm × 5 μm; Waters Corporation), and then conjugated to bovine serum albumin (BSA). The conjugated peptides were emulsified with Freund’s adjuvant (Sigma-Aldrich) and used to immunize specific-pathogen-free rabbits at 0, 2, 4, and 6 weeks. Seven days after the last immunization, serum was collected, and the antibody titers were determined using enzyme-linked immunosorbent assay (cat. no. 44-2404-21, Nunc 584 MaxiSorp flat bottom, Nalge Nunc International, Penfield, NY, USA). Protein G Sepharose (GE Healthcare, Chicago, IL, USA) was used to purify the polyclonal antibodies.

### Immunofluorescence analysis

Mosquito hemocytes were pre-labeled by intrathoracic injection of mosquitoes with Vybrant™ CM-DiI cell-labeling solution (V22888, Invitrogen) as previously described (Zhu et al, 2022). Twenty-four hours post injection, hemolymph was collected from mosquitoes fed on infected control or TNF-α^−/−^ mice and placed onto poly-L-lysine-coated glass slides. Hemocytes were then fixed with 4% paraformaldehyde (PFA) in PBS for 10 min at room temperature. Meanwhile, midguts were also dissected in ice-cold PBS and longitudinally opened to expose the epithelial layer, and immediately transferred to 1.5 mL microcentrifuge tubes. Tissues were fixed in 4% PFA for 2 h at 4 °C. After fixation, both hemocytes and midguts were washed three times with PBS, and were then permeabilized for 30 min in 0.1% Triton X-100/PBS. Samples were initially blocked using 10% bovine serum albumin (BSA) in PBS for 1 h at room temperature, followed by overnight incubation at 4 °C with primary antibodies diluted 1:200 in PBS containing 1% BSA: rabbit anti-Wgn polyclonal antibody. To investigate the binding of host TNF-α to Wgn in different subsets of hemocytes, mosquito hemocytes were prepared as described above, but without the pre-treatment using Vybrant™ CM-DiI. Following blocking, the hemocytes were stained with an anti-Wgn polyclonal antibody and a mouse anti-TNF-α monoclonal antibody (Santa Cruz Biotechnology, sc-52746). After thorough washing, samples were subsequently incubated for 2 h at room temperature under light-protected conditions with species-matched secondary antibodies: Alexa Fluor 488-conjugated goat anti-rabbit IgG (Beyotime Biotech, A0423), with or without Alexa Fluor 647-conjugated goat anti-mouse IgG (Beyotime Biotech, A0473), both diluted 1:500 in PBS. Nuclei were stained with 1 μg/mL DAPI (Beyotime Biotech, C1002) for 10 min. For actin visualization, midguts were incubated with 1 U phalloidin (Invitrogen, A2284) for 30 min. Samples were mounted using Dako Fluorescence Mounting Medium (S3023, Agilent) and stored at 4 °C in darkness.

### Observation of hemocytes attached to the midgut basal lamina

To investigate the recruitment of mosquito hemocytes to the midgut basal lamina, 3–5-day-old mosquitoes were allowed to feed either on the infected control or TNF-α^−/−^ mice. Immediately after blood feeding, each mosquito was injected with 69 nL of 100 μmol/L Vybrant® CM-DiI cell-labeling solution. At 24 h after blood feeding, mosquito midguts were dissected in 4% paraformaldehyde solution, longitudinally opened, and then fixed overnight in 4% paraformaldehyde at 4 °C. The next day, midguts were washed three times with PBS. Samples were initially blocked using 10% BSA in PBS for 1 h at room temperature and washed three times with PBS. For actin and nuclear staining, midguts were co-incubated with 1 U phalloidin and DAPI for 30 min at room temperature. Tissues were mounted with Dako Fluorescence Mounting Medium onto microscope slides. Hemocytes were observed using a confocal microscope (Leica TCS SP8), and image acquisition was performed using LAS AF Lite. The number of hemocytes per midgut under each biological condition was counted and compared.

### Identification and quantification of three hemocyte subsets in mosquito hemolymph

Hemolymph was collected from mosquitoes, and hemocytes were allowed to adhere to coverslips pre-coated with poly-L-lysine. Each coverslip received hemolymph from 50 mosquitoes. After adhesion, the hemocytes were fixed with 4% paraformaldehyde and stained with phalloidin and DAPI to visualize cell morphology. At least 20 random fields of view were imaged per coverslip, and cell diameters were measured using ZEN software. To classify hemocytes into distinct subtypes, the following size criteria were applied for each image as reported (Barletta et al, 2022): cells with diameters between 4 and 7.5 μm were categorized as prohemocytes, those with diameters >7.5 μm to 12.5 μm as oenocytoids, and cells with diameters >12.5 μm as granulocytes.

### His pull-down assays

For the pull-down assays, 150 µg of His-tagged Wgn or Grnd protein was incubated with HisPur™ Cobalt Resin columns at 4 °C for 2 h with gentle shaking. Following five washes with wash buffer, 150 µg of human recombinant TNF-α (HY-P7058, MedChemExpress) was added and incubated overnight at 4 °C with continuous mixing. After an additional five washes with wash buffer, proteins were eluted using Elution Buffer containing 290 mM imidazole. The eluates were then separated by 15% SDS-PAGE, transferred to PVDF membranes, and probed with anti-His antibody (12698 T, Cell Signaling Technology) and anti-TNF-α antibody (3707S, Cell Signaling Technology). This was followed by incubation with horseradish peroxidase (HRP)-conjugated anti-rabbit secondary antibodies (ZB-2301, Zhongshan Golden Bridge Biotechnology). Finally, the membranes were visualized using a Bio-Rad ChemiDoc Touch Imaging System.

### Surface plasmon resonance

All SPR experiments were performed on a Biacore T200 at a constant temperature of 25 °C. A CM5 sensor chip was used, and an anti‑His‑tag antibody was covalently immobilized onto the chip surface via a standard amine‑coupling kit. Specifically, a mixture of 50 mM N‑hydroxysuccinimide (NHS) and 200 mM 1‑ethyl‑3‑(3‑dimethylaminopropyl) carbodiimide (EDC) was injected at a flow rate of 10 μL/min for 7 min to activate the chip channels. The anti‑His antibody was diluted to 50 μg/mL in 10 mM sodium acetate buffer (pH 5.0) and injected at 10 μL/min for 7 min to achieve an immobilization level of approximately 6000–8000 RU. Finally, the surface was blocked with 1 M ethanolamine (pH 8.5) for 7 min. A blank channel without immobilized antibody was used as a reference. The running buffer was 10 mM HEPES, 150 mM NaCl, 0.05% Tween‑20, 50 μM EDTA, pH 7.4. The system was equilibrated at a flow rate of 30 μL/min before the experiment. His‑tagged Wgn or Grnd protein was diluted to an appropriate concentration in the running buffer and injected at 10 μL/min for 60–120 s to capture ~300–500 RU on the surface. Subsequently, TNF‑α protein was serially diluted in the running buffer to concentrations of 1, 2, 4, 8, 16, and 32 nM and injected sequentially at a flow rate of 30 μL/min, with an association time of 120 s and a dissociation time of 300–600 s. After each cycle, a regeneration buffer was injected at 30 μL/min for 30 s to completely remove the surface‑bound TNF‑α, and the next concentration was analyzed after regeneration.

### ELISA saturation concentration method

Add 100 μL of Wgn or Grnd protein at a concentration of 10 μg/mL to each well of a 96-well microplate and coat overnight at 4 °C. After discarding the coating solution, add 200 μL of PBST washing buffer to each well, let stand for 3 min, discard, and repeat the wash three times before patting dry. Subsequently, add 200 μL of 1% BSA/PBS blocking buffer to each well and block at 37 °C for 1 h. Serially dilute recombinant TNF-α protein in 0.5% BSA/PBS dilution buffer to final concentrations of 0, 1, 5, 10, 50, 100, 500, and 1000 nM. Discard the blocking buffer and immediately add 100 μL of the prepared TNF-α solution to each well, then incubate at 37 °C for 2 h. Remove the liquid, add 200 μL of PBST to each well, wash for 3 min, repeat three times, and pat dry. Dilute the anti-TNF-α-HRP antibody at 1:5000, add 100 μL to each well, and incubate at 37 °C for 1 h. Discard the liquid, add 200 μL of PBST, wash for 3 min, repeat five times, and pat dry. Finally, add 100 μL of TMB substrate solution to each well, incubate at room temperature in the dark for 15 min, add 50 μL of stop solution, and measure the absorbance at 450 nm using a microplate reader.

### *P. falciparum* culture and gametocytes induction

The *P. falciparum* NF54 strain was cultured for mature gametocyte production using established protocols (Tripathi et al, 2020). Briefly, gametocyte cultures were initiated at 0.3-1% parasitemia in 4% hematocrit using RPMI-1640 medium supplemented with 25 mM HEPES, 100 μg/mL hypoxanthine, 25 mM NaHCO3, 5 μg/mL gentamicin, and 10% human serum (pH 7.4), with daily medium replacement without fresh erythrocyte supplementation. Both human blood and serum were anonymously obtained from Chongqing Blood Bank, and the related experiments were reviewed and approved by the Ethics Committee of the Army Medical University Institute of Medical Research (AF/SC-08/1.0). Developmental progression and culture purity were monitored through Giemsa-stained blood smears analyzed every 48 h over 15–18 days.

### Assessment of blocking efficacy of anti-Pfs25 antibody

The anti-Pfs25 monoclonal antibody was generated through recombinant expression technology by GenScript (Shanghai, China) as previously described(He et al, 2025). To assess the influence of TNF-α on the transmission blockade efficacy of anti-Pfs25 antibody, control and TNF-α^−/−^ mice were intravenously inoculated with 4 × 10⁶ *P. berghei*-*Pfs25*-pRBCs. Three days later, mice were intravenously injected with 20 μg of anti-Pfs25 antibody and then fed to mosquitoes. Oocyst number per mosquito was counted at 9 days post blood meal, and both TRA (%) and TBA (%) were calculated as previously described(He et al, 2025).

### Statistical analysis

All statistical analyses were conducted using GraphPad Prism (GraphPad Software, La Jolla, CA, USA). For continuous variables, unpaired *t* test (two-tailed) was employed to compare two groups when data followed a normal distribution; otherwise, the Mann–Whitney *U* test (two-tailed) was utilized for group comparisons. The incidence of ECM was measured by log-rank (Mantel–Cox) test. Parasitemia and gametocytemia were analyzed by two-way ANOVA mixed model. Differences in mosquito infection rates were analyzed using Fisher’s exact test (two-sided). The significance level was set at *P* < 0.05. Error bars indicate the SD.

## Supplementary information

Peer Review File

Source data Fig. 1

Source data Fig. 2

Source data Fig. 3

Source data Fig. 4

Source data Fig. 5

Source data Fig. 6

Source data Fig. 7

Figure EV1 Source Data

Figure EV2 Source Data

Figure EV3 Source Data

Figure EV4 Source Data

Figure EV5 Source Data

Figure EV6 Source Data

Figure EV7 Source Data

Expanded View Figures

## Data Availability

The 16S rDNA sequence data and RNA sequencing datasets have been deposited in the National Center for Biotechnology Information (NCBI) Sequence Read Archive under the accession number PRJNA1263103. https://trace.ncbi.nlm.nih.gov/Traces/?view=run_browser&acc=SRR33576267&display=data-access. The source data of this paper are collected in the following database record: biostudies:S-SCDT-10_1038-S44319-026-00929-8.
