# Supplementary material for: Cross-species TNF-α signaling in mosquitoes promotes malaria transmission
Source: EMBO Rep. 2026 Sep 29;27(21):6191–222. doi: 10.1038/s44319-026-00929-8 (PMC13743784; doi:10.1038/s44319-026-00929-8)
Supplement: Supplementary file 1 [file 44319_2026_929_MOESM16_ESM.pdf]

Expanded View Figures

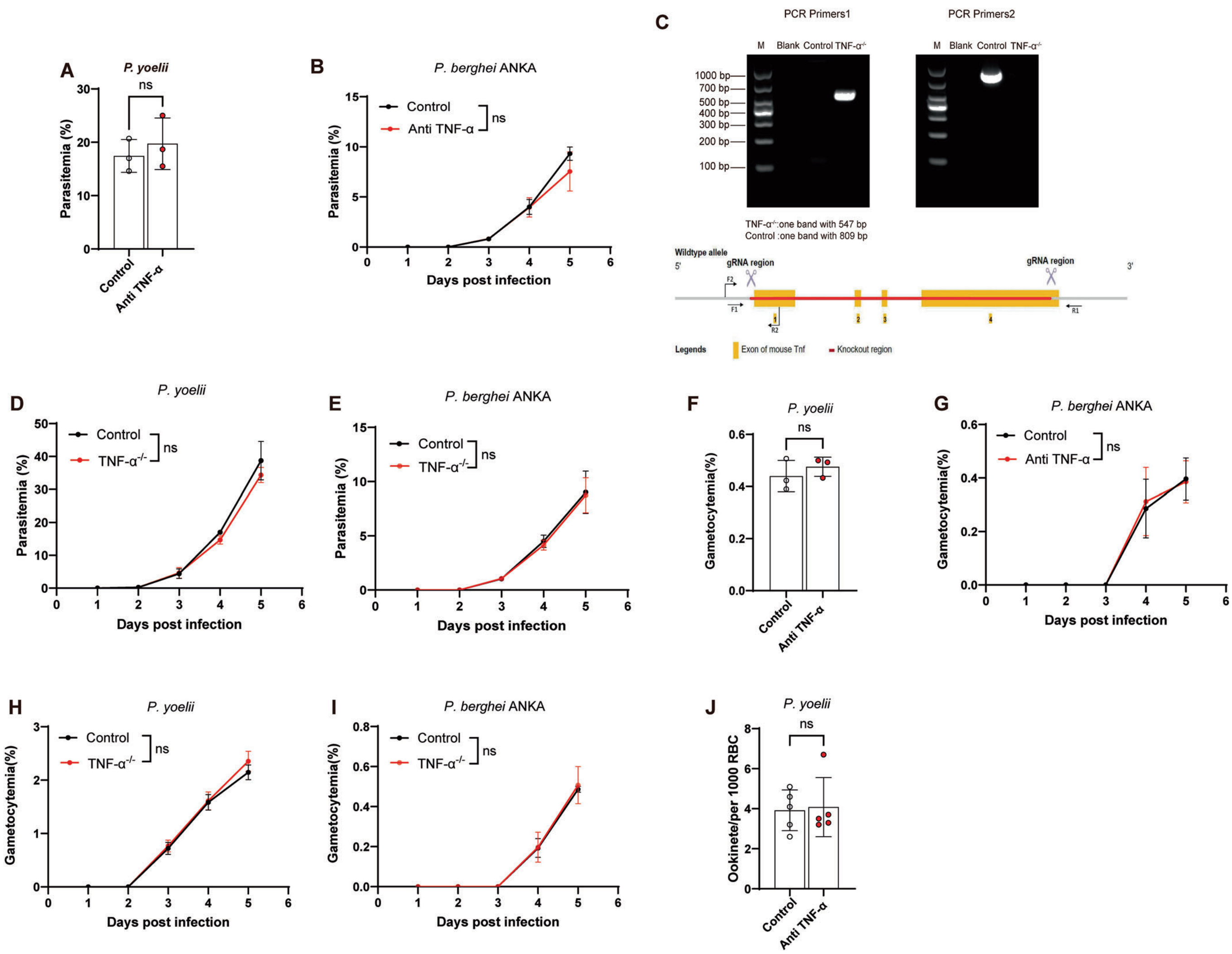

Figure EV1. Effect of TNF-α on parasite growth in mice.

(**A**) Parasitemia of *P. yoelii* in mice pre-treated with or without anti-TNF-α antibody at 4 days post-infection. Results are from $n = 3$ biological replicates. (**B**) Parasitemia of *P. berghei* in mice pre-treated with or without anti-TNF-α. Results are from $n = 5$ biological replicates. (**C**) Identification of TNF-α knockout mice. Different lengths of fragments amplified from the genomic DNA extracted from control and transgenic mice. A 547 bp target fragment was obtained from the genomic DNA extracted from TNF-α knockout mice while no band was obtained from the genomic DNA extracted from control mice with Primers 1. A 809 bp target fragment was obtained from the genomic DNA extracted from control mice while no band was obtained from genomic extracted from TNF-α knockout mice with Primers 2. (**D, E**) Parasitemia of *P. yoelii* (**D**) and *P. berghei* (**E**) in control and TNF-α$^{-/-}$ mice Results are from $n = 3$ biological replicates. (**F**) Gametocytemia of *P. yoelii* in mice pre-treated with or without anti-TNF-α antibody at 4 days post-infection. Results are from $n = 3$ biological replicates. (**G**) Gametocytemia of *P. berghei* in mice pre-treated with or without anti-TNF-α antibody. Results are from $n = 5$ biological replicates. (**H, I**) Gametocytemia of *P. yoelii* (**H**) and *P. berghei* (**I**) in control and TNF-α$^{-/-}$ mice. Results are from $n = 3$ biological replicates. (**J**) Blood taken from *P. yoelii*-infected mice pre-treated with or without anti-TNF-α antibody was in vitro cultured for 24 h, and the number of generated ookinetes per 1000 RBCs was counted and compared. Results are from $n = 5$ biological replicates. Data information: (**A, B, D–J**) Data are presented as the mean ± SD. Statistical analysis of data in (**A, F, J**) was performed by unpaired *t* test (two-tailed), while data in (**B, D, E, G–I**) was analyzed by two-way ANOVA mix model. ns no significance. Source data are available online for this figure.

 © The Author(s)

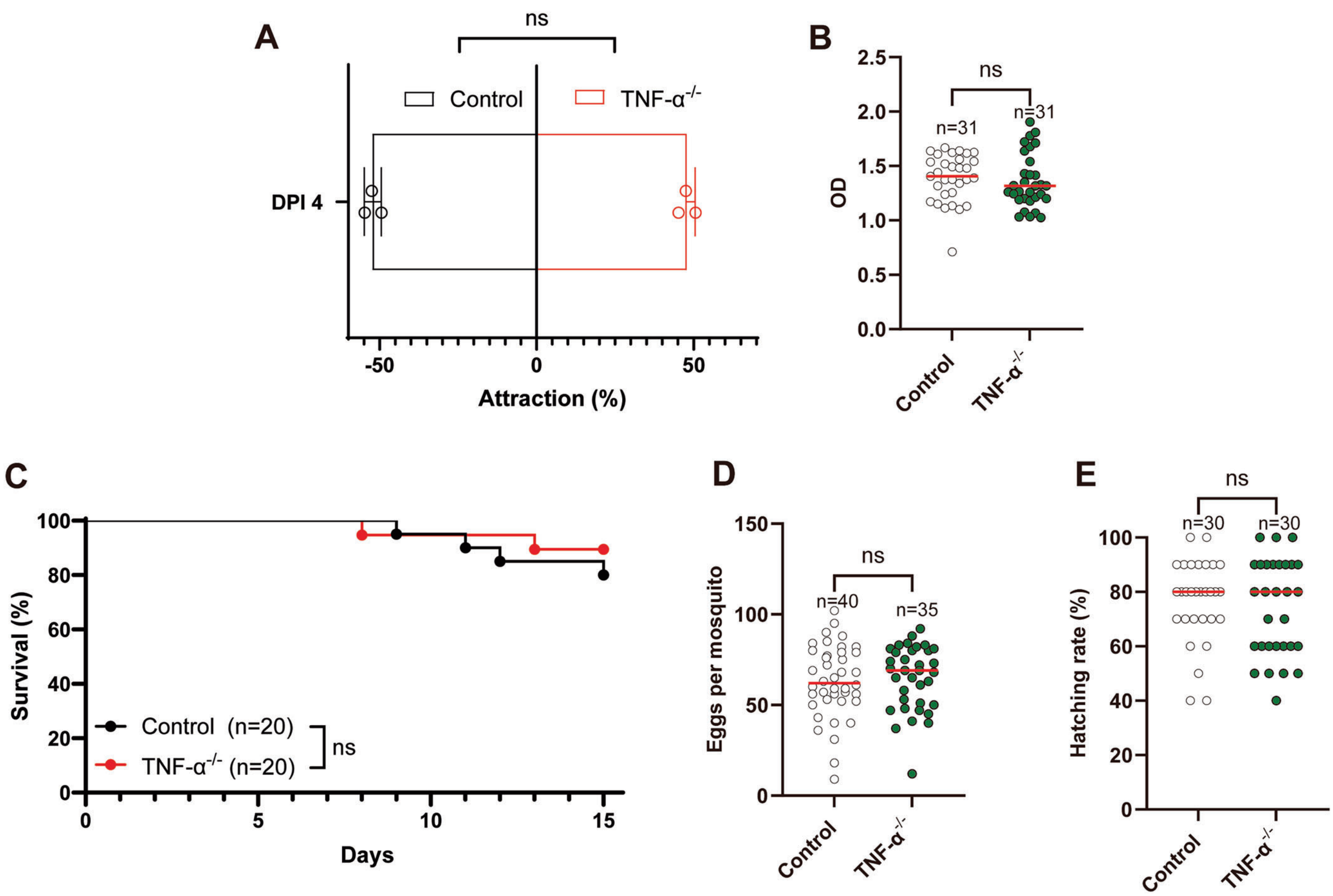

Figure EV2. Effect of TNF-α on mosquito fitness.

(**A**) Host-seeking behavior of mosquitoes toward littermate control or TNF-α$^{-/-}$ mice infected with *P. yoelii* for 4 days was assessed through dual-choice olfactometry assays. A box plot shows the choice preference of the mosquitoes in Control and TNF-α$^{-/-}$ groups. The central line represents the median (50th percentile). The box boundaries represent the 25th and 75th percentiles (Q1 and Q3, interquartile range, IQR). The whiskers extend to the minimum and maximum observed values within 1.5 × IQR from the lower and upper quartiles; since all data points lie within this range, the whisker endpoints coincide with the data extrema and no outliers are present. Specific descriptive values are as follows: Control: Min = 49.4, Q1 = 49.4, Median = 52.5, Q3 = 54.8, Max = 54.8. TNF-α$^{-/-}$: Min = 45.2, Q1 = 45.2, Median = 47.5, Q3 = 50.6, Max = 50.6. Results are from $n = 3$ biological replicates. (**B**) Midguts of mosquitoes fed on infected control or TNF-α$^{-/-}$ mice were dissected. Hemoglobin-derived heme levels were quantified by measuring absorbance at 410 nm. Individual dots represent the OD value of ten midguts, with a line indicating median. (**C**) Survival probability of mosquitoes fed on infected control or TNF-α$^{-/-}$ mice within 15 days. (**D**) The number of eggs laid by each mosquito fed on infected control or TNF-α$^{-/-}$ mice. Individual dots represent the number of eggs laid by per mosquito, with a line indicating the median. (**E**) The percentage of hatched eggs collected from mosquitoes fed on infected control or TNF-α$^{-/-}$ mice. Individual dots represent the hatching rate of ten eggs, with a line indicating the median. Results are from $n = 30$ biological replicates. Data information: For (**B–D**), *n* indicates the number of biologically independent mosquito samples. Statistical analysis of data in (**A**) was performed by Fisher's exact test (two-sided), while data in (**B**, **D**, **E**) was analyzed by Mann–Whitney *U* test (two-tailed). In panel (**C**), data was analyzed by log-rank (Mantel–Cox) test, ns: no significance. Source data are available online for this figure.

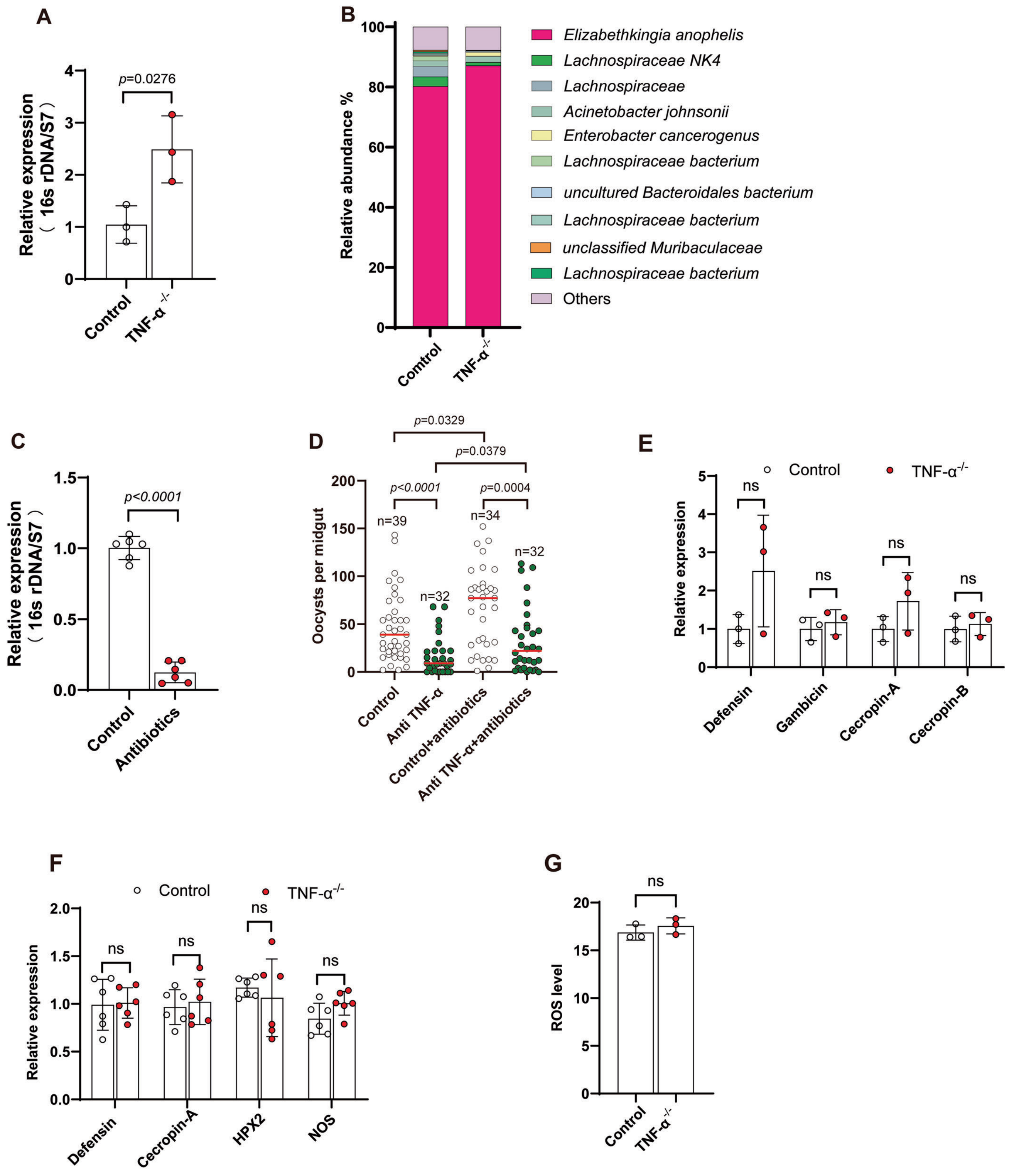
A
Relative expression
(16s rDNA/S7)
p=0.0276
Control
TNF-α -/-
B
Relative abundance %
Elizabethkingia anophelis
Lachnospiraceae NK4
Lachnospiraceae
Acinetobacter johnsonii
Enterobacter cancerogenus
Lachnospiraceae bacterium
uncultured Bacteroidales bacterium
Lachnospiraceae bacterium
unclassified Muribaculaceae
Lachnospiraceae bacterium
Others
Comtrol
TNF-α -/-
C
Relative expression
(16s rDNA/S7)
p<0.0001
Control
Antibiotics
D
Oocysts per midgut
p=0.0329
p=0.0379
p<0.0001
p=0.0004
n=39
n=32
n=34
n=32
Control
Anti TNF-α
Control+antibiotics
Anti TNF-α+antibiotics
E
Control
TNF-α-/-
Relative expression
ns
Defensin
Gambicin
Cecropin-A
Cecropin-B
F
Control
TNF-α-/-
Relative expression
ns
Defensin
Cecropin-A
HPX2
NOS
G
ROS level
ns
Control
TNF-α -/-

 © The Author(s)

Figure EV3. The alteration of microbiota, AMPs and ROS is not contributed to the promoting effect of TNF-α on malaria infection.

(**A**) The abundance of microbiota in midguts of mosquitoes feeding on *P. yoelii*-infected control or TNF-$\alpha^{-/-}$ mice was determined by qPCR at 24 h post-blood meal. Results are from $n = 3$ biological replicates. (**B**) Comparative analysis of midgut microbial communities in mosquitoes feeding on infected control and TNF-$\alpha^{-/-}$ mice at 24 h post-blood meal. (**C**) The abundance of microbiota in the mosquito midgut was assessed by qPCR before and after 4-day antibiotic treatment. Results are from $n = 6$ biological replicates. (**D**) After microbiota was cleared by antibiotics treatment, mosquitoes were allowed to feed on *P. yoelii*-infected control and TNF-$\alpha^{-/-}$ mice. Seven days later, the oocysts number in mosquitoes were counted and compared. The number of oocysts per mosquito represented by individual dots, with a horizontal line indicating the median, and *n* indicating the number of biologically independent mosquito samples. (**E**) Relative expression of *Defensin*, *Gambicin, Cecropin-A* and *Cecropin-B* between mosquitoes fed on *P. yoelii*-infected control and TNF-$\alpha^{-/-}$ mice. Results are from $n = 3$ biological replicates. (**F**) Relative expression of *Defensin*, *Cecropin-A*, HPX2 and NOS between hemocytes from mosquitoes fed on *P. yoelii*-infected control and TNF-$\alpha^{-/-}$ mice. Results are from $n = 6$ biological replicates. (**G**) ROS levels in midguts of mosquitoes feeding on *P. yoelii*-infected control and TNF-$\alpha^{-/-}$ mice was quantified by DHE assays 24 h post blood meal. Data were normalized to total protein content (Bradford assay) and expressed as relative fluorescence units (RFU)/μg protein. Results are from $n = 3$ biological replicates. Data information: Data in panels of (**A**, **C**, **E–G**) are presented as the mean ± SD. Statistical analysis of data in (**A**, **C**, **E–G**) were performed by unpaired *t* test (two-tailed), while data in (**D**) was analyzed by Mann–Whitney *U* test (two-tailed). Source data are available online for this figure.

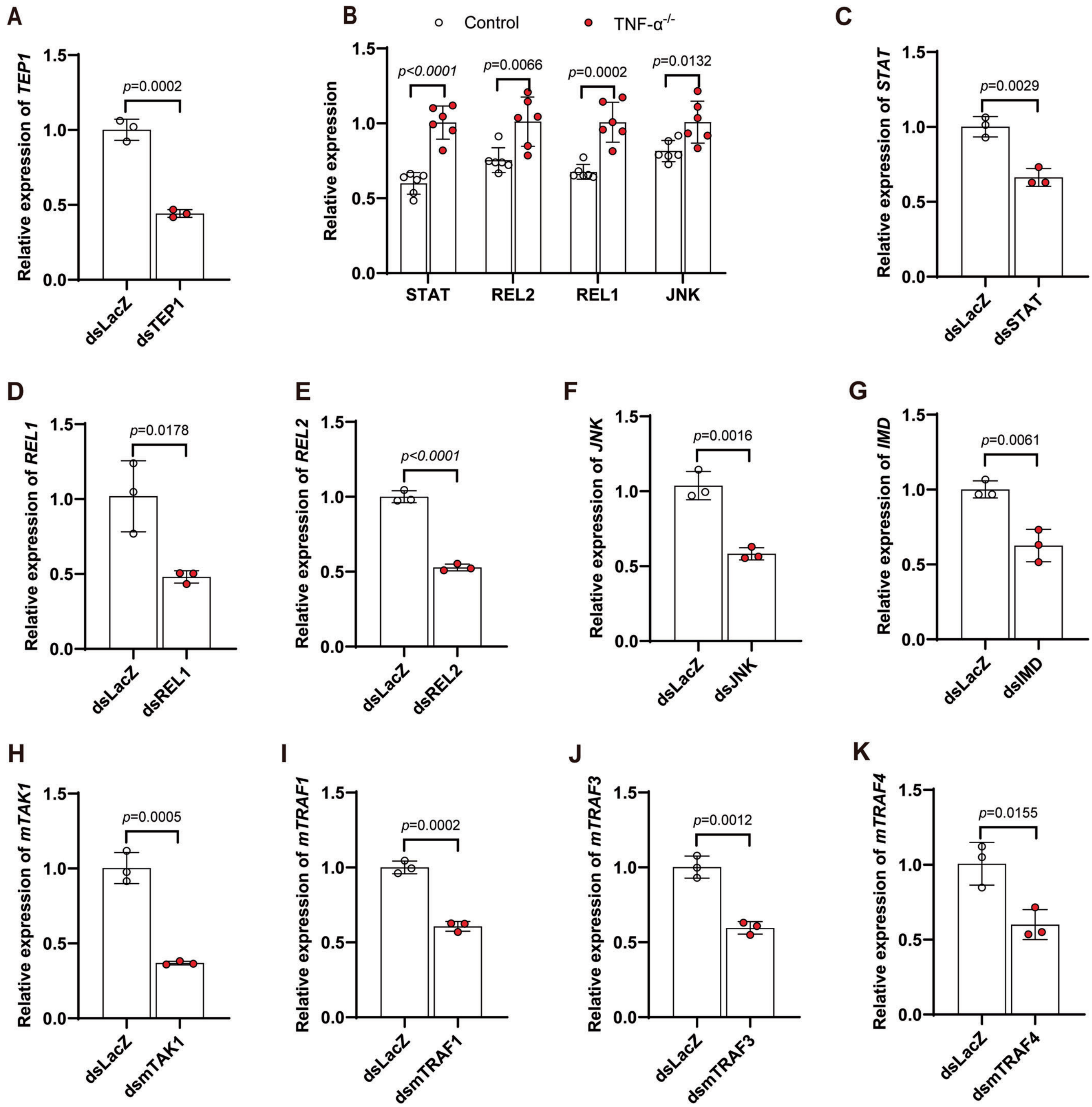

Figure EV4. The dsRNA mediated silencing efficiency of *TEP1*, *STAT*, *REL1*, *REL2*, *JNK*, *IMD*, *mTAK1*, *mTRAF1*, *mTRAF3* and *mTRAF4*.

(**A**, **C–K**) The dsRNA silencing efficiency of *TEP1* (**A**), *STAT* (**C**), *REL1* (**D**), *REL2* (**E**), *JNK* (**F**), *IMD* (**G**), *mTAK1* (**H**), *mTRAF1* (**I**), *mTRAF3* (**J**) and *mTRAF4* (**K**) was assessed by qPCR. Results are from $n = 3$ biological replicates. (**B**) Relative expression of *STAT, REL2, REL1* and *JNK* between hemocytes from mosquitoes fed on *P. yoelii*-infected control and TNF-α$^{-/-}$ mice. Results are from $n = 6$ biological replicates. Data are presented as the mean ± SD. Statistical analysis of data was conducted by unpaired *t* test (two-tailed). Source data are available online for this figure.

 © The Author(s)

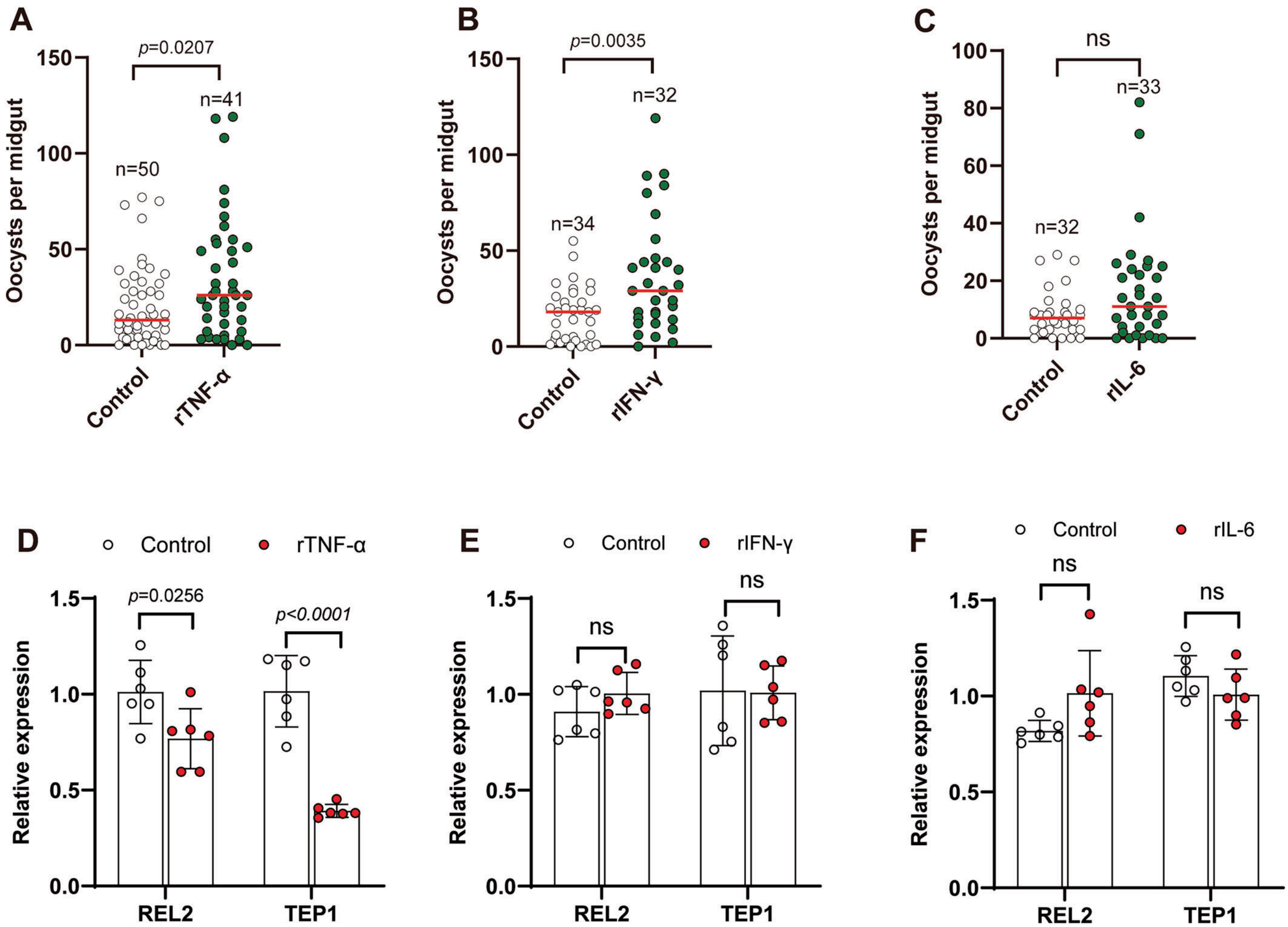

Figure EV5. The effect of rTNF-α, rIFN-γ and rIL-6 on the parasite infection and the expression of REL2 and TEP1 in mosquitoes.

(**A–C**) Blood was collected from mice infected with *P. yoelii* on day 4. Oocyst counts in midgut of mosquitoes fed on blood supplemented with or without rTNF-α (**A**), rIFN-γ (**B**), or rIL-6 (**C**). The number of oocysts per mosquito is represented by individual dots, with a horizontal line indicating the median, and n indicating the number of biologically independent mosquito samples. (**D–F**) Relative expression levels of *REL2* and *TEP1* in mosquitoes fed on blood supplemented with or without rTNF-α (**D**), rIFN-γ (**E**) or rIL-6 (**F**). Data are presented as the mean ± SD. Results are from $n = 6$ biological replicates. Data information: Statistical analysis of data in (**A–C**) was performed using Mann–Whitney *U* test (two-tailed), while data in (**D–F**) were analysed by unpaired *t* test (two-tailed). ns: no significance. Source data are available online for this figure.

© The Author(s)

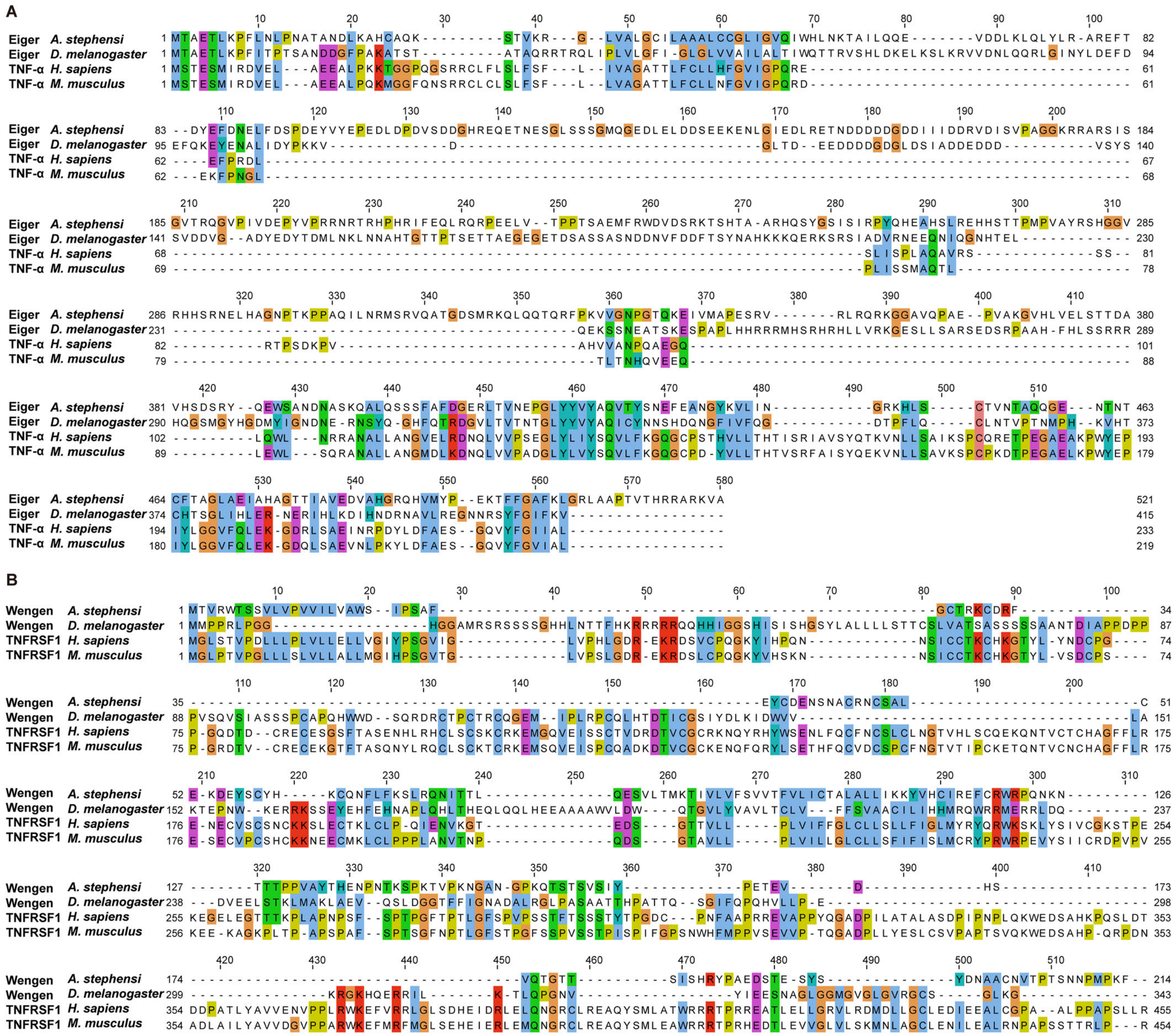

Figure EV6. Sequence alignment of Eiger and Wgn with their homologs in mammalians.

(**A**) Sequence alignment of the Eiger from *An. stephensi* and *D. melanogaster* with TNF-α from *M. musculus* and *H. sapiens*. (**B**) Sequence alignment of the Wgn from *An. stephensi* and *D. melanogaster* with TNFRs from *M. musculus* and *H. sapiens*. Different colors indicate varying levels of homology among the alignments. Source data are available online for this figure.

 © The Author(s)

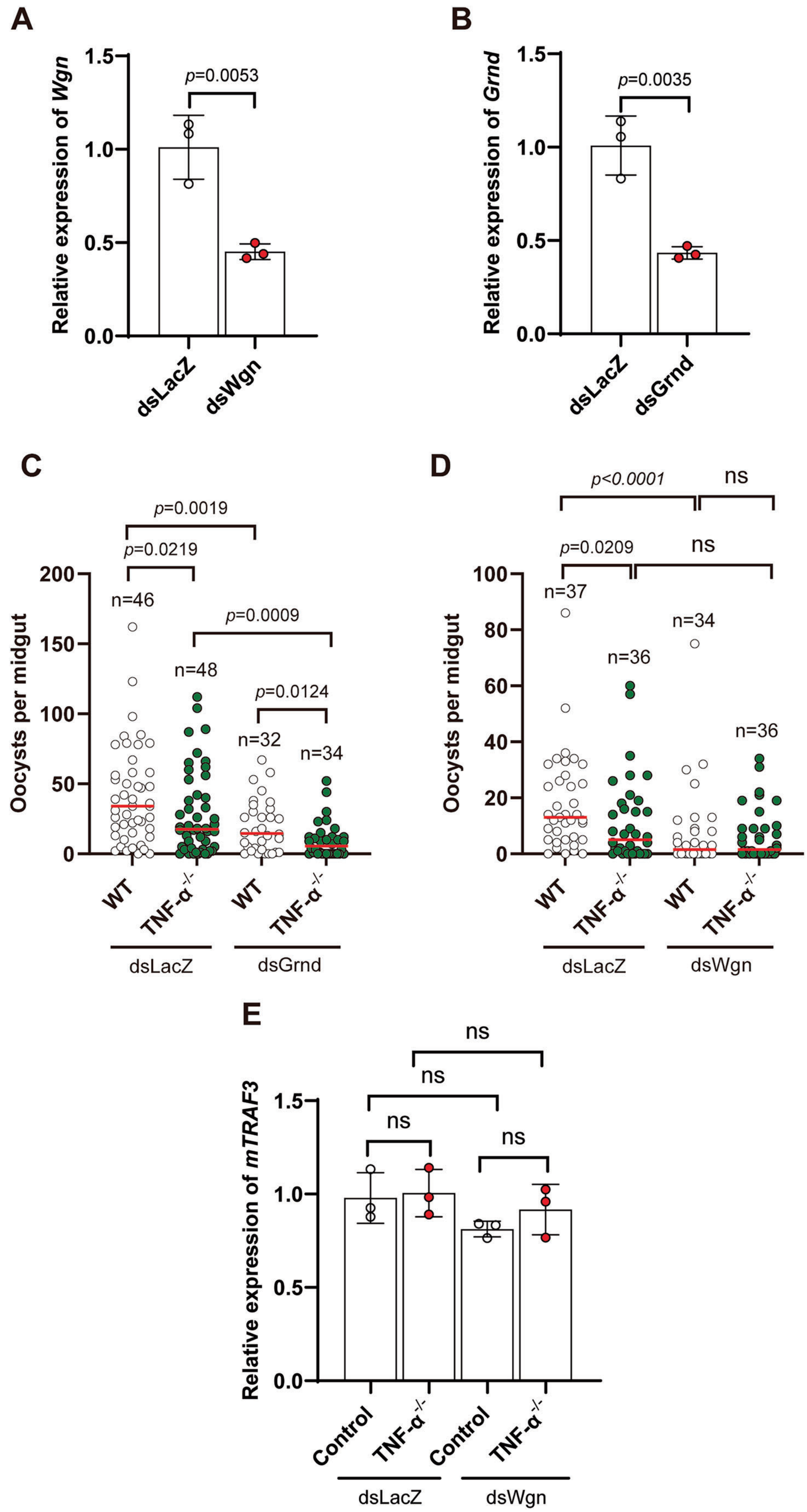
A
Relative expression of Wgn
p=0.0053
1.5
1.0
0.5
0.0
dsLacZ
dsWgn
B
Relative expression of Grnd
p=0.0035
1.5
1.0
0.5
0.0
dsLacZ
dsGrnd
C
Oocysts per midgut
p=0.0019
p=0.0219
p=0.0009
p=0.0124
200
150
100
50
0
n=46
n=48
n=32
n=34
WT
TNF-α-/-
WT
TNF-α-/-
dsLacZ
dsGrnd
D
Oocysts per midgut
p<0.0001
ns
p=0.0209
ns
100
80
60
40
20
0
n=37
n=36
n=34
n=36
WT
TNF-α-/-
WT
TNF-α-/-
dsLacZ
dsWgn
E
Relative expression of mTRAF3
ns
ns
ns
ns
1.5
1.0
0.5
0.0
Control
TNF-α-/-
Control
TNF-α-/-
dsLacZ
dsWgn

Figure EV7. The effect of knockdown of *Wgn* or *Grnd* on *P. berghei* infection in mosquitoes.

(**A**, **B**) The dsRNA silencing efficiency of *Wgn* (**A**) and *Grnd* (**B**) was determined by qPCR. Results are from $n = 3$ biological replicates. (**C**, **D**) *Grnd* (**C**) or *Wgn* (**D**) of mosquitoes was knocked down, and mosquitoes were then fed on *P. berghei*-infected control or TNF-α$^{-/-}$ mice. Oocyst number in mosquito midguts was counted at 7 days post-blood meal. The number of oocysts per mosquito is represented by individual dots, with a horizontal line indicating the median, and n indicating the number of biologically independent mosquito samples. (**E**) The relative expression levels of *mTRAF3* in mosquitoes with *Wgn* knocked down were evaluated by qPCR at 24 h post-blood feeding. Results are from $n = 3$ biological replicates. Data information: Data in (**A**, **B**, **E**) are presented as the mean ± SD. Statistical analysis of data in (**A**, **B**, **E**) was done by unpaired *t* test (two-tailed), while data in (**C**, **D**) was analysed using a Mann–Whitney *U* test (two-tailed). ns no significance. Source data are available online for this figure.

 © The Author(s)
