# Supplementary material for: Cross-species TNF-α signaling in mosquitoes promotes malaria transmission
Source: EMBO Rep. 2026 Sep 29;27(21):6191–222. doi: 10.1038/s44319-026-00929-8 (PMC13743784; doi:10.1038/s44319-026-00929-8)
Supplement: Supplementary file 2 [file 44319_2026_929_MOESM1_ESM.pdf]

EMBO reports

Cross-species TNF-α signaling in mosquitoes promotes malaria transmission

Meilin Li, Biao He, Jian Zhang, Yongling Fan, Jianyong Li, Feng Zhu, Shuai Guo, Taoli Zhou, Yuanli Gao, Taiping Liu, and Wenyue Xu

Corresponding author(s): Wenyue Xu (xuwenyue@tmmu.edu.cn)

Review Timeline:

Submission Date:	4th Dec 25
Editorial Decision:	19th Jan 26
Revision Received:	18th Apr 26
Editorial Decision:	19th Jun 26
Revision Received:	14th Jul 26
Editorial Decision:	24th Aug 26
Revision Received:	27th Aug 26
Accepted:	28th Aug 26

Editor: Achim Breiling

Transaction Report:

(Note: With the exception of the correction of typographical or spelling errors that could be a source of ambiguity, letters and reports are not edited. Depending on transfer agreements, referee reports obtained elsewhere may or may not be included in this compilation. Referee reports are anonymous unless the Referee chooses to sign their reports.)

Dear Prof. Xu,

Thank you for the transfer of your manuscript to EMBO reports. I have now received reports from the three referees that were asked to evaluate your study, which can be found at the end of this email. As you will see, the referees have several comments, concerns, and suggestions, indicating that a major revision of the manuscript is necessary to allow publication of the study in EMBO reports. As the reports are below, and all points need to be addressed, I will not detail them here.

Moreover, considering the low sample size of several experiments, please refrain from stating in each figure legend repeatedly that "three independent experiments have been performed with similar results for each experiment", but rather show these replicate experiments were applicable to increase the reliability of the data.

Given the constructive referee comments, I would thus like to invite you to revise your manuscript with the understanding that the concerns of the referees must be addressed in the revised manuscript and in a detailed point-by-point response. Acceptance of your manuscript will depend on a positive outcome of a second round of review. It is EMBO reports policy to allow a single round of revision only and acceptance of the manuscript will therefore depend on the completeness of your responses included in the next, final version of the manuscript.

Revised manuscripts should be submitted within three months of a request for revision. Please contact me to discuss the revision (also by video chat) if you have questions or comments regarding the revision, or should you need additional time.

When submitting your revised manuscript, please also carefully review the instructions that follow below.

PLEASE NOTE THAT upon resubmission revised manuscripts are subjected to an initial quality control prior to exposition to re-review. Upon failure in the initial quality control, the manuscripts are sent back to the authors, which may lead to delays. Frequent reasons for such a failure are the lack of the data availability section (please see below) and the presence of statistics based on n=2 (the authors are then asked to present scatter plots or provide more data points).

When submitting your revised manuscript, we will require:

1) a .docx formatted version of the final manuscript text (including legends for main figures, EV figures and tables), but without the figures included. Figure legends should be compiled at the end of the manuscript text.

2) individual production quality figure files as .eps, .tif, .jpg (one file per figure), of main figures and EV figures. Please upload these as separate, individual files upon re-submission.

The Expanded View format, which will be displayed in the main HTML of the paper in a collapsible format, has replaced the Supplementary information. You can submit up to 6 images as Expanded View. Please follow the nomenclature Figure EV1, Figure EV2 etc. The figure legend for these should be included in the main manuscript document file in a section called Expanded View Figure Legends after the main Figure Legends section. Additional Supplementary material should be supplied as a single pdf file labeled Appendix. The Appendix should have page numbers and needs to include a table of content on the first page (with page numbers) and legends for all content. Please follow the nomenclature Appendix Figure Sx, Appendix Table Sx etc. throughout the text, and also label the figures and tables according to this nomenclature.

For more details, please refer to our guide to authors:
https://link.springer.com/journal/44319/submission-guidelines#cms-Revised-submissions

Please consult our guide for figure preparation:
https://media.springernature.com/original/springer-cms/rest/v1/content/27825798/data/v1

3) a .docx formatted letter INCLUDING the reviewers' reports and your detailed point-by-point responses to their comments. As part of the EMBO Press transparent editorial process, the point-by-point response is part of the Review Process File (RPF), which will be published alongside your paper.

4) a complete author checklist, which you can download from our author guidelines (https://link.springer.com/journal/44319/submission-guidelines#cms-Revised-submissions). Please insert page numbers in the checklist to indicate where the requested information can be found in the manuscript. The completed author checklist will also be part of the RPF.

Please also follow our guidelines for the use of living organisms, and the respective reporting guidelines:
https://link.springer.com/partners/embo-press/editorial-policies#Animal%20and%20human%20studies

5) that primary datasets produced in this study (e.g. RNA-seq, ChIP-seq, structural and array data) are deposited in an

appropriate public database. If no primary datasets have been deposited, please also state this in a dedicated section (e.g. 'No primary datasets have been generated and deposited'), see below.

See also: https://link.springer.com/partners/embo-press/editorial-policies#Data%20deposition

Please remember to provide a reviewer password if the datasets are not yet public.

The accession numbers and database should be listed in a formal "Data Availability" section that follows the model below. This is now mandatory (like the COI statement). Please note that the Data Availability Section is restricted to new primary data that are part of this study. This section is mandatory. As indicated above, if no primary datasets have been deposited, please state this in this section

Data availability

The datasets produced in this study are available in the following databases:

- RNA-Seq data: Gene Expression Omnibus GSE46843 (https://www.ncbi.nlm.nih.gov/geo/query/acc.cgi?acc=GSE46843)
- [data type]: [name of the resource] [accession number/identifier/doi] ([URL or identifiers.org/DATABASE:ACCESSION])

*** Note - All links should resolve to a page where the data can be accessed. ***

See also: https://link.springer.com/partners/embo-press/editorial-policies#Data%20availability%20statement

Moreover, I have these editorial requests:

6) We now request the publication of original source data with the aim of making primary data more accessible and transparent to the reader. You will receive a separate email with instructions for providing source data with your revised manuscript, including information how to upload and organize the files.

7) Our journal encourages inclusion of *data citations in the reference list* to directly cite datasets that were re-used and obtained from public databases. Data citations in the article text are distinct from normal bibliographical citations and should directly link to the database records from which the data can be accessed. In the main text, data citations are formatted as follows: "Data ref: Smith et al, 2001" or "Data ref: NCBI Sequence Read Archive PRJNA342805, 2017". In the Reference list, data citations must be labeled with "[DATASET]". A data reference must provide the database name, accession number/identifiers and a resolvable link to the landing page from which the data can be accessed at the end of the reference. Further instructions are available at: https://link.springer.com/journal/44319/submission-guidelines#cms-Reference-guidelines

8) Regarding data quantification and statistics, please make sure that the number "n" for how many independent experiments were performed, their nature (biological versus technical replicates), the bars and error bars (e.g. SEM, SD) and the test used to calculate p-values is indicated in the respective figure legends (also for EV and Appendix figures). Please also check that all the p-values are explained in the legend, and that these fit to those shown in the figure. Please provide statistical testing where applicable. Please avoid the phrase 'independent experiment' but clearly state if these were biological or technical replicates. Please also indicate (e.g. with n.s.) if testing was performed, but the differences are not significant. In case n=2, please show the data as separate datapoints without error bars and statistics. See also: https://link.springer.com/journal/44319/submission-guidelines#cms-Figure-and-data-presentation

If n<5, please show single datapoints for diagrams.

9) Please add scale bars of similar style and thickness to all microscopic images, using clearly visible black or white bars (depending on the background). Please place these in the lower right corner of the images themselves. Please do not write on or near the bars in the image but define the size in the respective figure legend.

10) Please also note our reference format: https://link.springer.com/journal/44319/submission-guidelines#cms-Reference-guidelines

11) We updated our journal's competing interests policy in January 2022 and request authors to consider both actual and perceived competing interests. Please name this section 'Disclosure and Competing Interests Statement' and put it after the Acknowledgements section.
See: https://link.springer.com/partners/embo-press/editorial-policies#Competing%20interest%20disclosures

12) We now use CRediT to specify the contributions of each author in the journal submission system. CRediT replaces the author contribution section. Please use the free text box to provide more detailed descriptions and do NOT provide your final manuscript text file with an author contributions section. See also our guide to authors (section 'Author contributions'): https://link.springer.com/journal/44319/submission-guidelines#cms-Revised-submissions

13) All Materials and Methods need to be described in the main text using our 'Structured Methods' format, which is required for all research articles. According to this format, the Methods section should include a Reagents and Tools Table (listing key reagents, experimental models, software, and relevant equipment and including their sources and relevant identifiers), uploaded as separate file, and a Methods section in which we encourage the authors to describe their methods using a step-by-step protocol format with bullet points, to facilitate the adoption of the methodologies across labs. More information on how to adhere to this format as well as downloadable templates (.doc) for the Reagents and Tools Table can be found in our author guidelines (section 'Structured Methods'):

https://link.springer.com/journal/44319/submission-guidelines#cms-Manuscript-organisation-and-formatting

Please add all primer information directly to the Reagents and Tools table.

14) Please order the manuscript sections like this, using only these names:
Title page - Abstract (max. 175 words) - Keywords (max. 5) - Introduction - Results - Discussion - Methods - Data availability section - Acknowledgements (please include here all the funding information) - Disclosure and Competing Interests Statement - References - Figure legends - Expanded View Figure legends

15) Please make sure that all the funding information is also entered into the online submission system and that it is complete and similar to the one in the acknowledgement section of the manuscript text file.

We would also welcome the submission of cover suggestions, or motifs to be used by our Graphics Illustrator in designing a cover.

As part of the EMBO publication's Transparent Editorial Process, EMBO reports publishes online a Review Process File (RPF) to accompany accepted manuscripts. This File will be published in conjunction with your paper and will include the referee reports, your point-by-point response and all pertinent correspondence relating to the manuscript.

You are able to opt out of this by letting the editorial office know (emboreports@embo.org). If you do opt out, the Review Process File link will point to the following statement: "No Review Process File is available with this article, as the authors have chosen not to make the review process public in this case."

I look forward to seeing a revised form of your manuscript when it is ready.

Yours sincerely,

Achim Breiling
Senior Editor
EMBO Reports

Referee #1:

In the current study, Meilin Li et al. investigate host TNF α-mediated cross-species signaling in mosquitoes. The authors demonstrate that host-derived TNF α impairs oocyte formation in the mosquito midgut by suppressing TEP1 expression. Using RNA interference, they further show that TEP1 is regulated by the IMD pathway, and that TNF α inhibits this pathway by interfering with mTAK1 expression. Finally, the authors confirm protein-protein interactions between host-derived TNF α and the mosquito receptors Wengen or Grnd, which are known to regulate TRAF2 and TRAF3 in Drosophila. Overall, the study is comprehensive and addresses an important question. However, there is a major methodological concern:

1. The authors use TNF α−/− mice and compare them directly with wild-type animals, despite the profoundly different and globally reprogrammed immune responses between these two genotypes. The attempt to justify this comparison in Figure 1 and Supplementary Figure 1 is not sufficiently convincing. A more appropriate strategy would be to include TNFR1/2−/− mice as a control group, as TNF α−/− and TNFR1/2−/− mice are expected to exhibit broadly comparable defects in TNF signaling-dependent immune responses. It is also important to note that TNF α deficiency may not directly alter blood-stage Plasmodium yoelii or P. berghei parasitemia in the mammalian host, yet serum from these mice can still differ substantially in cytokine and chemokine composition and thereby variably modulate mosquito immune responses.
2. To determine whether the observed phenotype is specific to TNF α, the authors should perform artificial feeding experiments in which mosquitoes are offered blood and parasite in the presence of defined cytokines, such as TNF α, IFN γ, IL 1β, IL 6, IL 12,etc and then quantify oocyst burden, TEP1 expression, and relevant IMD pathway readouts for each condition.
3. The pull down experiment shown in Figure 5G is not fully convincing. The authors should validate the proposed interactions using additional biophysical approaches such as biolayer interferometry (BLI) or microscale thermophoresis (MST), which provide quantitative binding parameters and orthogonal confirmation. Furthermore, the identity of the second band detected in the His Wengen pull down with anti TNF α needs to be clarified, particularly given that no analogous band is observed in the His

Grnd pull down probed with anti TNF α.

Referee #2:

The manuscript presents compelling data on host-derived TNFα promoting malaria infection in mosquitoes via Wgn/mTRAF3/IMD pathway interactions. However, major concerns include contradictions with prior literature on mosquito TNF signaling, small sample sizes, tissue specificity of RNA-seq data, and lack of mechanistic verification that TNFα reaches the hemolymph and hemocytes. Minor issues mainly relate to figure clarity, gene nomenclature, citation accuracy, and methodological details. Addressing these points will significantly improve the rigor and clarity of the manuscript.

Major Revisions

Contradictions with Previous Studies on Mosquito TNF Signaling
The findings that host-derived TNFα promotes malaria infection in mosquitoes, along with the effects of Wengen (Wgn) or Grnd silencing on parasite loads, contradict prior studies showing that endogenous mosquito TNF signaling (Wgn/Grnd pathway) limits infection (Samantsidis et al., 2025; Srinivasan et al., 2022). The authors should discuss this discrepancy and ideally provide P. berghei infection data when mosquito TNF signaling components (Wgn, Grnd, Eiger) are silenced to clarify the discordance. The species of Plasmodium used should be clearly indicated throughout the text and in figure legends.
Additionally, when discussing Wgn vs Grnd silencing effects on parasite load, the authors should contextualize their results with previous publications and consider whether intrathoracic injections of recombinant TNFα could produce opposite effects.

Sample Size and Statistical Reliability of Ookinete Counts
Counts of ookinetes at 14 h post-feeding (n=10 midguts) are insufficient to draw strong conclusions. A minimum of 30-40 midguts per condition is recommended. Additionally, the 14 h timepoint may be too early; evaluating ookinete numbers at 18 h post-feeding would better capture midgut invasion.
The authors should also comment on the large discrepancy between dsLacZ controls in Figures 5A and 5B, where oocyst numbers differ by approximately four-fold.

Effect of Host-Derived TNFα on Ookinete Transformation
While Fig. S2E shows that TNFα -/- does not influence gametocyte-to-ookinete transformation in vitro, this should be confirmed in vivo.
There is also an apparent inconsistency between Fig. 2C (low ookinete numbers at 14 h post-infection) and Fig. 2D (oocyst numbers exceeding ookinetes). This discrepancy should be addressed.

Mechanistic Verification of TNFα Effects on Mosquito Immunity
It is critical to demonstrate that host-derived TNFα reaches the mosquito hemolymph in sufficient quantities to affect immune signaling and any possible effects on hemocytes. Perfusion and quantification of TNFα would strengthen the mechanistic interpretation.
In parallel, hemocyte populations should be quantified (granulocytes, oenocytoids, prohemocytes) to determine whether feeding on TNFα-/- vs WT mice alters their proportions. Also Monitoring hemocyte attachment to the midgut basal lamina would further clarify how TNFα affects parasite killing.

dsRNA Knockdown Consistency and Controls
Some knockdown experiments (e.g., dsJNK in Fig. 3G) have low and inconsistent replicate numbers. All dsRNA experiments should maintain consistent sample sizes, and dsLacZ controls should be presented on the left of the figure panels, following standard convention, to avoid confusion.

Conflict in TEP1 Expression Data
In Fig. 3D, TEP1 expression appears reduced in TNFα-/- infected groups with Rel1 silencing, which contradicts the text stating no significant changes. The authors should clarify these discrepancies.

Wgn Signaling, Metabolism, and Immunity
Considering that Wgn can act as a metabolic switch in Drosophila, regulating both lipolysis and immunity, the authors should discuss whether host-derived TNFα promotes Plasmodium development primarily by modulating immunity or through changes in resource availability.

Minor Revisions:

Abstract Clarity on TNFα Neutralization
Clarify whether TNFα is neutralized via antibodies, genetic approaches, or other inhibitors, and specify whether this occurs in the host or mosquito.

Introduction and Citations

Correct the citation on line 31 and ensure all references are accurate.

Lines 34-36: Provide additional citations supporting cross-species uptake of host immune effectors; tick studies may be relevant.

Standardize gene symbols (e.g., Wengen vs Wgn) throughout the manuscript and bibliography.

Figure Legends and Clarity

Fig. 1E legend incorrectly states oocyst counts at 2-4 days post-infection; clarify that counts were done at 7 days post-infection.

Fig. 6H: Adjust the scale or explicitly annotate medians to improve visualization.

Gene and Primer Information
Include accession numbers for Wgn (AGAP000728), Grnd (AGAP008399), and other genes. Ensure primers match sequences in VectorBase.

Reference Accuracy
Confirm that "Stone et al., 2016" correctly describes TNFα inhibiting malaria infection in mosquitoes.

Referee #3:

This study uncovers a novel cross-species immune signaling mechanism wherein host-derived TNF-α binds to the mosquito TNF receptor Wengen (Wng), suppressing mosquito immune defenses and enhancing Plasmodium transmission. Mechanistically, TNF-α binding to Wng activates mosquito TRAF3, which suppresses the IMD/Relish pathway via inhibition of TAK1, leading to reduced TEP1 expression and increased parasite survival. Notably, neutralizing TNF-α prevents experimental cerebral malaria and enhances the efficacy of a transmission-blocking vaccine (anti-Pfs25). The findings highlight a surprising pro-parasitic role for host TNF-α and suggest potential for TNF-α-targeted interventions in malaria control.

Major Concerns:

1) Direct Evidence for Receptor Binding: While pull-down data supports Wng-TNF-α interaction, quantitative binding affinity (e.g., ELISA, SPR, or ITC) would strengthen the claim. In lines 338-367, no direct experiment was performed to assess TNF-α binding to Wng, other than support from pull-down and RNAi experiments. Protein-protein interaction experiments are required to confirm that TNF-α directly interacts with the Wng receptor.

2) Specificity of Wng vs. Grnd: Although Wng is implicated, Grnd knockdown also affects parasite load. Further clarification or discussion of their differential roles is needed.

3) Impact on Mosquito Physiology: The long-term effects of TNF-α signaling on mosquito immunity beyond TEP1 were underexplored.

4) Host-Mosquito Interface Dynamics: Temporal/spatial dynamics of TNF-α concentration in the midgut and its stability post-ingestion are not fully addressed.

Minor Concerns:
1) The potential systemic effects of TNF-α on mosquito hemocyte populations or stem cells should be briefly discussed.
2) Some figure legends are overly verbose and could be more concise.
3) In lines 154-167, the term "symbiotic microbiota" is inaccurate. The mosquito midgut microbiota includes mutualists, commensals, and potential pathogens, not all of which are symbiotic.
4) Clarify whether APL1A or APL1C is analyzed in line 191 and Figure 2G, as each isoform has specificity for rodent or human malaria parasites.
5) Grammar & Language: Generally clear and well-written with minor grammatical issues:
a) Inconsistent spacing around hyphens (e.g., "TNF-α-/-" should be consistently spaced).
b) Occasional redundant phrasing (e.g., "mosquito feeding on the infected-WT or TNF-α-/- mice").
c) Some passive constructions could be rephrased for clarity.
Typos
a) "TEP1 activation through attenuating..." → "by attenuating"
b) "difference infection intensity" → "difference in infection intensity"
c) "difference of infection intensity" → "difference in infection intensity"

Suggested Additional Experiments (must be done to address major concerns):

1) Biophysical characterization: Perform ELISA or surface plasmon resonance (SPR) to quantify TNF-α binding affinity for Wng vs. Grnd.
2) Temporal dynamics: Analyze TNF-α levels and downstream signaling markers at multiple timepoints post-bloodmeal.
3) Transcriptomic profiling: Conduct RNA-seq on hemocytes post-TNF-α exposure to identify other affected immune genes.
4) Clarify the isoform-specific roles of APL1A versus APL1C in defending against rodent versus human malaria parasites to better delineate the mechanisms underlying cross-species immune regulation. Additionally, while the IMD pathway has been specifically characterized as a defense mechanism against human malaria parasites in Anopheles mosquitoes, the manuscript would benefit from a more granular discussion of how this pathway may differentially influence immunity against rodent versus human malaria species, and any observed or expected discrepancies.

Dear editor and reviewers,

Thanks a lot for your comments, which would greatly improve our manuscript. We have responded to your comments point-by-point as following. We hope you will be satisfied with our responses.

Responses to Referee #1's comments

In the current study, Meilin Li et al. investigate host TNF α-mediated cross-species signaling in mosquitoes. The authors demonstrate that host-derived TNF α impairs oocyte formation in the mosquito midgut by suppressing TEP1 expression. Using RNA interference, they further show that TEP1 is regulated by the IMD pathway, and that TNF α inhibits this pathway by interfering with mTAK1 expression. Finally, the authors confirm protein-protein interactions between host-derived TNF α and the mosquito receptors Wengen or Grnd, which are known to regulate TRAF2 and TRAF3 in Drosophila. Overall, the study is comprehensive and addresses an important question. However, there is a major methodological concern:

Q1. The authors use TNF $\alpha^{-/-}$ mice and compare them directly with wild-type animals, despite the profoundly different and globally reprogrammed immune responses between these two genotypes. The attempt to justify this comparison in Figure 1 and Supplementary Figure 1 is not sufficiently convincing. A more appropriate strategy would be to include TNFR1/$2^{-/-}$ mice as a control group, as TNF $\alpha^{-/-}$ and TNFR1/$2^{-/-}$ mice are expected to exhibit broadly comparable defects in TNF signaling-dependent immune responses. It is also important to note that TNF α deficiency may not directly alter blood-stage *P. yoelii* or *P. berghei* parasitemia in the mammalian host, yet serum from these mice can still differ substantially in cytokine and chemokine composition and thereby variably modulate mosquito immune responses.

Response: Thanks for your appreciation of our study and providing us with insightful comments. The control mice we used alongside TNF-$\alpha^{-/-}$ mice were littermate controls, which greatly minimized the background difference between those two mice. For clarity, we have replaced the WT with "littermate control" or "control" throughout our manuscript. Furthermore, we validated the findings from TNF-$\alpha^{-/-}$ mice using a TNF-α neutralizing antibody. Importantly, neither TNF-α deficiency nor neutralization significantly affected *Plasmodium* parasitemia in the mammalian host, for either *P. yoelii* or *P. berghei* (Fig EV1A, B, D, E). The relevant description is provided in Lines 97, and we have replaced the term "growth" with "parasitemia" for improved precision.

While including TNFR1/$2^{-/-}$ mice would have strengthened our control group, commercially available TNFR1/$2^{-/-}$ mice are not accessible in China. We therefore performed alternative experiments using anti-TNFR1 and anti-TNFR2 blocking antibodies to examine their effects on parasite infection in mosquitoes.

Consequently, no significant changes in malaria parasite transmission were observed in mosquitoes fed on infected mice pretreated with both anti-TNFR1 and anti-TNFR2 antibodies.

Q2. To determine whether the observed phenotype is specific to TNF α, the authors should perform artificial feeding experiments in which mosquitoes are offered blood and parasite in the presence of defined cytokines, such as TNF α, IFN γ, IL 1β, IL 6, IL 12, etc and then quantify oocyst burden, TEP1 expression, and relevant IMD pathway readouts for each condition.

Response: Thanks for your insightful suggestion, which would significantly improve the rigor of our findings. We have observed the influence of several cytokines on malaria transmission, and

found the depletion of TNF α, IFN γ, or IL-6 could significantly modulate malaria transmission. Therefore, we compared the effect of the supplement of either rIFN, rTNF-a or rIL-6 on parasite infection, TEP1 expression and IMD pathway in mosquitoes. As a result, we found both the supplement of IFN and TNF-a, but not IL-6, could enhance parasite infection in mosquitoes. Notably. only the supplement of TNF significantly down regulated the expression of both TEP1 and REL2. Thus, our data strongly supported the specific role of TNF α in promoting malaria transmission via suppression of IMD pathway.

Q3. The pull down experiment shown in Figure 5G is not fully convincing. The authors should validate the proposed interactions using additional biophysical approaches such as biolayer interferometry (BLI) or microscale thermophoresis (MST), which provide quantitative binding parameters and orthogonal confirmation. Furthermore, the identity of the second band detected in the His Wengen pull down with anti TNF α needs to be clarified, particularly given that no analogous band is observed in the His Grnd pull down probed with anti TNF α.

Response: Thanks. Based on instrument availability at our university, we used ELISA and SPR—rather than BLI and MST—to quantify the binding between TNF and Wengen. Both assays revealed strong binding affinity between TNF and Wengen, whereas no significant binding signal was observed between TNF and Grnd. These results are presented in Fig. 5G–H, and the corresponding description has been added to the text (Lines 276–277).

Regarding the second band detected by anti-TNF-α in the His-Wengen pull-down assay, it likely arose from high TNF loading; similar observations have been reported previously (Samivel *et al*, 2020).

Responses to Referee #2's comments：

Q1： The manuscript presents compelling data on host-derived TNFα promoting malaria infection in mosquitoes via Wgn/mTRAF3/IMD pathway interactions. However, major concerns include contradictions with prior literature on mosquito TNF signaling, small sample sizes, tissue specificity of RNA-seq data, and lack of mechanistic verification that TNFα reaches the hemolymph and hemocytes. Minor issues mainly relate to figure clarity, gene nomenclature, citation accuracy, and methodological details. Addressing these points will significantly improve the rigor and clarity of the manuscript.

Response: Thanks for your comments, which will significantly improve the rigor and clarity of our manuscript. We have responded to your comments point-by-point as following.

Major Revisions

Q2： Contradictions with Previous Studies on Mosquito TNF Signaling

The findings that host-derived TNFα promotes malaria infection in mosquitoes, along with the effects of Wengen (Wgn) or Grnd silencing on parasite loads, contradict prior studies showing that endogenous mosquito TNF signaling (Wgn/Grnd pathway) limits infection (Samantsidis et al., 2025; Srinivasan et al., 2022). The authors should discuss this discrepancy and ideally provide *P. berghei* infection data when mosquito TNF signaling components (Wgn, Grnd, Eiger) are silenced to clarify the discordance. The species of *Plasmodium* used should be clearly indicated throughout the text and in figure legends.

Response: Thanks for your concerning. Actually, we discussed the contradictory of our results to

previous studies, but did not explain the possible mechanism behind this. According to your suggestion, we confirmed that the silence of either Wgn or Grnd resulted in the significant reduction of *P. berghei* infection in mosquitoes, which is also contradictory to what is found in Samantsidis et al., 2025. Now, we think the discrepancy could be interpreted as the different doses, but not the different parasite species, used in our and previous study. We found that TNFα has a significantly higher affinity for binding with Wng compared to Grnd, indicating that TNFα can only bind to and activate Wng at physiological concentrations. This finding aligns with our study, which highlights the dependence of Wng in TNFα's pro-transmission effect on malaria. In contrast, at sufficiently high levels, TNFα activates both the high-affinity receptor Wng and the low-affinity receptor Grnd, which was supported by previous observation of the involvement of both Wng and Grnd in the inhibition of malaria transmission at elevated concentrations of TNF. Since the activation of Wng and Grnd by Eiger in *Drosophila* is known to promote distinct cellular functions, it is plausible that high concentrations of TNF might elicit responses that might contradict those induced by lower concentrations. This suggests a dual effect of dose-dependent TNFα on the mosquito immune response. Interestingly, a similar effect has been reported regarding TNFα's influence on the growth of mammalian cells. It has been reported TNF-α at low concentration promotes, but at high concentration inhibit, both tumor and neuron cells(Tang et al, 2013; Mahdavi Sharif et al, 2020) However, the underlying mechanism is still unknown. Additionally, P. yoelii-infection model was utilized to explore the underlying mechanism of TNF-promoted malaria transmission in our study. Therefore, we have added a sentence of "To explore the underlying mechanisms of TNF-promoted malaria transmission, we utilized a *P. yoelii* infection model in the following sections, unless a different species is specified." in line 122-123. In addition, the species of *Plasmodium* used has been indicated throughout the text and in figure legends to make it clearer. Please refer to our revised manuscript for details.

Q3: Additionally, when discussing Wgn vs Grnd silencing effects on parasite load, the authors should contextualize their results with previous publications and consider whether intrathoracic injections of recombinant TNFα could produce opposite effects.

Response: Thanks. It has been discussed as above. We proposed that the concentration, but not the intrathoracic injections, produce the opposite effects in prior study.

Q4: Sample Size and Statistical Reliability of Ookinete Counts

Counts of ookinetes at 14 h post-feeding (n=10 midguts) are insufficient to draw strong conclusions. A minimum of 30-40 midguts per condition is recommended. Additionally, the 14 h timepoint may be too early; evaluating ookinete numbers at 18 h post-feeding would better capture midgut invasion.

Response: Thanks for your suggestion. According to your suggestion, we have increased the midguts for calculation of ookinetes to 47, and found there is still no significant difference between mosquitoes fed on the infected WT and TNF-deficient mice. The result has been updated in Fig.2C. As we aimed to evaluate the effect of TNF-deficiency the generation of ookinetes in mosquito midgut, the 14 h timepoint we chose should be more appropriate.

Q5: The authors should also comment on the large discrepancy between dsLacZ controls in Figures 5A and 5B, where oocyst numbers differ by approximately four-fold.

Response: Thanks. The experiment of Figures 5A and 5B was performed independently. We observed the oocyst number can fluctuate across separate experiment, due to variations in both the parasitemia and mosquito physiological status. To ensure more consistence dsLacZ controls, we have repeated these experiments and updated the manuscript with the new results. Please refer to

our revised manuscript for details.

Q6: Effect of Host-Derived TNFα on Ookinete Transformation

While Fig. S2E shows that TNFα -/- does not influence gametocyte-to-ookinete transformation in vitro, this should be confirmed in vivo.

Response: Thanks for your suggestion. Actually, the *in vivo* effect of $TNF\alpha^{-/-}$ on gametocyte-to-ookinete transformation has been performed as in Figure2A-C. In this experiment, we did not find significant difference of ookinetes generated in mosquitoes feeding on WT or $TNF\alpha^{-/-}$ infected mice.

Q7: There is also an apparent inconsistency between Fig. 2C (low ookinete numbers at 14 h post-infection) and Fig. 2D (oocyst numbers exceeding ookinetes). This discrepancy should be addressed.

Response: Sorry for the confusion. Counting all ookinetes in the midgut is technically challenging, so we instead quantified the average number of ookinetes per 1000 red blood cells, similar to how we measured mouse parasitemia. Because the number of ookinetes per 1000 RBCs was low, we present these values as relative numbers. In Fig. 2C, the relative number of ookinetes at 14 hours post-blood meal is expressed as the ratio of the average ookinete count in mosquitoes fed on TNF-$\alpha^{-/-}$ or control mice relative to that in mosquitoes fed on wild-type control mice. This differs from the absolute oocyst counts shown in Fig. 2D. To improve clarity, we have revised the corresponding figure legends. In addition, as noted in our earlier response, we have increased the sample size to 47 mosquitoes per group.

Q8: Mechanistic Verification of TNFα Effects on Mosquito Immunity

It is critical to demonstrate that host-derived TNFα reaches the mosquito hemolymph in sufficient quantities to affect immune signaling and any possible effects on hemocytes. Perfusion and quantification of TNFα would strengthen the mechanistic interpretation.

Response: Thanks. Now, we have quantified the TNF in hemolymph of mosquitoes after they fed on the infected WT mice. As a result, we detected a significant level of TNF in hemolymph of mosquitoes TNF at 24 h post blood feeding (Fig.5J in the revised manuscript). Along with the binding of TNF to hemocytes in our immunofluorescent assay, this result further supported the possible effect of host-derived TNF on hemocytes of mosquitoes.

Q9: In parallel, hemocyte populations should be quantified (granulocytes, oenocytoids, prohemocytes) to determine whether feeding on TNFα-/- vs WT mice alters their proportions. Also Monitoring hemocyte attachment to the midgut basal lamina would further clarify how TNFα affects parasite killing.

Response: Thanks. Now, we have evaluated the effect of TNF-deficiency on the hemocyte populations. As compared to the mosquitoes feeding on the infected WT mice, we also observed a significantly increased proportion of granulocytes and oenocytoids, accompanied by a decreased proportion of prohemocytes, in hemolymph of mosquitoes feeding on the infected TNFα-/- mice. This new data has presented as Fig.5N in the revised manuscript. This result indicates that TNFα signaling through Wgn significantly inhibited the differentiation of prohemocytes into granulocytes and oenocytoids. Our initial immunofluorescence assay did not detect Wng-mediated binding of host TNF on prohemocytes, which appeared inconsistent with this observation. We therefore repeated the immunofluorescence assay and detected a clear TNF/Wng signal on prohemocytes, which is now shown in Fig. 5K. Based on these findings, we proposed that TNF/Wng might not only impede the differentiation of prohemocytes into granulocytes and oenocytoids, but also suppress the activation of IMD signaling in granulocytes. Additionally, the hemocytes attachment to the midgut basal lamina has been performed. We found more hemocytes were attached to the

midgut basal lamina in mosquitoes feeding on the infected TNFα$^{-/-}$ mice than that feeding on the infected control mice, the data has been presented as (Fig. 5O-P). The context in both Result (line 279-298) section has been revised accordingly. Please refer to our revised manuscript.

Q10: dsRNA Knockdown Consistency and Controls

Some knockdown experiments (e.g., dsJNK in Fig. 3G) have low and inconsistent replicate numbers. All dsRNA experiments should maintain consistent sample sizes, and dsLacZ controls should be presented on the left of the figure panels, following standard convention, to avoid confusion.

Response: Thanks for your suggestion. Now, dsLacZ controls in all Figures have been presented on the left of the figure panels to make it clearer.

Q11: Conflict in TEP1 Expression Data

In Fig. 3D, TEP1 expression appears reduced in TNFα-/- infected groups with Rel1 silencing, which contradicts the text stating no significant changes. The authors should clarify these discrepancies.

Response: Sorry for the confusion of our description. Now, the sentences have been revised as following: " Similarly, no significant changes of difference in TEP1 expression or oocyst counts were observed between mosquitoes fed on infected control and TNF-α$^{-/-}$ mice (Fig. 3D-E), after knocking down REL1 (line 204-206)" Hopefully, we have made it clearer now.

Q12: Wgn Signaling, Metabolism, and Immunity

Considering that Wgn can act as a metabolic switch in Drosophila, regulating both lipolysis and immunity, the authors should discuss whether host-derived TNFα promotes *Plasmodium* development primarily by modulating immunity or through changes in resource availability.

Response: Thanks for your insightful suggestion. We proposed that host-derived TNFα promotes *Plasmodium* development primarily by modulating immunity, but its role in the regulation of mosquito lipolysis could not be completely excluded. This has been incorporated in the Discussion section (line 395-397).

Minor Revisions:

Q13: Abstract Clarity on TNFα Neutralization

Clarify whether TNFα is neutralized via antibodies, genetic approaches, or other inhibitors, and specify whether this occurs in the host or mosquito.

Response: Thanks. It has been revised as "the neutralization of host-derived TNF by anti-TNF antibody".

Q14: Introduction and Citations

Correct the citation on line 31 and ensure all references are accurate.

Response: Thanks. The citation format has been corrected.

Q15: Lines 34-36: Provide additional citations supporting cross-species uptake of host immune effectors; tick studies may be relevant.

Response: Thanks. Additional citation about tick studies has been provided.

Q16: Standardize gene symbols (e.g., Wengen vs Wgn) throughout the manuscript and bibliography.

Response: Thanks. All gene symbols have been standardized throughout the manuscript and bibliography.

Q17: Figure Legends and Clarity

Fig. 1E legend incorrectly states oocyst counts at 2-4 days post-infection; clarify that counts were done at 7 days post-infection.

Response: Thanks. It has been corrected.

Q18: Fig. 6H: Adjust the scale or explicitly annotate medians to improve visualization.

Response: Thanks. It has been corrected.

Q19: Gene and Primer Information

Include accession numbers for Wgn (AGAP000728), Grnd (AGAP008399), and other genes. Ensure primers match sequences in VectorBase.

Response: Thanks. The accession numbers for the new genes in *An. stephensi* have been added.

Q20: Reference Accuracy

Confirm that "Stone et al., 2016" correctly describes TNFα inhibiting malaria infection in mosquitoes.

Response: Thanks. It has been corrected.

Referee #3:

This study uncovers a novel cross-species immune signaling mechanism wherein host-derived TNF-α binds to the mosquito TNF receptor Wengen (Wng), suppressing mosquito immune defenses and enhancing *Plasmodium* transmission. Mechanistically, TNF-α binding to Wng activates mosquito TRAF3, which suppresses the IMD/Relish pathway via inhibition of TAK1, leading to reduced TEP1 expression and increased parasite survival. Notably, neutralizing TNF-α prevents experimental cerebral malaria and enhances the efficacy of a transmission-blocking vaccine (anti-Pfs25). The findings highlight a surprising pro-parasitic role for host TNF-α and suggest potential for TNF-α-targeted interventions in malaria control.

Response: Thanks a lot for your appreciation of our study.

Major Concerns:

1) Direct Evidence for Receptor Binding: While pull-down data supports Wng-TNF-α interaction, quantitative binding affinity (e.g., ELISA, SPR, or ITC) would strengthen the claim. In lines 338-367, no direct experiment was performed to assess TNF-α binding to Wng, other than support from pull-down and RNAi experiments. Protein-protein interaction experiments are required to confirm that TNF-α directly interacts with the Wng receptor.

Response: Thanks. It has been corrected. As our above response to Reviewer 1's comments, we have used both ELISA and SPR assay to confirm the direct interaction between TNF and Wengen.

2) Specificity of Wng vs. Grnd: Although Wng is implicated, Grnd knockdown also affects parasite load. Further clarification or discussion of their differential roles is needed.

Response: Thanks for your suggestion. We have now added a discussion on the roles of Wng and Grnd in mosquito anti-malaria immune responses as follows: Both Wng and Grnd may participate in Eiger-mediated mosquito immunity against *Plasmodium* infection, whereas the *Plasmodium* transmission-promoting effect of host-derived TNF relies exclusively on Wng.

3) Impact on Mosquito Physiology: The long-term effects of TNF-α signaling on mosquito immunity beyond TEP1 were underexplored.

Response: Thanks. for your suggestion. Actually, we have evaluated the effect of TNF-deficiency on mosquito physiology, such as attraction, blood taken, fitness, and production, the data has been presented in Figure EV2. In addition, we have also evaluated TNF-deficiency on hemocytes populations, as our earlier response to reviewer 1' comments.

4) Host-Mosquito Interface Dynamics: Temporal/spatial dynamics of TNF-α concentration in the midgut and its stability post-ingestion are not fully addressed.

Response: Thanks for your valuable suggestion. In fact, we were surprised to find that a high concentration of TNF-α persisted in mosquito midgut. This observation promoted us to assess other cytokines, such as IL-12p70, IL-6 and in midgut of mosquito fed on the infected mice. However, we also detected that these cytokines similarly persist at high concentrations in mosquito midgut. Upon reviewing the literature, we found that hemoglobin can interfere the detection of TNF by ELISA (Hemoglobin interference with an enzyme-linked immunosorbent assay for the detection of tumor necrosis factor-alpha(Simoni et al, 1995). Therefore, we suspect that the detection of TNF-α in mosquito midgut may be a false-positive result, potentially interfered by hemoglobin released from red blood cells hemolysis in mosquito midgut. Consequently, we have removed this data from our manuscript. The removal of this result will not affect our conclusion about the entry of TNF in mosquito midgut during blood feeding, as we have successfully detected the TNF in hemolymph and hemocyte by immunofluorescence assay and ELISA.

Figure for referee with unpublished data has been removed upon request by the authors.

Minor Concerns:

1) The potential systemic effects of TNF-α on mosquito hemocyte populations or stem cells should be briefly discussed.

Response: Thanks for your suggestion. As described in our earlier response to reviewer 1, the effect of TNF-deficiency on mosquito hemocyte populations has been determined.

2) Some figure legends are overly verbose and could be more concise.

Response: Thanks. We have carefully checked all figure legends and made it more concise.

3) In lines 154-167, the term "symbiotic microbiota" is inaccurate. The mosquito midgut microbiota includes mutualists, commensals, and potential pathogens, not all of which are symbiotic.

Response: Thanks for your suggestion. The term "symbiotic microbiota" has been corrected into " microbiota".

4) Clarify whether APL1A or APL1C is analyzed in line 191 and Figure 2G, as each isoform has specificity for rodent or human malaria parasites.

Response: Thanks for your suggestion. We have carefully checked the relevant section and confirmed that the gene should indeed be APL1. As previously reported, only a single *APL1* gene is present in *An. stephensi* (Mitri et al, 2020). In other words, *An. stephensi* APL1 has no isoforms, in contrast to the multiple isoforms found in *An. gambiae*.

5) Grammar & Language: Generally clear and well-written with minor grammatical issues:

a) Inconsistent spacing around hyphens (e.g., " TNF-$\alpha^{-/-}$" should be consistently spaced).

b) Occasional redundant phrasing (e.g., "mosquito feeding on the infected-WT or TNF-$\alpha^{-/-}$ mice").

c) Some passive constructions could be rephrased for clarity.

Response: Thanks for your suggestion. Now, we have carefully read through our manuscript and corrected the following errors, such as the inconsistent spacing around hyphens, redundant phrasing and passive construction.

Typos

a) "TEP1 activation through attenuating..." → "by attenuating"

Response: It has been corrected.

b) "difference infection intensity" → "difference in infection intensity"

Response: It has been corrected.

c) "difference of infection intensity" → "difference in infection intensity"

Response: It has been corrected.

Suggested Additional Experiments (must be done to address major concerns):

1) Biophysical characterization: Perform ELISA or surface plasmon resonance (SPR) to quantify TNF-α binding affinity for Wng vs. Grnd.

Response: Thanks for your suggestion. As described in our earlier response to the reviewer's comments, we have used both ELISA and SPR assays to verify the direct interaction between TNF and Wengen. These results are presented in Fig. 5G and Fig. 5H, and the corresponding text in the Results section has been revised accordingly (Lines 276–278).

2) Temporal dynamics: Analyze TNF-α levels and downstream signaling markers at multiple timepoints post-bloodmeal.

Response: Thanks for your suggestion. We have now dynamically examined the expression of *REL2* in mosquitoes following TNF-α exposure. We found that REL2 expression was significantly downregulated at 12 and 24 h, then upregulated at 48 h, and finally returned to baseline levels at 72 h. Since we were unable to identify a suitable location to integrate this result into the main manuscript, we have chosen to present these data here in our response. Please see the figure below.

Figure for referee with unpublished data has been removed upon request by the authors.

The dynamic expression of *REL2* in mosquitoes feeding on the infected mice

3) Transcriptomic profiling: Conduct RNA-seq on hemocytes post-TNF-α exposure to identify other affected immune genes.

Response: Thanks. We previously performed RNA-seq on whole mosquitoes following TNF-α exposure, but were unable to conduct hemocyte-specific RNA-seq due to the difficulty of collecting sufficient highly pure hemocytes. Instead, we used quantitative real-time PCR to examine

expression changes of a panel of immune-related genes in mosquito hemocytes after TNF treatment, including *Defensin*, *Cecropin-A*, *HPX*, *NOS*, *STAT-A*, *REL2*, and *REL1*. Among these, only *STAT-A*, *REL2*, and *REL1* were significantly upregulated. We have demonstrated that neither STAT-A nor REL1 contributed to the enhanced malaria transmission mediated by TNF (Fig. 3C–E). These results are presented separately in Figure EV3F and Figure EV4B, and the corresponding description has been revised in the Results section (Lines 172–175 and 194–198). We hope these revisions address your concern.

4) Clarify the isoform-specific roles of APL1A versus APL1C in defending against rodent versus human malaria parasites to better delineate the mechanisms underlying cross-species immune regulation. Additionally, while the IMD pathway has been specifically characterized as a defense mechanism against human malaria parasites in Anopheles mosquitoes, the manuscript would benefit from a more granular discussion of how this pathway may differentially influence immunity against rodent versus human malaria species, and any observed or expected discrepancies.

Response: Thanks for your valuable comment. It is well established that *An. gambiae* exhibits isoform-specific roles of APL1A versus APL1C of in defending against rodent versus human malaria parasites, respectively. However, unlike *An. gambiae*, which carries three APL1 isoforms in, only a single *APL1* ortholog has been identified in *An. stephensi* (Mitri et al, 2020). Additionally, the role of the IMD pathway in *An. gambiae* responses to rodent malaria infection appears contradictory. In this study, we found the essential role of IMD pathway in *An. stephensi* against rodent malaria infection, which is consistent with a prior study(Feng et al, 2021). This indicates the immune responses of *An. stephensi* against malaria parasites differs from that of *An. gambiae,* and results should be interpreted with caution across different parasite-mosquito combinations.

Reference

Feng Y, Chen L, Gao L, Dong L, Wen H, Song X, Luo F, Cheng G & Wang J (2021) Rapamycin inhibits pathogen transmission in mosquitoes by promoting immune activation. PLOS Pathog 17: e1009353

Mahdavi Sharif P, Jabbari P, Razi S, Keshavarz-Fathi M & Rezaei N (2020) Importance of TNF-alpha and its alterations in the development of cancers. Cytokine 130: 155066

Mitri C, Bischoff E, Eiglmeier K, Holm I, Dieme C, Brito-Fravallo E, Raz A, Zakeri S, Nejad MIK, Djadid ND, et al (2020) Gene copy number and function of the APL1 immune factor changed during Anopheles evolution. Parasites & Vectors 13: 18

Samivel R, Nagarajan RP, Subramanian U, Khan AA, Masmali A, Almubrad T & Akhtar S (2020) Inhibitory effect of ursolic acid on ultraviolet B radiation-induced oxidative stress and proinflammatory response-mediated senescence in human skin dermal fibroblasts. Oxid Med Cell Longevity 2020: 1–17

Simoni J, Simoni G, Lox CD, Prien SD & Shires GT (1995) Hemoglobin interference with an enzyme-linked immunosorbent assay for the detection of tumor necrosis factor-alpha. Anal Chim Acta 313: 1–14

Tang X, Wang Y, Zhou S, Qian T & Gu X (2013) Signaling pathways regulating dose-dependent dual effects of TNF-α on primary cultured schwann cells. Mol Cell Biochem 378: 237–246

Dear Prof. Xu,

Thank you for the submission of your revised manuscript to our editorial offices. I have already forwarded to you the reports I received from the three referees that were asked to re-evaluate the study, you will also find below again. As you know, referees #1 and #3 now basically support publication of the revised manuscript in EMBO reports. In contrast, referee #2 indicates that s/he remains critical, stating that s/he is not convinced that the mechanistic conclusions proposed by the authors are sufficiently supported by the presented data. In particular, the referee states that several central claims regarding IMD signaling, hemocyte differentiation, and interpretation of pathway-specific effects remain largely inferential and, in some cases, inconsistent with the presented figures or prior literature.

Going through the p-b-p-response you have provided (further revision plan), and after receiving feedback on the report of referee #2 by referee #3, I feel that the remaining concerns of the referees can be addressed during a further revision. I thus invite you to revise you manuscript further and to provide a final detailed point-by-point response, as indicated in your revision plan and as outlined by referee #3 in his/her feedback. Please find his/her further comment also below.

Moreover, I have the editorial requests below I ask you to also address in the final revised manuscript. Please also provide a final p-b-p-response to the editorial requests.

Editorial requests:

- Please order the manuscript sections like this, using only these names:
Title page - Abstract - Keywords - Introduction - Results - Discussion - Methods - Data availability section - Acknowledgements - Disclosure and Competing Interests Statement - References - Figure legends - Expanded View Figure legends

- We now use CRediT to specify the contributions of each author in the journal submission system. CRediT replaces the author contribution section. Please use the free text box to provide more detailed descriptions and do NOT provide your final manuscript text file with an author contributions section. See also our guide to authors (section 'Author contributions'): https://link.springer.com/journal/44319/submission-guidelines#cms-Revised-submissions

- In the 'Data Availability' section, please add a direct link to the dataset.

- Please check again that the number "n" for how many independent experiments were performed, their nature (biological versus technical replicates), the bars and error bars (e.g. SEM, SD) and the test used to calculate p-values is indicated in the respective figure legends (main and EV figures). Please also check that all the p-values are explained in the legend, and that these fit to those shown in the figure. Please provide statistical testing where applicable. Please avoid the phrase 'independent experiment' but clearly state if these were biological or technical replicates. Please also indicate (e.g. with n.s.) if testing was performed, but the differences are not significant. In case n=2, please show the data as separate datapoints without error bars and statistics. See also:

https://link.springer.com/journal/44319/submission-guidelines#cms-Figure-and-data-presentation

If n<5, please show single datapoints for diagrams. Moreover:
- Please note that the exact p values are not provided in the legends of figures 1B-I; 2D, F-H, J, K; 3B, C, D, E, F, G, H, I; J, K, L, M; 4A, B, C, D, H, I, J-M; 5A-E, J, L, M, O; 6A, C, D, E-K; 7A-D; EV3 A, C, D; EV4 A-K; EV5 A, B, D; EV7 A-D.
- Please note that the box plots need to be defined in terms of minima, maxima, centre, bounds of box and whiskers, and percentile in the legends of figure EV2 A.

- Please move the primer and peptide information (Tables EV1 and EV2) to the reagents and tools table and remove the tables from the manuscript. Please update the callouts and also add further callouts to the Reagents & Tools Table where appropriate. Please also remove the example table from the final Reagents & Tools table.

- Please add scale bars of similar style and thickness to microscopic images, using clearly visible black or white bars (depending on the background). Please place these in the lower right corner of the images themselves. Please do not write on or near the bars in the image but define the size in the respective figure legend. Presently, most scale bars are too thin and have text nearby.

- Thanks for providing the source data. Please update this (and the source data checklist) in case new experiments are provided during revision and upload the final source data as one ZIPed folder per figure, containing all the source data for this figure.

In addition, I would need from you uploaded separately:
- a short, two-sentence summary of the manuscript (not more than 35 words).
- two to four short (!) bullet points highlighting the key findings of your study (two lines each).

- a schematic summary figure as separate file that provides a sketch of the major findings (not a data image) in jpeg or tiff format (with the exact width of 550 pixels and the exact height of 300 pixels) that can be used as a visual synopsis on our website. Please upload this separately and remove the graphical abstract from the manuscript text file.

I look forward to seeing the further revised version of your manuscript when it is ready. Please let me know if you have questions regarding the revision.

We would also welcome the submission of cover suggestions, or motifs to be used by our Graphics Illustrator in designing a cover.

As part of the EMBO publication's Transparent Editorial Process, EMBO reports publishes online a Review Process File (RPF) to accompany accepted manuscripts. This File will be published in conjunction with your paper and will include the referee reports, your point-by-point response and all pertinent correspondence relating to the manuscript.

You are able to opt out of this by letting the editorial office know (emboreports@embo.org). If you do opt out, the Review Process File link will point to the following statement: "No Review Process File is available with this article, as the authors have chosen not to make the review process public in this case."

I look forward to seeing a further revised form of your manuscript when it is ready.

Yours sincerely,

Achim Breiling
Senior Editor
EMBO Reports

Referee #1:

Thank you for the responses.

Referee #2:

I appreciate the authors' efforts to address my previous comments. Although, the revised manuscript contains several interesting observations regarding the effect of host-derived TNF-α on mosquito susceptibility to Plasmodium infection, I still remain unconvinced that the mechanistic conclusions proposed by the authors are sufficiently supported by the presented data. In particular, several central claims regarding IMD signaling, hemocyte differentiation, and interpretation of pathway-specific effects remain largely inferential and, in some cases, inconsistent with the presented figures or prior literature. Given the EMBO reports' policy allowing only a single round of revisions, I am in the unfortunate position of having to reject the manuscript in its current form, for the following reasons:

Evidence supporting IMD-dependent regulation of TEP1 remains insufficient

1.The authors propose that host-derived TNF-α suppresses mosquito anti-Plasmodium immunity through inhibition of the IMD pathway, ultimately reducing TEP1 expression. Although the authors demonstrated the reduction of TEP1 active form following infection on TNF-α depleted mosquitoes, the evidence supporting the mechanistic link between TNF-α and IMD remains incomplete.
The manuscript does not directly demonstrate IMD pathway activation or suppression. In particular, no evidence is provided regarding:
REL2 cleavage/activation or,
REL2 nuclear translocation
The conclusions are therefore based primarily on transcriptional changes and RNAi phenotypes, which are insufficient to establish direct suppression of IMD signaling.

2.Ookinete transformation efficiency was not evaluated in vivo as requested
The authors state in their response that the in vivo effect of TNF-α deficiency on ookinete development was already addressed in Figure 2A-C by quantifying the ookinete counts in the mosquito midguts or the expression levels of ookinete-specific genes in mosquitoes fed on WT or TNF-α-/- mice.
However, this is not equivalent to the experiment that was requested.
To properly assess in vivo transformation efficiency, mosquito midguts should be dissected approximately 16-18 h post blood feeding and the proportions of zygotes, retorts, and mature ookinetes within each blood bolus should be quantified relative to

total parasite numbers. This would allow proper evaluation of whether TNF-α deficiency affects parasite developmental progression within the mosquito midgut.
The absence of significant differences in invaded ookinete numbers is partially reassuring; however, it does not fully exclude effects on parasite developmental dynamics prior to invasion.

3.Potential systemic confounding effects in TNF-α-deficient mice remain insufficiently addressed
Deletion of TNF-α is known to induce broad immunological and metabolic alterations in vertebrates. Therefore, the observed mosquito phenotypes may not solely reflect direct effects of TNF-α itself.
In particular, TNF-α deficiency during Plasmodium infection could alter the insulin signaling, a point that is especially relevant given previous studies linking vertebrate insulin signaling to increased permissiveness of Plasmodium infection in Anopheles stephensi. These potential systemic confounders should had been discussed more carefully. In this right, the authors mentioned in the discussion that "ingested human insulin was found to reduce P. falciparum burden...", which is actually the opposite of what Pakpour et al., 2012 demonstrated.

4.Hemocyte characterization and differentiation claims remain unconvincing
The images presented in Figure 5K do not convincingly resemble canonical granulocytes. The cells appear substantially larger (>40 μm) than expected granulocytes and contain abundant lipid droplets. These cells may instead represent fat body contaminants or potentially megacytes rather than granulocytes.
Furthermore, the statement:
"host-derived TNF-α impedes the differentiation of prohemocytes into granulocytes and oenocytoids"
is not sufficiently supported by the presented data (Fig. 5N).
The authors are heavily relying on merely the proportions of different hemocyte subpopulations, therefore, the conclusions regarding hemocyte differentiation should be substantially toned down.

5.The explanation for discrepancies with previous studies remains insufficient
The authors propose that discrepancies between the current study and previous publications can be explained primarily by differences in TNF-α dose.
However, this explanation remains insufficient, particularly because contradictory phenotypes are observed following silencing of the same genes (Wgn and Grnd) across studies. It remains unclear how differences in ligand concentration alone would explain opposite phenotypic outcomes resulting from receptor silencing experiments.
Additionally, the manuscript itself reports discrepancies with previous observations regarding STAT-A-dependent effects on parasite development. In the text the authors stated: " although it significantly increased the oocyst number in mosquitoes feeding on the infected control mice (Fig. 3C)". Gupta et al., showed that STAT-A silencing increases the numbers of P.berghei oocysts.

It appears as though the particular work has a lot of discrepancies with the current literature, which could be the result of the mosquito species or strain used.

Finally, the authors did not address my previous suggestion regarding intrathoracic injection of recombinant TNF-α to directly test their proposed dose-dependent model in vivo. Such experiments would substantially strengthen the proposed explanation for the apparent discrepancies with previous studies.
Minor comments
1.The citation "B et al., 1996" should be corrected to Lemaitre et al., 1996 (PMID: 8808632).
2.Please carefully verify citation formatting throughout the manuscript according to EMBO reports standards.
3.Wengen nomenclature remains inconsistent throughout the manuscript and should be standardized.

Referee #3:

The authors have substantially addressed my major concerns in the revised manuscript. In particular, the addition of direct binding assays, including pull-down, SPR, and ELISA, now provides much stronger support for the proposed interaction between host-derived TNF-α and mosquito Wng. The revised data also better distinguish the roles of Wng and Grnd and strengthen the mechanistic link to the mTRAF3-mTAK1-REL2/TEP1 axis. Additional experiments addressing mosquito fitness, blood-feeding behavior, microbiota, hemocyte localization, and P. falciparum infection substantially improve the manuscript.

I am satisfied that the main scientific concerns have been adequately addressed. I would support publication after minor revision. The remaining issues are mainly textual and should be corrected before acceptance. The authors should carefully edit the manuscript for grammar, terminology, and typographical errors, and should avoid overstatement where mechanistic activation is inferred from genetic/functional assays rather than being directly measured biochemically.

Further comment:

After reading Referee #2's remaining concerns, I agree that several mechanistic conclusions in the manuscript remain

insufficiently supported by the current data. Although I still find the study interesting and potentially important, particularly the observation that host-derived TNF-α can influence mosquito susceptibility to Plasmodium infection, I agree with Referee #2 that some of the mechanistic interpretations should either be experimentally strengthened or substantially toned down.

In particular, the proposed link between TNF-α and suppression of the mosquito IMD pathway remains largely inferential. The current evidence appears to rely mainly on transcriptional changes and RNAi phenotypes, without direct demonstration of REL2 activation, cleavage or nuclear translocation. Therefore, I do not think the manuscript should make a strong conclusion that TNF-α directly suppresses IMD signaling unless additional evidence is provided. Alternatively, the authors could revise the wording to state that TNF-α deficiency is associated with changes in IMD-related immune markers and TEP1 activity.

I also agree that the hemocyte differentiation claims should be toned down. The current data based on hemocyte proportions do not seem sufficient to conclude that host-derived TNF-α impedes differentiation of prohemocytes into granulocytes and oenocytoids. A more cautious interpretation would be appropriate unless additional cell-identity markers or functional evidence are provided.

The potential systemic effects of TNF-α deficiency in mice also remain an important confounding issue. Because TNF-α deletion can alter inflammatory, metabolic and hormonal states during infection, the mosquito phenotypes may not reflect TNF-α alone. This point should be discussed more carefully, including the possible influence of insulin signaling and other host-derived blood factors. The cited interpretation of the Pakpour et al. insulin study should also be corrected if it is currently stated in the wrong direction.

Regarding ookinete transformation, I agree that the authors have not fully performed the specific experiment requested by Referee #2. However, I would not necessarily require this experiment if the authors avoid making strong claims about altered parasite transformation dynamics. If they wish to retain such claims, then quantification of zygotes, retorts and mature ookinetes at 16-20 h post-feeding would be needed.

Overall, my recommendation would be to request a further focused revision. As I noted in my previous review, the authors should avoid overstating mechanistic activation when pathway involvement is inferred from genetic or functional assays rather than being directly measured biochemically.

Dear Referees,

Thanks a lot for your comments, which would greatly improve our manuscript. We have responded to your comments point-by-point as following. We hope you will be satisfied with our responses.

Response to Referee #1

Thank you for the responses.

Response: Thanks a lot for your stratification with our revision**.**

Response to Referee #2

I appreciate the authors' efforts to address my previous comments. Although, the revised manuscript contains several interesting observations regarding the effect of host-derived TNF-α on mosquito susceptibility to Plasmodium infection, I still remain unconvinced that the mechanistic conclusions proposed by the authors are sufficiently supported by the presented data. In particular, several central claims regarding IMD signaling, hemocyte differentiation, and interpretation of pathway-specific effects remain largely inferential and, in some cases, inconsistent with the presented figures or prior literature. Given the EMBO reports' policy allowing only a single round of revisions, I am in the unfortunate position of having to reject the manuscript in its current form, for the following reasons:

Evidence supporting IMD-dependent regulation of TEP1 remains insufficient

1.The authors propose that host-derived TNF-α suppresses mosquito anti-Plasmodium immunity through inhibition of the IMD pathway, ultimately reducing TEP1 expression. Although the authors demonstrated the reduction of TEP1 active form following infection on TNF-α depleted mosquitoes, the evidence supporting the mechanistic link between TNF-α and IMD remains incomplete.

The manuscript does not directly demonstrate IMD pathway activation or suppression. In particular, no evidence is provided regarding:

- REL2 cleavage/activation or,
- REL2 nuclear translocation

The conclusions are therefore based primarily on transcriptional changes and RNAi phenotypes, which are insufficient to establish direct suppression of IMD signaling.

Response: Thanks for your comment**.** We acknowledge that experimental data documenting REL2 cleavage/activation or REL2 nuclear translocation would further strengthen our conclusions. Unfortunately, commercial antibodies targeting total and phosphorylated REL2 are currently unavailable, which prevents us from performing such assays. Consistent with similar published work in this field, conclusions are therefore drawn principally based on transcriptional profiling and RNAi phenotypic observations(Feng et al, 2021; Frolet et al, 2006; Estévez-Lao et al, 2025). To avoid overstatement of our conclusion, we have toned down our conclusion about the IMD pathway-mediated regulation of TEP1 expression thorough manuscript. These revision can be found in line 15-16, line 88, line 193-194, line 218-219, line 231-232, line 337, and line 1001-1002.

2.Ookinete transformation efficiency was not evaluated in vivo as requested

The authors state in their response that the in vivo effect of TNF-α deficiency on ookinete development was already addressed in Figure 2A-C by quantifying the ookinete counts in the

mosquito midguts or the expression levels of ookinete-specific genes in mosquitoes fed on WT or TNF-α -/- mice.

However, this is not equivalent to the experiment that was requested.

To properly assess in vivo transformation efficiency, mosquito midguts should be dissected approximately 16-18 h post blood feeding and the proportions of zygotes, retorts, and mature ookinetes within each blood bolus should be quantified relative to total parasite numbers. This would allow proper evaluation of whether TNF-α deficiency affects parasite developmental progression within the mosquito midgut.

The absence of significant differences in invaded ookinete numbers is partially reassuring; however, it does not fully exclude effects on parasite developmental dynamics prior to invasion.

Response: We apologize for misinterpreting your earlier comment. We agree that quantifying the relative proportions of zygotes, retorts and mature ookinetes within the mosquito midgut would better assess whether TNF modulates ookinete transformation efficiency. Nevertheless, our quantitative RT–PCR data and direct parasite counting both demonstrate that TNF exerts no significant impact on ookinete production, indicating TNF is unlikely to alter the process of ookinete transformation. However, we have revised the phrase “ookinete formation” to “ookinete stage” at line 159 to eliminate ambiguity.

3.Potential systemic confounding effects in TNF-α-deficient mice remain insufficiently addressed

Deletion of TNF-α is known to induce broad immunological and metabolic alterations in vertebrates. Therefore, the observed mosquito phenotypes may not solely reflect direct effects of TNF-α itself.

In particular, TNF-α deficiency during Plasmodium infection could alter the insulin signaling, a point that is especially relevant given previous studies linking vertebrate insulin signaling to increased permissiveness of Plasmodium infection in Anopheles stephensi. These potential systemic confounders should had been discussed more carefully. In this right, the authors mentioned in the discussion that "ingested human insulin was found to reduce P. falciparum burden...", which is actually the opposite of what Pakpour et al., 2012 demonstrated.

Response: We acknowledge that TNF exerts widespread immunological and metabolic effects in vertebrates. Accordingly, in our revised manuscript, we neutralized both TNF-αR1 and TNF-αR2 to rule out indirect TNF-mediated effects in mice. However, unforeseen off-target impacts of TNF on the mouse cannot be fully eliminated, and we have explicitly noted this technical limitation within the Discussion section. Please refer to line 382-388 of the revised manuscript.

We also apologize for the erroneous description of findings from Pakpour et al., 2012. Their work demonstrated that ingested human insulin elevates *Plasmodium falciparum* parasite burden. Now, we have rectified this error. Please refer to line 354 for details.

4.Hemocyte characterization and differentiation claims remain unconvincing

The images presented in Figure 5K do not convincingly resemble canonical granulocytes. The cells appear substantially larger (>40 μm) than expected granulocytes and contain abundant lipid droplets. These cells may instead represent fat body contaminants or potentially megacytes rather than granulocytes.

Furthermore, the statement:

"host-derived TNF-α impedes the differentiation of prohemocytes into granulocytes and oenocytoids" is not sufficiently supported by the presented data (Fig. 5N).

The authors are heavily relying on merely the proportions of different hemocyte subpopulations, therefore, the conclusions regarding hemocyte differentiation should be substantially toned down.

Response: We have carefully re-examined the image shown in Figure 5K and acknowledge that the large cell size shown is characteristic of megacytes. We further reviewed the original image for Figure 5K, which displays a typical granulocyte. Although megacytes represent a subpopulation of granulocytes, we plan to replace the current panel with the original granulocyte image. Please see the updated Figure 5Kprovided in the revised manuscript.

Regarding Figure 5N, we have toned down our concluding statement, as we lack direct experimental evidence to fully support the claim that host-derived TNF-α blocks prohemocyte differentiation into granulocytes and oenocytoids. Please refer to line 296-298 for details.

5.The explanation for discrepancies with previous studies remains insufficient

The authors propose that discrepancies between the current study and previous publications can be explained primarily by differences in TNF-α dose.

However, this explanation remains insufficient, particularly because contradictory phenotypes are observed following silencing of the same genes (Wgn and Grnd) across studies. It remains unclear how differences in ligand concentration alone would explain opposite phenotypic outcomes resulting from receptor silencing experiments.

Response: Thanks. We propose that the conflicting phenotype observed upon silencing the same genes (Wgn and Grnd) in our work versus prior publication can also be interpreted by difference in TNF-α dosage. In earlier studies, Wgn and Grnd knockdown was carried out following intrathoracic injection of 50 ng recombinant TNF. By contrast, our gene silencing assays were conducted after mosquitoes fed on parasite-infected mice, which supply TNF at physiological endogenous concentrations.

Additionally, the manuscript itself reports discrepancies with previous observations regarding STAT-A-dependent effects on parasite development. In the text the authors stated: " although it significantly increased the oocyst number in mosquitoes feeding on the infected control mice (Fig. 3C)". Gupta et al., showed that STAT-A silencing increases the numbers of P.berghei oocysts.

It appears as though the particular work has a lot of discrepancies with the current literature, which could be the result of the mosquito species or strain used.

Response: Thanks. Initially, we identified the STAT homologue of *Anopheles stephensi* via sequence homology searching and designated this gene STAT-A. However, a recent publication highlighted that proteins sharing analogous structures and functions often exhibit low sequence conservation, and recommended structural similarity-based searching for homologous protein identification(Wu et al, 2026). When we re-analyzed our data using the structural comparison workflow described in that study, we confirmed that *An. stephensi* harbours only a single STAT gene, in contrast to *An. gambiae*, which possesses two paralogs: STAT-A and STAT-B. As illustrated by our structural prediction results below, the STAT protein from *An. stephensi* exhibits far greater structural homology to STAT-B of *An. gambiae*. Now *STAT-A* in the text (line 200-201) and FigEV4C and Fig.3B-C has been changed into *STAT*.

We therefore agree with you that the inconsistent phenotypic outcomes between our work and prior literature can be largely attributed to the distinct mosquito species examined. Now, we have elaborated on this species-dependent discrepancy in the Discussion section in our revised manuscript. Please refer to line 407-411 for details.

Figure for referee with unpublished data has been removed upon request by the authors.

Finally, the authors did not address my previous suggestion regarding intrathoracic injection of recombinant TNF-α to directly test their proposed dose-dependent model in vivo. Such experiments would substantially strengthen the proposed explanation for the apparent discrepancies with previous studies.

Response: As raised in the reviewer's earlier comment, the original request was only to discuss the discrepancies between our findings and prior published work. Nevertheless, we acknowledge that directly validating our proposed dose-dependent model in vivo via intrathoracic delivery of recombinant TNF-α at varying concentrations would strengthen our arguments. As illustrated in the figure below, intrathoracic injection of 1 ng rTNF-α markedly elevated infection intensity, whereas intrathoracic delivery of 20 ng rTNF-α led to a significant reduction in infection intensity. Thus, our data does support the dual effect of dose-dependent TNF-α on malaria transmission.

Figure for referee with unpublished data has been removed upon request by the authors.

Minor comments

1.The citation "B et al., 1996" should be corrected to Lemaitre et al., 1996 (PMID: 8808632).

Response: Thanks. Now, we have amended the citation format for the reference "B et al., 1996" as you suggested.

2.Please carefully verify citation formatting throughout the manuscript according to EMBO reports standards.

Response: We have thoroughly reviewed and standardized all citation formatting across the entire

manuscript in strict accordance with the formatting guidelines of EMBO Reports.

3.Wengen nomenclature remains inconsistent throughout the manuscript and should be standardized.

Response: Thanks. As you suggested, we have carefully gone through the manuscript and standardized the nomenclature of Wengen as Wgn throughout the revised manuscript.

Response to Referee #3

The authors have substantially addressed my major concerns in the revised manuscript. In particular, the addition of direct binding assays, including pull-down, SPR, and ELISA, now provides much stronger support for the proposed interaction between host-derived TNF-α and mosquito Wng. The revised data also better distinguish the roles of Wng and Grnd and strengthen the mechanistic link to the mTRAF3-mTAK1-REL2/TEP1 axis. Additional experiments addressing mosquito fitness, blood-feeding behavior, microbiota, hemocyte localization, and P. falciparum infection substantially improve the manuscript.

I am satisfied that the main scientific concerns have been adequately addressed. I would support publication after minor revision. The remaining issues are mainly textual and should be corrected before acceptance. The authors should carefully edit the manuscript for grammar, terminology, and typographical errors, and should avoid overstatement where mechanistic activation is inferred from genetic/functional assays rather than being directly measured biochemically.

Response: We appreciate your positive assessment of our revised manuscript and the decision to request minor revisions. Following your guidance, we have thoroughly reviewed the full text and comprehensively fixed grammatical errors, inconsistent terminology, and typographical mistakes to the best of our ability. Moreover, we have toned down our conclusions regarding two key points: the IMD pathway-mediated regulation of TEP1 expression, and the suppression of prohemocyte differentiation into granulocytes and oenocytoids driven by host-derived TNF-α. All corresponding modifications are marked as yellow color in the revised manuscript for your reference.

Further comment:

After reading Referee #2's remaining concerns, I agree that several mechanistic conclusions in the manuscript remain insufficiently supported by the current data. Although I still find the study interesting and potentially important, particularly the observation that host-derived TNF-α can influence mosquito susceptibility to Plasmodium infection, I agree with Referee #2 that some of the mechanistic interpretations should either be experimentally strengthened or substantially toned down.

Response: Thank you very much for your feedback regarding Referee #2's concerns on our revised manuscript. We have adjusted the manuscript text in accordance with your instructions. Our detailed point-by-point responses are provided below.

In particular, the proposed link between TNF-α and suppression of the mosquito IMD pathway remains largely inferential. The current evidence appears to rely mainly on transcriptional changes and RNAi phenotypes, without direct demonstration of REL2 activation, cleavage or nuclear translocation. Therefore, I do not think the manuscript should make a strong conclusion that TNF-α directly suppresses IMD signaling unless additional evidence is provided. Alternatively, the

authors could revise the wording to state that TNF-α deficiency is associated with changes in IMD-related immune markers and TEP1 activity.

Response: Thank you. We acknowledge that our study lacks definitive evidence to confirm direct suppression of the mosquito IMD pathway by TNF-α, as well as IMD pathway-dependent regulation of TEP1 expression. Accordingly, we have rephrased our conclusions to only describe correlations between TNF-α depletion and altered levels of IMD-associated immune markers, IMD pathway activity, and TEP1 expression. These revisions can be found on line 218-219.

I also agree that the hemocyte differentiation claims should be toned down. The current data based on hemocyte proportions do not seem sufficient to conclude that host-derived TNF-α impedes differentiation of prohemocytes into granulocytes and oenocytoids. A more cautious interpretation would be appropriate unless additional cell-identity markers or functional evidence are provided.

Response: Thanks. Following your guidance, we have tone down the conclude that host-derived TNF-α impedes differentiation of prohemocytes into granulocytes and oenocytoids. Please refer to line 296-298 for reference.

The potential systemic effects of TNF-α deficiency in mice also remain an important confounding issue. Because TNF-α deletion can alter inflammatory, metabolic and hormonal states during infection, the mosquito phenotypes may not reflect TNF-α alone. This point should be discussed more carefully, including the possible influence of insulin signaling and other host-derived blood factors. The cited interpretation of the Pakpour et al. insulin study should also be corrected if it is currently stated in the wrong direction.

Response: Thank you very much for your insightful suggestion. We have now clarified in the Discussion section that indirect impacts of TNF-α on malaria transmission—potentially mediated by disrupted insulin signaling and altered production of other host-derived circulating factors—cannot be fully excluded. Please refer to line 382-388 for details.

Regarding ookinete transformation, I agree that the authors have not fully performed the specific experiment requested by Referee #2. However, I would not necessarily require this experiment if the authors avoid making strong claims about altered parasite transformation dynamics. If they wish to retain such claims, then quantification of zygotes, retorts and mature ookinetes at 16-20 h post-feeding would be needed.

Response: Thank you for your valuable suggestion. Our original intention was to state that TNF-α exerts no influence on ookinete generation rather than ookinete transformation. To eliminate ambiguity, we have revised the phrase “ookinete formation” to “ookinete stage” at line 159.

Overall, my recommendation would be to request a further focused revision. As I noted in my previous review, the authors should avoid overstating mechanistic activation when pathway involvement is inferred from genetic or functional assays rather than being directly measured biochemically.

Response: Thank you. Consistent with the explanations provided above, we have tempered several mechanistic conclusions where pathway engagement was only inferred from genetic or functional assays. We hope the revisions and responses meet your expectations.

Reference:

Estévez-Lao TY, Martin LE & Hillyer JF (2025) Activation of the immune deficiency pathway (IMD) reduces the mosquito heart rate via a nitric oxide-based mechanism. J Insect Physiol 161: 104738

Feng Y, Chen L, Gao L, Dong L, Wen H, Song X, Luo F, Cheng G & Wang J (2021) Rapamycin inhibits pathogen transmission in mosquitoes by promoting immune activation. PLOS Pathog 17: e1009353

Frolet C, Thoma M, Blandin S, Hoffmann JA & Levashina EA (2006) Boosting NF-κB-Dependent Basal Immunity of *Anopheles gambiae* Aborts Development of *Plasmodium berghei*. Immunity 25: 677–685

Wu W, Cui C, Zhu Y, Chen J, Zhuang Q, Wang Y, Liu Z, Gao H, Ou G-Z, Liu C, et al (2026) Structural genomics sheds light on protein functions and remote homologs across the insect tree of life. Cell Res 36: 411–425

2nd Revision - Editorial Decision 24th Aug 2026

Dear Prof. Xu

Thank you for the submission of your further revised manuscript to our editorial offices. I have now received the report from the referee that was asked to re-evaluate the study, you will find below. As you will see, the referee now supports the publication of the study in EMBO reports. However, the referee has remaining comments and suggestions to improve the manuscript which I ask you to do in a final revised manuscript. This requires mainly text changes. Please provide a final manuscript file where these are highlighted and also a final p-b-p-response to the remaining points of the referee.

I look forward to seeing the final revised version of your manuscript when it is ready. Please let me know if you have questions regarding the revision.

Best,

Achim Breiling
Senior Editor
EMBO Reports

Referee #3:

The authors have made substantial efforts to address the concerns raised during the previous rounds of review, and the manuscript has improved considerably. In particular, the additional experiments examining TNF-α binding to Wgn, Wgn versus Grnd specificity, the detection of host-derived TNF-α in mosquito hemolymph, and the P. falciparum membrane-feeding experiments have significantly strengthened the mechanistic and translational aspects of the study.

I also appreciate the authors' efforts to examine alternative explanations, including mosquito fitness, feeding behavior, microbiota composition, and parasite developmental stages, as well as the expanded analysis of the IMD-associated pathway. Overall, I believe that the major experimental concerns raised previously have now been substantially addressed.

However, a few interpretive and presentation issues still need clarification before the manuscript is suitable for acceptance.

The statement that TNF-α "activates mTRAF3" appears stronger than the experimental evidence currently supports. The authors demonstrate that mTRAF3 is functionally required for the TNF-α-dependent phenotype, since mTRAF3 knockdown abolishes the differences in parasite infection and downstream mTAK1, REL2, and TEP1 expression. However, I do not see a direct biochemical demonstration of mTRAF3 activation, such as receptor recruitment, altered protein localization, ubiquitination, or another activation-state measurement. The manuscript itself appropriately states in one location that TNF-α/Wgn signaling "may activate mTRAF3." I therefore suggest replacing definitive statements such as "TNF-α activates mTRAF3" with wording such as "TNF-α/Wgn signaling acts through mTRAF3" or "is mTRAF3-dependent" throughout the manuscript, unless additional direct evidence of mTRAF3 activation is available.

The wording "downregulating mTRAF3" should also be corrected. The presented experiments support a model in which mTRAF3 is required for the TNF-α-mediated suppression of mTAK1/REL2/TEP1 signaling, rather than demonstrating that TNF-α directly downregulates mTRAF3 itself. The section title and corresponding schematic should therefore be revised for consistency with the experimental data.
The proposed clinical implication of anti-TNF-α treatment should be substantially softened. The manuscript currently suggests that anti-TNF-α treatment "could be considered for clinical use in malaria patients" to prevent cerebral malaria, reduce transmission, and improve transmission-blocking efficacy. While the mouse ECM experiments and transmission studies are interesting and potentially important, these data do not yet establish the safety or therapeutic efficacy of systemic TNF-α blockade in human malaria. I suggest revising this section to state that modulation of TNF-α signaling may represent a potential avenue for future investigation, but that further studies are required before any clinical application can be considered.

Some of the mechanistic language remains somewhat teleological. For example, the manuscript states that malaria parasites "exploit" host TNF-α signaling to enhance transmission. The present data demonstrate that host-derived TNF-α creates a physiological environment that favors parasite transmission, but they do not directly demonstrate that the parasite actively manipulates this pathway. A more cautious formulation, such as "host-derived TNF-α signaling favors malaria parasite transmission" or "can be utilized by the parasite to facilitate transmission," would be preferable.

The manuscript would benefit from one additional careful language edit. Several grammatical and stylistic issues remain throughout the text, including phrases such as "super higher dose," "the silence of either Wgn or Grnd," and multiple subject-verb agreement errors. These do not affect the overall scientific conclusions but should be corrected before publication.

In summary, I believe that the authors have satisfactorily addressed the major experimental concerns raised in the previous rounds of review. The remaining issues are primarily related to interpretation and wording rather than the need for additional major experiments. I therefore recommend minor revision. If the authors appropriately temper the mechanistic and clinical claims outlined above and carefully edit the language, I would be comfortable supporting acceptance of the manuscript.

Dear Referee,

Thanks a lot for your comments, which would greatly improve our manuscript. We have responded to your comments point-by-point as following. We hope you will be satisfied with our responses.

Response to Referee #3:

The authors have made substantial efforts to address the concerns raised during the previous rounds of review, and the manuscript has improved considerably. In particular, the additional experiments examining TNF-α binding to Wgn, Wgn versus Grnd specificity, the detection of host-derived TNF-α in mosquito hemolymph, and the P. falciparum membrane-feeding experiments have significantly strengthened the mechanistic and translational aspects of the study.

I also appreciate the authors' efforts to examine alternative explanations, including mosquito fitness, feeding behavior, microbiota composition, and parasite developmental stages, as well as the expanded analysis of the IMD-associated pathway. Overall, I believe that the major experimental concerns raised previously have now been substantially addressed.

Response**:** Thanks a lot for your appreciation of our revision.

However, a few interpretive and presentation issues still need clarification before the manuscript is suitable for acceptance.

Response**:** Thanks for your comments. Now, we have clarified all issues as you pointed as following.

Q1. The statement that TNF-α "activates mTRAF3" appears stronger than the experimental evidence currently supports. The authors demonstrate that mTRAF3 is functionally required for the TNF-α-dependent phenotype, since mTRAF3 knockdown abolishes the differences in parasite infection and downstream mTAK1, REL2, and TEP1 expression. However, I do not see a direct biochemical demonstration of mTRAF3 activation, such as receptor recruitment, altered protein localization, ubiquitination, or another activation-state measurement. The manuscript itself appropriately states in one location that TNF-α/Wgn signaling "may activate mTRAF3." I therefore suggest replacing definitive statements such as "TNF-α activates mTRAF3" with wording such as "TNF-α/Wgn signaling acts through mTRAF3" or "is mTRAF3-dependent" throughout the manuscript, unless additional direct evidence of mTRAF3 activation is available.

Response: Thanks. We agree that our current experimental evidence only demonstrates a functional requirement for mTRAF3 in mediating the TNF-α/Wgn signaling cascade, rather than a direct biochemical activation. As you suggested, we have replaced statement of "TNF-α activates mTRAF3" with "TNF-α/Wgn signaling acts through mTRAF3" throughout the manuscript. These revisions can be found on line 14-15, 337, 1049.

Q2. The wording "downregulating mTRAF3" should also be corrected. The presented experiments support a model in which mTRAF3 is required for the TNF-α-mediated suppression of mTAK1/REL2/TEP1 signaling, rather than demonstrating that TNF-α directly downregulates mTRAF3 itself. The section title and corresponding schematic should therefore be revised for consistency with the experimental data.

Response: Thanks. We have adjusted the corresponding text and section title in manuscript as you

suggested. These revisions can be found on line 235-236, 258-259,1032-1033. Furthermore, the solid line of arrow between TNF-α and mTRAF3 in Figure 4N, between Wng and mTRAF3 in Figure 5Q and synopsis image, have been replaced a dashed line.

Q3. The proposed clinical implication of anti-TNF-α treatment should be substantially softened. The manuscript currently suggests that anti-TNF-α treatment "could be considered for clinical use in malaria patients" to prevent cerebral malaria, reduce transmission, and improve transmission-blocking efficacy. While the mouse ECM experiments and transmission studies are interesting and potentially important, these data do not yet establish the safety or therapeutic efficacy of systemic TNF-α blockade in human malaria. I suggest revising this section to state that modulation of TNF-α signaling may represent a potential avenue for future investigation, but that further studies are required before any clinical application can be considered.
Response: We sincerely thank you for your constructive comments regarding the clinical implications of our findings. we have carefully revised the manuscript accordingly. These revisions can be found on line 427-429.

Q4. Some of the mechanistic language remains somewhat teleological. For example, the manuscript states that malaria parasites "exploit" host TNF-α signaling to enhance transmission. The present data demonstrate that host-derived TNF-α creates a physiological environment that favors parasite transmission, but they do not directly demonstrate that the parasite actively manipulates this pathway. A more cautious formulation, such as "host-derived TNF-α signaling favors malaria parasite transmission" or "can be utilized by the parasite to facilitate transmission," would be preferable.
Response: Thank you very much for your suggestion. We fully agree that our data demonstrate a favorable physiological environment created by host-derived TNF-α, rather than active manipulation by the parasite. We have revised the wording on lines 21-22, 341-342 as you suggested.

Q5. The manuscript would benefit from one additional careful language edit. Several grammatical and stylistic issues remain throughout the text, including phrases such as "super higher dose," "the silence of either Wgn or Grnd," and multiple subject-verb agreement errors. These do not affect the overall scientific conclusions but should be corrected before publication.
Response: We sincerely appreciate your constructive suggestion concerning language editing. We have now thoroughly revised the language throughout the entire manuscript with assistance from artificial intelligence. All revisions are highlighted in the revised manuscript, and we have noted the use of artificial intelligence for language editing in the Acknowledgements section.

In summary, I believe that the authors have satisfactorily addressed the major experimental concerns raised in the previous rounds of review. The remaining issues are primarily related to interpretation and wording rather than the need for additional major experiments. I therefore recommend minor revision. If the authors appropriately temper the mechanistic and clinical claims outlined above and carefully edit the language, I would be comfortable supporting acceptance of the manuscript.
Response: Thanks a lot for your appreciation and comments, which will greatly improve our manuscript. Hopefully, you will be satisfied with our responses.

Prof. Wenyue Xu
Army Medical University (Third Military Medical University)
Department of Pathogenic Biology
Gaotanyan Zhengjie 30, Shapingba District
Chongqing
China

Dear Prof. Xu,

Thank you for the submission of your final revised manuscript to our editorial offices. I now went through this and your final p-b-p-response and consider the remaining points of referee #3 and all editorial requests as adequately addressed.

I am thus very pleased to accept your manuscript for publication in the next available issue of EMBO reports. Thank you for your contribution to our journal.

Your manuscript will be processed for publication by EMBO Press. It will be copy edited and you will receive page proofs prior to publication. Please note that you will be contacted by Springer Nature Author Services to complete licensing and payment information.

You may qualify for financial assistance for your publication charges - either via a Springer Nature fully open access agreement or an EMBO initiative. Check your eligibility: https://link.springer.com/journal/44319/how-to-publish-with-us

Should you be planning a Press Release on your article, please notify the production team via this form in order to coordinate publication and release dates: https://forms.cloud.microsoft/e/eH4A8JVP6g

If you have any questions, please do not hesitate to contact the Editorial Office. Thank you for your contribution to EMBO Reports.

Yours sincerely,

Achim Breiling
Senior Editor
EMBO Reports

>>> Please note that it is EMBO Reports policy for the transcript of the editorial process (containing referee reports and your response letter) to be published as an online supplement to each paper. If you do NOT want this, you will need to inform the Editorial Office via email immediately. More information is available here: https://link.springer.com/partners/embo-press/editorial-policies#Peer%20review

EMBO Press Author Checklist

Corresponding Author Name: Wenyue Xu	
Journal Submitted to: EMBO Reports	
Manuscript Number: EMBOR-2025-63348V2	

USEFUL LINKS FOR COMPLETING THIS FORM
The EMBO Journal - Author Guidelines
EMBO Reports - Author Guidelines
Molecular Systems Biology - Author Guidelines
EMBO Molecular Medicine - Author Guidelines

Reporting Checklist for Life Science Articles (updated January
This checklist is adapted from Materials Design Analysis Reporting (MDAR) Checklist for Authors. MDAR establishes a minimum set of requirements in transparent reporting in the life sciences (see Statement of Task: 10.31222/osf.io/9sm4x). Please follow the journal's guidelines in preparing your
Please note that a copy of this checklist will be published alongside your article.

Abridged guidelines for figures
1. Data
The data shown in figures should satisfy the following conditions:
- the data were obtained and processed according to the field's best practice and are presented to reflect the results of the experiments in an accurate and unbiased manner.
- ideally, figure panels should include only measurements that are directly comparable to each other and obtained with the same assay.
- plots include clearly labeled error bars for independent experiments and sample sizes. Unless justified, error bars should not be shown for technical
- if n<5, the individual data points from each experiment should be plotted. Any statistical test employed should be justified.
- Source Data should be included to report the data underlying figures according to the guidelines set out in the authorship guidelines on Data

2. Captions
Each figure caption should contain the following information, for each panel where they are relevant:
- a specification of the experimental system investigated (eg cell line, species name).
- the assay(s) and method(s) used to carry out the reported observations and measurements.
- an explicit mention of the biological and chemical entity(ies) that are being measured.
- an explicit mention of the biological and chemical entity(ies) that are altered/varied/perturbed in a controlled manner.
- the exact sample size (n) for each experimental group/condition, given as a number, not a range;
- a description of the sample collection allowing the reader to understand whether the samples represent technical or biological replicates (including how many animals, litters, cultures, etc.).
- a statement of how many times the experiment shown was independently replicated in the laboratory.
- definitions of statistical methods and measures:
  - common tests, such as t-test (please specify whether paired vs. unpaired), simple χ2 tests, Wilcoxon and Mann-Whitney tests, can be unambiguously identified by name only, but more complex techniques should be described in the methods section;
  - are tests one-sided or two-sided?
  - are there adjustments for multiple comparisons?
  - exact statistical test results, e.g., P values = x but not P values < x;
  - definition of 'center values' as median or average;
  - definition of error bars as s.d. or s.e.m.

Please complete ALL of the questions below.
Select "Not Applicable" only when the requested information is not relevant for your study.

Materials

Newly Created Materials	Information included in the manuscript?	In which section is the information available? (Reagents and Tools Table, Materials and Methods, Figures, Data Availability Section)
New materials and reagents need to be available; do any restrictions apply?	Not Applicable	

Antibodies	Information included in the manuscript?	In which section is the information available? (Reagents and Tools Table, Materials and Methods, Figures, Data Availability Section)
For **antibodies** provide the following information:<br>- Commercial antibodies: RRID (if possible) or supplier name, catalogue number and or/clone number<br>- Non-commercial: RRID or citation	Yes	Reagents and Tools Table

DNA and RNA sequences	Information included in the manuscript?	In which section is the information available? (Reagents and Tools Table, Materials and Methods, Figures, Data Availability Section)
**Short novel DNA or RNA including primers, probes**: provide the sequences.	Yes	Reagents and Tools Table

Cell materials	Information included in the manuscript?	In which section is the information available? (Reagents and Tools Table, Materials and Methods, Figures, Data Availability Section)
**Cell lines:** Provide species information, strain. Provide accession number in repository **OR** supplier name, catalog number, clone number, and/**OR** RRID.	Not Applicable	
**Primary cultures:** Provide species, strain, sex of origin, genetic modification status.	Not Applicable	
Report if the cell lines were recently **authenticated** (e.g., by STR profiling) and tested for mycoplasma contamination.	Not Applicable	

Experimental animals	Information included in the manuscript?	In which section is the information available? (Reagents and Tools Table, Materials and Methods, Figures, Data Availability Section)
**Laboratory animals or Model organisms:** Provide species, strain, sex, age, genetic modification status. Provide accession number in repository **OR** supplier name, catalog number, clone number, **OR** RRID.	Yes	Reagents and Tools Table
**Animal observed in or captured from the field:** Provide species, sex, and age where possible.	Not Applicable	
Please detail **housing and husbandry conditions**.	Yes	Materials and methods

Plants and microbes	Information included in the manuscript?	In which section is the information available? (Reagents and Tools Table, Materials and Methods, Figures, Data Availability Section)
**Plants:** provide species and strain, ecotype and cultivar where relevant, unique accession number if available, and source (including location for collected wild specimens).	Not Applicable	
**Microbes:** provide species and strain, unique accession number if available, and source.	Not Applicable	

Human research participants	Information included in the manuscript?	In which section is the information available? (Reagents and Tools Table, Materials and Methods, Figures, Data Availability Section)
If collected and within the bounds of privacy constraints report on age, sex and gender or ethnicity for all study participants.	Not Applicable	

Core facilities	Information included in the manuscript?	In which section is the information available? (Reagents and Tools Table, Materials and Methods, Figures, Data Availability Section)
If your work benefited from core facilities, was their service mentioned in the acknowledgments section?	Not Applicable	

Design

Study protocol	Information included in the manuscript?	In which section is the information available? (Reagents and Tools Table, Materials and Methods, Figures, Data Availability Section)
If study protocol has been **pre-registered, provide DOI in the manuscript**. For clinical trials, provide the trial registration number **OR** cite DOI.	Not Applicable	
Report the **clinical trial registration number** (at ClinicalTrials.gov or equivalent), where applicable.	Not Applicable	

Laboratory protocol	Information included in the manuscript?	In which section is the information available? (Reagents and Tools Table, Materials and Methods, Figures, Data Availability Section)
Provide DOI OR other citation details if **external detailed step-by-step protocols** are available.	Not Applicable	

Experimental study design and statistics	Information included in the manuscript?	In which section is the information available? (Reagents and Tools Table, Materials and Methods, Figures, Data Availability Section)
Include a statement about **sample size** estimate even if no statistical methods were used.	Yes	Figure Legends
Were any steps taken to minimize the effects of subjective bias when allocating animals/samples to treatment (e.g. **randomization procedure**)? If yes, have they been described?	Yes	Materials and methods
Include a statement about **blinding** even if no blinding was done.	Yes	Materials and methods
Describe **inclusion/exclusion criteria** if samples or animals were excluded from the analysis. Were the criteria pre-established?<br><br>If sample or data points were omitted from analysis, report if this was due to attrition or intentional exclusion and provide justification.	Not Applicable	
For every figure, are **statistical tests** justified as appropriate? Do the data meet the assumptions of the tests (e.g., normal distribution)? Describe any methods used to assess it. Is there an estimate of variation within each group of data? **I**s the variance similar between the groups that are being statistically compared?	Yes	Materials and methods

Sample definition and in-laboratory replication	Information included in the manuscript?	In which section is the information available? (Reagents and Tools Table, Materials and Methods, Figures, Data Availability Section)
In the figure legends: state number of times the experiment was **replicated** in laboratory.	Yes	Figure Legends
In the figure legends: define whether data describe **technical or biological replicates**.	Yes	Figure Legends

Ethics

Ethics	Information included in the manuscript?	In which section is the information available? (Reagents and Tools Table, Materials and Methods, Figures, Data Availability Section)
Studies involving **human participants**: State details of **authority granting ethics approval** (IRB or equivalent committee(s), provide reference number for approval.	Not Applicable	
Studies involving **human participants**: Include a statement confirming that **informed consent** was obtained from all subjects and that the experiments conformed to the principles set out in the WMA Declaration of Helsinki and the Department of Health and Human Services Belmont Report.	Not Applicable	
Studies involving **human participants:** For publication of **patient photos**, include a statement confirming that consent to publish was obtained.	Not Applicable	
Studies involving experimental **animals**: State details of **authority granting ethics approval** (IRB or equivalent committee(s), provide reference number for approval. Include a statement of compliance with ethical regulations.	Yes	Materials and methods
Studies involving **specimen and field samples:** State if relevant **permits** obtained, provide details of authority approving study; if none were required, explain why.	Not Applicable	

Dual Use Research of Concern (DURC)	Information included in the manuscript?	In which section is the information available? (Reagents and Tools Table, Materials and Methods, Figures, Data Availability Section)
Could your study fall under dual use research restrictions? Please check biosecurity documents and list of **select agents and toxins** (CDC): https://www.selectagents.gov/sat/list.htm	Not Applicable	
If you used a select agent, is the security level of the lab appropriate and reported in the manuscript?	Not Applicable	
If a study is subject to dual use research of concern regulations, is the name of the **authority granting approval and reference number** for the regulatory approval provided in the manuscript?	Not Applicable	

Reporting

The MDAR framework recommends adoption of discipline-specific guidelines, established and endorsed through community initiatives. Journals have their own policy about requiring specific guidelines and recommendations to complement MDAR.

Adherence to community standards	Information included in the manuscript?	In which section is the information available? (Reagents and Tools Table, Materials and Methods, Figures, Data Availability Section)
State if relevant guidelines or checklists (e.g., **ICMJE, MIBBI, ARRIVE, PRISMA**) have been followed or provided.	Not Applicable	
For **tumor marker prognostic studies,** we recommend that you follow the **REMARK** reporting guidelines (see link list at top right). See author guidelines, under 'Reporting Guidelines'. Please confirm you have followed these guidelines.	Not Applicable	
For **phase II and III randomized controlled trials**, please refer to the **CONSORT** flow diagram (see link list at top right) and submit the CONSORT checklist (see link list at top right) with your submission. See author guidelines, under 'Reporting Guidelines'. Please confirm you have submitted this list.	Not Applicable	

Data Availability

Data availability	Information included in the manuscript?	In which section is the information available? (Reagents and Tools Table, Materials and Methods, Figures, Data Availability Section)
Have **primary datasets** been deposited according to the journal's guidelines (see 'Data Deposition' section) and the respective accession numbers provided in the Data Availability Section?	Yes	Data availability
Were **human clinical and genomic datasets** deposited in a public access-controlled repository in accordance to ethical obligations to the patients and to the applicable consent agreement?	Not Applicable	
Are **computational models** that are central and integral to a study available without restrictions in a machine-readable form? Were the relevant accession numbers or links provided?	Not Applicable	
If publicly available data were reused, provide the respective **data citations in the reference list**.	Not Applicable	
